# In Search of the Most Significant Potential G-Quadruplexes in SARS-CoV-2 RNA: Genomic Analysis

**DOI:** 10.3390/v18020253

**Published:** 2026-02-16

**Authors:** Margarita Zarudnaya, Ivan Voiteshenko, Vasyl Hurmah, Tetiana Shyryna, Alex Nyporko, Maksym Platonov, Szczepan Roszak, Bakhtiyor Rasulev, Karina Kapusta, Leonid Gorb

**Affiliations:** 1 Department of Molecular and Quantum Biophysics, Institute of Molecular Biology and Genetics, National Academy of Sciences of Ukraine, 150, Akademika Zabolotnoho Str., 03143 Kyiv, Ukraine; mzar@ukr.net (M.Z.); isvoiteshenko@knu.ua (I.V.); vhurmach@gmail.com (V.H.); t.shyryna@i.ua (T.S.); platon1971@gmail.com (M.P.); 2 Educational and Scientific Institute of High Technologies, Taras Shevchenko National University of Kyiv, 60 Volodymyrska Street, 01033 Kyiv, Ukraine; a_nyporko@knu.ua; 3 Faculty of Chemistry, University of Wrocław, 50370 Wrocław, Poland; szczepan.roszak@pwr.edu.pl; 4 Department of Coatings and Polymeric Materials, North Dakota State University, NDSU Department 2510, P.O. Box 6050, Fargo, ND 58108, USA; bakhtiyor.rasulev@ndsu.edu; 5 Department of Chemistry and Physics, Tougaloo College, Tougaloo, MS 39174, USA; karina.kapusta@icnanotox.org

**Keywords:** SARS-CoV-2 genome, G-quadruplexes, RNA secondary structure prediction, mutational analysis, docking, molecular dynamics simulation

## Abstract

G-quadruplexes (G4s) are emerging as potential antiviral targets. SARS-CoV-2 genomic RNA contains 42 G-rich regions harboring putative G-quadruplex-forming sequences (PQSs). Here, we performed a systematic genomic and structural analysis of SARS-CoV-2 PQSs. It was proposed that non-G-tetrads or different triads may stabilize most G4s in this RNA. Many G4s may include the most stable U·A-U triad. Several G-quadruplexes may be significantly stabilized by 3′ U-tetrad. Large-scale mutational analysis of RNA structural elements containing PQSs showed that most PQSs are highly conserved, while persistent G4-destroying mutations were observed only for one PQS and were transient for two others. Based on G4 position and structural context, we propose that: (i) G4 370 in nsp1 may contribute to cap-independent translation initiation; (ii) certain putative G4s in different genes may assist in co-translational folding of viral proteins; (iii) G4 13385, located upstream of the frameshift stimulation element, may promote formation of a pseudoknot competent for −1 frameshifting. For putative G4s at positions 3467, 13385 and 28903, we analyzed binding to 13 compounds by molecular docking and selected four candidates for molecular dynamics simulations. The ligand EKM emerged as a promising antiviral candidate due to its specific binding to G4 3467.

## 1. Introduction

RNA and DNA G-quadruplexes (G4s) are four-stranded structures built from stacks of at least two G-tetrads stabilized by Hoogsteen hydrogen bonds and monovalent cations. These non-canonical structures, which regulate diverse cellular and viral processes (for review, see [1,2,3,4,5,6,7,8]), are increasingly viewed as attractive therapeutic targets or agents [9,10,11], particularly in the case of COVID-19 caused by the SARS-CoV-2 virus. This virus has the ~30 kb positive-sense RNA genome which contains 47 putative G-quadruplex-forming sequences (PQSs) [12,13]. All these PQSs can form G4s with only two G-tetrads, implying relatively low stability. It was suggested that highly stable G-quadruplexes can be necessary for the inhibition of specific cellular processes, while low-stability G4s can serve as switchable and tunable motifs [14].

To date, the precise functions of individual RNA G4s in the SARS-CoV-2 genomic RNA (gRNA) remain unresolved. In principle, rG4s may up- or downregulate gene expression via at least three non-exclusive mechanisms: (i) acting as kinetic barriers to translocating proteins or protein machines (in particular, ribosomes); (ii) remodeling local secondary structure and thereby masking or exposing regulatory elements; and (iii) recruiting rG4-binding proteins that further modulate RNA fate [15]. At least, G4s in SARS-CoV-2 may slow translation of large proteins to provide additional time for their proper folding (such a function for RNA G-quadruplexes was proposed in [16]) or can attenuate replication to escape cell immune response [17,18].

The 47 PQSs in SARS-CoV-2 gRNA are located in 39 G-rich sequence (GRS) regions, since some of them overlap. The formation of G-quadruplexes by nine PQSs has been investigated in several works [13,17,18,19,20,21,22,23,24,25,26,27,28] using different biophysical assays, mainly CD (circular dichroism) spectroscopy and fluorescence (Table S1). By these main assays, Razzak et al. [27] investigated 16 more PQSs. Detailed atomistic structures have been obtained for only three PQSs through molecular dynamics and quantum chemical calculations [27,29,30,31,32].

For four PQSs, experimental studies have reported conflicting results regarding G4 formation (Table S1). These are PQSs located at positions 8687, 24268, 25197 and 28903 (further, for brevity, PQS or G4 at position N will be referred to as PQS N or G4 N). The discrepancy between the results can be due to different experimental conditions, the peculiarities of the methods used, and possible formation of different structures by PQSs. For example, ThT (Thioflavin T) fluorescence intensities were found to be similar for PQSs 644, 24215 and 24268 in Cui et al. [22], while they were significantly lower for PQSs 24215 and 24268 than for 644 in Razzak et al. [27]. However, in both works, the highest intensity was observed for PQS 13385 (significantly higher than for PQS 644) and the lowest for 25197. In these studies, both the assay conditions and the PQSs differed. In [22], PQSs contained flanking nucleotides that could have influenced the assay results. Moreover, the results of the fluorescence assay depend on fluorescent G4-specific targeting compounds. Contrary to ThT, NMM (N-methyl mesophorphyrin) fluorescence intensities for PQS 644 were higher than for 13385 [18]. CD assays do not clearly confirm the formation of G-quadruplexes but provide information on PQS folding state [24]

Conflicting results in the case of PQS 28903 may be due to its capability to form different structures. Firstly, it was suggested that it forms a hairpin rather than a G-quadruplex [13,19,24]. Qin et al. [18] reported ΔG values for PQSs 644, 1574, 3467, 13385 and 28903 at 25 °C. These values are in the range of −1.8 kcal/mol −3.1 kcal/mol (−2.6 kcal/mol for PQS 28903), and they will be higher at physiological temperature. According to our prediction of the secondary structure of PQS 28903 with flanking nucleotides (see Section 3.1), ΔG of the hairpin formed by it is −2.5 kcal/mol at 37 °C (Table S2); i.e., formation of the hairpin by PQS 28903 will be more preferable than the formation of G-quadruplex or will compete with its formation. Likewise, the hairpin formed by PQS 25197 with ΔG = −5.8 kcal/mol (Table S2) will be the dominant structure, which is in accordance with the experimental results described above.

Secondly, PQS 28903 can form dimeric quadruplex [33] and G-triplex [34]. In addition, multimeric rather than monomolecular G4s may form at high PQS concentrations. Formation of exactly monomolecular G4s was confirmed only for single PQSs. Thus, several different methods are needed to test whether the PQS forms G-quadruplex. In this regard, the formation of G4s by PQSs 644, 1574, 3467 and 13385 is most reliable (Table S1). Moreover, involvement of these G4s in regulating SARS-CoV-2 life cycle was confirmed by Qin et al. [18]. All these PQSs are highly conserved (for example, Ref. [27]) despite the high mutability of the SARS-CoV-2 genome

According to results of mutational analysis conducted in [27] for 25 potential G4 sequences, most PQSs are also highly conserved (mutation rate < 1%), but three PQSs, including 28903, showed very high mutation rates (90–100%). Recently, we separately searched for mutations in this PQS and in the domain containing it across different countries and years [32]. Frequent mutations within GG repeats in PQS 28903 were detected only in 2021–2022 but disappeared in 2023–2024, suggesting that G4-destroying mutations in it were not tolerated in the long term. Instead, a stable triple mutation shifted PQS location from the 5′ arm of a long hairpin to two shorter hairpins at the top of the domain containing it, potentially facilitating its interaction with cellular factors and proteins.

It should be noted that in the literature, the search for PQSs in SARS-CoV-2 was usually first performed using Quadruplex Forming G-Rich Sequences (QGRS) Mapper software (Mapper web server, version 2006, Ramapo College, USA) [35] with different parameters: in particular, with maximal PQS length of 30 nucleotides (nts) or 45 nts and loop size range of 0–12, 1–15 or 0–36 [13,18,22,23,25,27]. The PQSs found were then analyzed by additional algorithms (in particular, G4Hunter [36]) to evaluate the G4 folding capability considering the consecutive G over consecutive C ratio. However, the QGRS algorithm is not rigorous [37], not only because it does not take into account the G and C content but also because it does not take into consideration the structural features of G-quadruplexes. This may be the reason why certain PQSs do not agree with this algorithm and indeed form G-quadruplexes [37]. Neighboring (or/and internal) non-G residues in PQSs can form tetrads or triads stabilizing G-quadruplexes [38,39,40,41,42,43] that are not taken into account in the algorithms used. Furthermore, internal loops in G4s can form hairpins stabilizing them [44].

Therefore, in this work, we searched for PQSs via in-house analysis. We first identified the GG tracts using a simple computer search function and then manually inspected all GG-rich regions, assessing the possibility of G4 stabilization by non-G-tetrads, triads and base pairs. The clustering of GG tracts is clearly visible in RNA sequences. As a result, we identified all PQSs reported in the literature, as well as additional ones. We assessed the influence of GC content on the formation of G-quadruplexes by directly determining the change in free energy (ΔG) of the hairpin formed by PQSs. We included the flanking nucleotides, since they may additionally stabilize the hairpin. The influence of longer tracts encompassing the PQSs will depend not only on their sequence and length but also on their secondary structure, which algorithms do not account for. To set the PQS location, we predicted the secondary structure of the SARS-CoV-2 genomic RNA region containing it.

Thus, to better understand which SARS-CoV-2 G4s are most likely to be biologically relevant and to serve as antiviral targets, here we undertook a comprehensive analysis that integrates: (i) G4 stabilization by non-G-tetrads, triads and base-pairs; (ii) explicit competition between rG4 and minimum-free-energy hairpins; (iii) the impact of frequent mutations on the sequence and location of PQSs; and (iv) assumptions about the functions of some quadruplexes in the cell. For three PQSs, we studied their interactions with several stabilizing ligands using molecular dynamics simulations.

## 2. Materials and Methods

Sequences of the regions encompassing PQSs in SARS-CoV-2 genomes were extracted from the GISAID database gisaid.org (accessed on 09 10 2025). Mutational analysis was performed using the author’s software algorithm implemented in Python version 3.9.21. The maximum number of genomes from one country in one year was limited to the 10,000 most complete genomes as defined by GISAID: genomes >29,000 bp are considered complete and assigned a high-coverage label of <1% Ns (undetermined bases).

The secondary structure of SARS-CoV-2 regions containing PQSs has been predicted by the UNAFold program (Unified Nucleic Acid Folding, The RNA Institute, University at Albany, New York, USA) [45] for genome Wuhan-Hu-1 (accession number NC_045512, reference sequence). We used 15% suboptimality and window 0 for the automatic folding of RNA fragments. Additionally, we included the flanking nucleotides in all RNA fragments studied and applied folding constraints that prevented these nucleotides from participating in base pairing. In cases of separated hairpins, we included more than one nucleotide upstream and downstream of the hairpin if the single flanking nucleotides could form a base pair.

### 2.1. Molecular Docking Simulation

The Glide (grid-based ligand docking with energetics) tool, part of Maestro interface of Schrödinger suite of programs [46], was used. During the simulation, the receptor remains rigid, and the binding compounds are flexible. For molecular screening, 13 compounds (PDB Ids: A1AEC, A1AED, A1AEE, A1AEF, A1BC9, EKJ, EKM, J0D, POH, R14, TFX, V5Z and VK0) with known activity were selected. All the compounds were selected based on analysis of the PDB [47] and ChEMBL [https://www.ebi.ac.uk/chembl/ (accessed on 13 06 2025)] databases. All the simulations were done using the SP (standard precision) docking method. In each case, the best five conformations of each investigated compound were selected for visual inspection.

### 2.2. MD Simulation

The MD simulation was performed to estimate the time stability of the obtained RNA structures. The calculations were done with Gromacs 2021 software [48] using force field Charmm36 [49]. In the case of the RNA G-quadruplex, K^+^ was inserted within the quadruplex structure. Each system was placed into the center of a periodic triclinic box with the next filling by SPC/E water molecules. A minimum 1.5 nm distance was maintained between the nearest atom of the RNA and the edge of the simulation box so that the RNA could fully immerse in water and rotate freely. Before conducting any evaluations, each MD trajectory was subjected to a rotation and translation fit to a reference RNA structure. This step was carried out to eliminate translational and rotational motions of the RNA molecules, allowing subsequent analyses to concentrate on internal molecular motions and interaction. To neutralize the system and mimic the cellular environment (pH 7), K^+^ and Cl^−^ ions were added to bring the ionic concentration to 150 mM. In this process, the solvent molecules are randomly replaced by monoatomic ions. The systems obtained were energy-minimized via the steepest descent algorithm (the maximum number of steps was 50,000) and equilibrated in two stages. The stage of NVT equilibration was performed at 1 ns using a V-rescale thermostat, and the second NPT equilibration of 1 ns was performed appropriately with the same thermostat and a Berendsen barostat. After that, the MD simulations were launched within 100 ns to all the complexes, and then for some that were selected, the MD simulation was extended to 500 ns. All calculations were done at a temperature of 35 °C and constant atmospheric pressure.

### 2.3. gmx_MMPBSA Calculation

The MM/PBSA (molecular mechanics/Poisson–Boltzmann surface area) [50,51,52] and complex binding energy (Equation (1)) were determined using gmx_MMPBSA (version 1.4.1, University of Medellín, University of Calgary, University of Toronto, Colombia/Canada) [52] software tool.


G_binding_ = ΔE_MM_ + ΔG_ps_ + ΔG_nps_ − TΔS (1)

Here, ∆E_MM_ is the molecular mechanics contribution in a vacuum and is the sum of ΔE_vdW_ (van der Waals’ interaction energy) and ΔE_EE_ (electrostatic interaction). ∆G_ps_ is the polar solvation free energy, ∆G_nps_ is the non-polar solvation free energy, and –TΔS is the entropic contribution.

## 3. Results and Discussion

### 3.1. Genome-Wide Search for SARS-CoV-2 PQSs

Results of our search for PQSs are presented in Table S2A,B for G4s in SARS-CoV-2 positive-sense and negative-sense gRNAs, respectively. We presented all PQSs with potential tetrads consisting of A and U bases, while out of the PQSs with triads, we selected only those with a canonical U·A-U triad, which is, along with triad C^+^·G-C, the most stable [53]. Since triplexes can exist as independent structures (for example, in [34]), they can be formed by RNA regions containing only three GG repeats. Therefore, we also presented the sequences of the shortest putative G-triplexes in Table S2C.

We paid special attention to putative 3′ U·U·U·U tetrad in G-quadruplexes. This tetrad significantly stabilizes G4s consisting of four chains [54,55,56,57]; in particular, it increases the melting temperature (T_m_) of G4 formed by the human telomere RNA sequence r(UAGGGU) by 29 °C [55]. Possibly, this tetrad may stabilize monomolecular G-quadruplexes as well.

In the positive-sense strand, we identified 133 PQSs (Table S2A) in 42 G-rich sequences (GRSs)—almost three times more PQSs than published in the literature. From these, we have selected 23 the most significant PQSs based on their length and potential stabilization by non-G-tetrads or triads (Table 1). The first group of PQSs in this Table includes the shortest sequences (≤25 nts), which form only unstable hairpins and have an ΔG greater than −1.0 kcal/mol. PQSs including in other groups of Table 1 are the shortest among those stabilized by non-G-tetrads, triads or base pairs (some of them are also included in the first group).

All PQSs presented in Table 1 were analyzed in detail (in the order of their location in the genome) with respect to their structural context, conservation and possible functional roles.

### 3.2. Potential G-Quadruplexes in Gene nsp1 of SARS-CoV-2 gRNA

#### 3.2.1. PQSs 353, 359 and 370

In vivo structural model of the whole SARS-CoV-2 gRNA is presented in the work of Sun et al. [58], and a model of most of the gRNA is presented in the work of Huston et al. [59]. We predicted the secondary structure of SARS-CoV-2 RNA fragments by the UNAFold program based on these two models. The structure of the region encompassing the first 294 nts of the genome is similar in both models and in models reported in other works (for example, Refs. [60,61,62]). The secondary structure of the 5′ terminus of SARS-CoV-2 gRNA, predicted by the UNAFold program for the reference genome Wuhan-Hu-1, is presented in Figure S1. Contrary to models in the literature, it exists in two variants with practically identical ΔG values. The optimal structure contains a large domain with two subdomains, whereas the suboptimal structure is similar to the models in the literature with a single domain. Notably, the reactivity data for the linker nucleotides in the models in the literature do not align well with these models. This may be explained by the coexistence of two nearly equally stable structures, but the models present only one of them.

**Figure 1:**
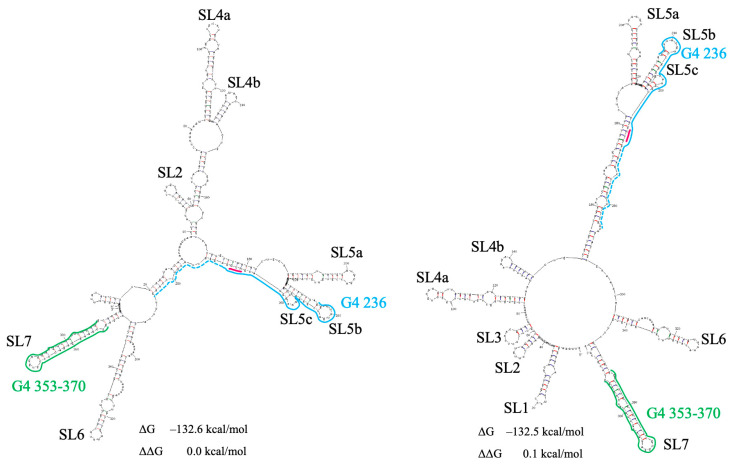
Secondary structure prediction of the SARS-CoV-2 5′ leader with double mutation C44A+C241U. PQSs 236 and 353–370 are indicated by blue and green, respectively. PQS 236 elongation is indicated by a dotted line. The start codon AUG is indicated in red. Hairpin numeration is as in Huston et al. [59]. ΔG—change in free energy. ΔΔG—the energy increment of the lowest change in free energy.

Our large-scale mutational analysis of the SARS-CoV-2 5′ terminal region revealed that three mutations were frequently observed in this region during 2022 to 2024 (Table S3). During this period, the base change C241U was dominant in all five countries, whereas the frequency of base change C21U increased from 0% to 20% in some countries. The double mutation C44A+C241U practically did not alter the structure of the region under study (Figure 1 and Figure S1).

Overlapping PQSs 353, 359 and 370 (Figure S2) are located within stem-loop SL7 of the 5′ leader. These PQSs correspond to the beginning of the first domain of nsp1 protein (Ref. [63] and Figure S3A), implying that their potential roles are unlikely to be co-translational folding of this domain; instead, they may perform other functions. Nsp1 supresses host gene expression through distinct mechanisms [64]; in particular, it disrupts cap-dependent translation by association with the 40S ribosomal subunit, raising the question of how viral mRNAs escape nsp1-mediated repression. Perhaps, G-quadruplexes 353, 359 and 370 play some role in supporting viral transcripts’ translation.

G-quadruplexes located in IRESs (internal ribosomal entry sites) may directly recruit the 40S ribosomal subunit in the vicinity of the AUG start codon ensuring cap-independent translation initiation [65]. IRES can be located both upstream and downstream of AUG. For example, in HIV-1 genomic RNA, one of the IRESs is located within the HIV1 Gag open reading frame and contains G-quadruplex [66]. We suppose that it may participate in translation initiation [67]. By analogy, G4s 353, 359 and 370 may contribute to cap-independent initiation of SARS-CoV-2 translation.

Biophysical assays were conducted only for PQS 353 [27]; they confirmed the formation of G4 by this sequence. At 37 °C, PQSs 353, 353el, 359 and 370 form hairpins with ΔGs of −3.4 kcal/mol, −6.0 kcal/mol, −6.0 kcal/mol and +0.1 kcal/mol, respectively (Table S2). Therefore, G4 formation by PQS 353 and especially by 353el and 359 can compete with a hairpin formation, contrary to PQS 370. Since Razzak et al. [27] used high PQS concentrations in their assays (15 μM), it is possible that multimeric G-quadruplexes could form together with (or instead of) monomolecular G4s in their assays. In addition, hairpins formed by PQSs 353, 353el, 359 and 370 have regions with unpaired GG repeats (Figure S4), which could promote the formation of multimeric structures.

The importance of certain G4s for the SARS-CoV-2 virus may be supported by their strong conservation in other coronaviruses, in particular, in SARS-CoV [22,23]. In SARS-CoV, the hairpin similar to SL7 is also formed (Table S4). It also includes several GG repeats, but only five of them match those in SARS-CoV-2. Thus, there are no direct analogs for PQSs 353, 359 and 370, but there is a similar hairpin encompassing seven redundant GG repeats, which can form different G4s. There are six redundant GG repeats in SARS-CoV-2.

Note that there is one more PQS in the RNA region under study—PQS 236 (Table S2). This sequence has a complete analog in SARS-CoV (Table S4) which may be stabilized by the canonical U·A-U triad. PQSs 236 and 236a form relatively stable hairpins (−8.2 kcal/mol and −9.8 kcal/mol, respectively (Table S2)), and the formation of G4 by PQS 236 without the triad is unlikely. The elongated variant (Table S2) forms a more stable hairpin (−15.5 kcal/mol), but this G4 may be significantly stabilzed by the 3′ U-tetrad. In addition, an internal hairpin (Table S2) can form in its third loop and stabilize it. Stabilization by the 3′ U-tetrad is also possible in the case of SARS-CoV.

PQSs 236, 236a and 236el start at the upper part of SL5b. Whether G-quadruplex stabilized by the 3′ U-tetrad is formed in the domain with SL5a-SL5c and whether it is functionally important is unknown. The repeat UUYYGU hexaloop motif in SL5a and SL5b was proposed to be important for packaging, but its full role in packaging and in other viral functions remains unclear [68]. The dominant mutation C241U did not destroy this motif.

Thus, overlapping G4s 353, 359 and 370 in the SARS-CoV-2 gRNA 5′ terminal region may support cap-independent translation initiation. PQS 370 stands out as a promising drug target: it is short, forms an unstable hairpin and is not affected by dominant 5′ UTR mutations.

#### 3.2.2. PQSs 509, 644 and 653

**Figure 2:**
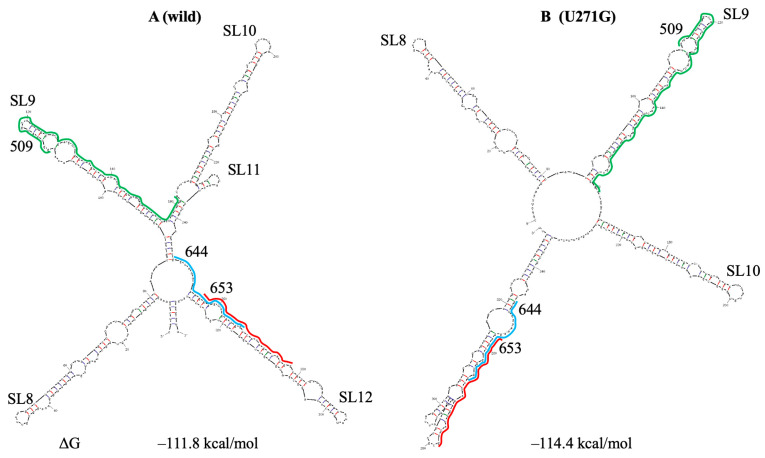
Secondary structure prediction of the SARS-CoV-2 region 5’, containing PQSs 509, 644 and 653. PQSs are underlined by lines. ΔG—change in free energy. (**A**) Without mutations. (**B**) With base change U271G.

Further, for brevity, we will refer to the models of SARS-CoV-2 genomic RNA presented in works of Sun et al. [58] and Huston et al. [59] as Sun and Huston models, respectively. In the Sun model, PQSs 509, 644 and 653 are in a large domain with five hairpins, which is located immediately downstream of SL7. Manfredonia et al. [60] designated the hairpin located immediately downstream of SL7 as SL8. We continued numbering the hairpins further. In the Huston model, hairpins similar to those in the Sun model are separate structural elements. UNAFold program predicts the structure of the region with PQSs 509, 644 and 653 as in the Sun model, only in the suboptimal folding with ΔΔG = 0.5 kcal/mol, while in the optimal folding, three out of five hairpins are localized in a subdomain (Figure 2A).

The dominant mutation U271G in the domain containing PQSs 509, 644 and 653 (Table S5) altered its structure (Figure 2B) and introduced a new GG repeat in PQS 653, generating a shortened PQS 653m (Table S2). PQSs 644, 653, and new PQSs 653m and 659m (Table S2) are short and form unstable hairpins; two of them (PQSs 644 and 653m) we included in Table 1 with the most significant PQSs. PQSs 644 and 653 have analogs in SARS-CoV (Table S4).

In the context of the nsp1 protein, PQS 644 lies at the beginning of the linker between the N- and C-terminal domains and might contribute to correct folding of the large N-terminal domain (Figure S3A). PQS 509, not reported in the literature, may form G4 with a 3′ U-tetrad and forms a moderately stable hairpin (ΔΔG = −6.2 kcal/mol). The potential formation of an internal hairpin in its third loop (Table S2) may further stabilize the G4. PQS 509 does not have a direct analog in SARS-CoV, but a putative G4 with 3′ U-tetrad is in the same region (Table S4); however, it forms the stable hairpin with ΔΔG = −13.1 kcal/mol. Although the direct function of G4 509 remains unclear, highly stable local structures such as 3′ U-tetrad-capped G4s may help maintain the overall architecture of very long RNAs without substantially increasing GC content.

### 3.3. PQSs in Gene nsp2 (1463, 1558, 1574, and 1805)

PQSs 1463, 1558, 1574 and 1805 are located in gene nsp2. PQS 1804/1805a is included in Table 1 as stabilized by a tetrad and base pairs at both sides. PQS 1558/1559 may also be simultaneously capped at both sides of G4 by non-G-tetrads (Table S2). All G4s correspond to the central part of nsp2 (Figure S3B). They may ensure proper protein folding, and their redundancy in this area may be necessary to delay ribosome movement during the synthesis of a rather large protein.

PQSs 1463, 1784 and 1805 have SARS-CoV analogs, whereas PQSs 1559 and 1574 do not (Table S4), although the same genomic region is enriched in PQSs in both viruses.

**Figure 3:**
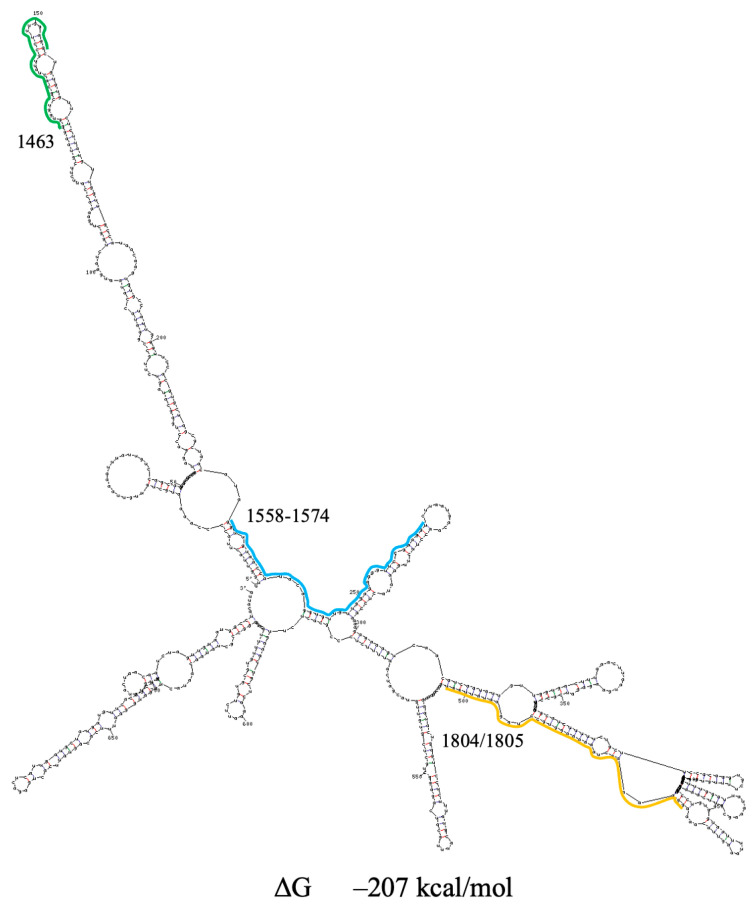
Secondary structure prediction of the SARS-CoV-2 region, containing PQSs 1463, 1558–1574 and 1804/1805. Based on the Sun model. PQSs are underlined by green, blue and yellow, respectively. ΔG—change in free energy.

The secondary structures of the region encompassing PQSs under study are different in the Sun and Huston models; however, PQSs 1559, 1574 and 1805 are located in similar hairpins. We predicted the secondary structure of the region under study based on both models in the literature but additionally included several separated hairpins downstream of the region in the Sun model and upstream of that in the Huston model. UNAFold predicts alternative folds for this region based on the Sun and Huston models, but in all cases, PQS 1805 resides in a conserved subdomain with three hairpins (Figure 3 and Figure S5).

PQS 1463 is among five PQSs which were reported to be the most prone to mutations [27]. It showed a relatively small mutation rate (4%). We did not find mutations with the frequency ≥2% in 2022/2023 directly in this PQS (Table S6). Rare mutation C18A (≤4%) only altered the structure of the middle hairpin in the upper part of the subdomain containing PQS 1805.

At the C-terminus of nsp2, PQSs 2714/2717 lie at the boundary to nsp3 protein (Figure S3B) and may assist in final nsp2 chain folding. PQS 2717 may be stabilized by a 3′ U-tetrad and an internal hairpin (Table S2). PQS 2714 has an analog in the SARS-CoV genome (Table S4), while PQS 2717 has a similar sequence but without all four U bases, which are necessary for the formation of a U-tetrad. UNAFold program predicts structures for the region containing these PQSs, similar to the models in the literature (Figure S6).

Thus, PQSs in gene nsp2 are conserved, and four of them have analogs in the SARS-CoV genome. The probability of forming G4s quadruplexes by some of them is relatively high due to their stabilization by triads or tetrads. They may be necessary for proper nsp2 folding.

### 3.4. PQSs in Gene nsp3

#### 3.4.1. PQS 3467

**Figure 4:**
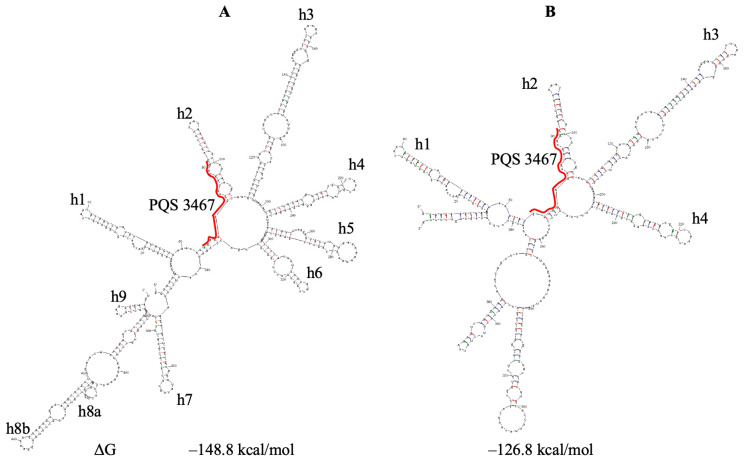
Secondary structure prediction of the SARS-CoV-2 region, containing PQS 3467. (**A**) based on the Sun model. (**B**) based on the Huston model. PQS 3467 is underlined. ΔG—change in free energy; h—hairpin.

PQS 3467 forms the most stable G-quadruplex among SARS-CoV-2 G4s (T_m_ = 63.2 °C [18]). It is located in gene nsp3. We predicted the secondary structure of the region under study directly from the Huston model (Figure 4), whereas, upon folding this region according to the Sun model, we added three small separate structural elements immediately downstream. In both cases, PQS 3467 initiates in short duplexes that close large domains. The formation of G4 by this PQS requires the destruction of not only the second hairpin but also the whole domain, suggesting that this G4 may form only transiently or under specific conditions.

The mutation search (Table S7) showed that unlike many other PQSs, even in 2023, most genomes had practically no mutations in the region under study; they were detected with a relatively high frequency (26%) only in UK (double mutation G62U+U196C). This mutation became dominant (68–82%) in 2024 in all countries for which the mutation search was conducted. All detected mutations, both dominant and rare, did not affect either the PQS 3467 sequence or its location. Since PQS 3467 is located in the gene nsp3 region corresponding to the beginning of Mac1 domain (Figure S3C), presumably, it will not be used for proper folding of the nascent polypeptide chain. In addition, it has no analog in the SARS-CoV genome. However, its high stability and strong conservation suggest an important but as yet unclear function. This PQS is included in Table 1.

#### 3.4.2. PQSs in SARS-CoV-2 gRNA Region Corresponding to SUD Domain (4256/4262,4485/4487 and 4616)

Subdomain SUD (SARS unique domain) of the nsp3 protein attracts special attention because it may be responsible for increased pathogenicity of SARS-type coronaviruses, in particular, SARS-CoV-2 [69]. SUD consists of three subunits: SUD-N, SUD-M and SUD-C. Four GRSs correspond to SUD-N and SUD-M (SUD-core) (Figure S3C). The secondary structure of SARS-CoV-2 gRNA containing PQSs 4256/4262 and 4485/4487 (predicted by the UNAFold program based on the models in the literature) is shown in Figure S7.

A dominant mutation (G380A, later designated G106A in extended searches) emerged in 2022 and rapidly reached frequencies >90%, abolishing G4s 4127/4143/4161 (Table S8). This base change and other most frequent base changes only altered the location of PQSs 4256/4262. Overlapping PQSs 4127, 4143 and 4161 have no analogs in SARS-CoV gRNA, while PQS 4256 has (Table S4). There are sequences similar to PQSs 4487 and 4616 in SARS-CoV gRNA; however, one of four GG repeats is absent in each of these sequences.

SUD-core domains in the nsp3 proteins of SARS-CoV and SARS-CoV-2 can bind DNA and RNA G-quadruplexes [70,71,72,73]. Lavigne et al. [71] showed by HTRF assay (homogenous time-resolved fluorescence) that contrary to cellular G4 TRF2 with three G-tetrads, the most stable viral G4s (13385, 24268 and 28903) do not interact with SUD-NM of SARS-CoV-2. In fact, the affinity of SUD-NM for G4 13385 was approximately five times less than for G4 TRF2, and even less for G4s 24268 and 28903 (Figure 5 in [71]). The absence of SUD-core binding to PQS 28903 was also confirmed by fluorescence assay in the work of Zhang et al. [73]. However, Qin B. et al. [72] showed by EMSA (electrophoretic mobility shift assay) that both G4 TRF2 and G4 24268 bind to SARS-CoV-2 SUD-core.

The formation of G-quadruplex by PQS 24268 has been confirmed in some studies but not in others (Table S1). The absence of G4 was primarily based on the results of the fluorescence assay. However, the low fluorescence intensity could be due to the structural features of this G4 and may not indicate its absence. The HTRF assay in [71] is also based on fluorescence data, and the authors’ results for PQS 24268 may also be due to its specific structure. Thus, viral G4s with two G-tetrads can interact with SUD-core but with significantly less efficiency than those with three tetrads, which are absent in SARS-CoV-2 gRNA but are present in cellular RNAs.

Kusov et al. [74] demonstrated that the SUD-M subunit is indispensable for SARS-CoV replication and suggested that it might be connected with the SUD’s ability to bind G-quadruplexes or oligo(G) tracts. If this is true, then which specific G4s in SARS-CoV-2 might be bound to SUD during replication/transcription? These processes are carried out inside of double-membrane vesicles (DMVs) (for example, Refs. [75,76]). Nsp3, along with other proteins, forms the DMV pore complex. According to Chen et al. [77], nsp3 in the pore complex does not come into contact with the gRNA on which minus-strand synthesis occurs; i.e., there is no interaction between SUD in nsp3 and G4 in gRNA during replication.

However, other nsp3 molecules could be involved in this interaction. Chen et al. [77] do not exclude the possibility that all macromolecules necessary for replication could have been enclosed in DMV at the time of its formation. Nsp3 molecules could have been enclosed in the same way. Weak (transient) SUD binding to RNA G4s and weak nsp3 interaction with most of the other proteins of the replication and transcription complex (RTC) may facilitate reorganization of RTC for participating in different steps of the replication-transcription process, such as minus/plus gRNA and sgRNA synthesis, proof-reading, capping and polyadenylation. Furthermore, SUD may stabilize G-quadruplexes [73]. RTC stalling before stabilized G4s may also facilitate RTC reorganization. Quite recently, Yang et al. [78] showed that RTC localizes in the exterior of DMVs, and nsp12 and other RTC components could be recruited to DMV through direct interaction of nsp12 with nsp3. In this model, gRNA in complex with RTC must enter DMV through the pore complex. In this case, an additional nsp3 protein(s) may also enter DMV either during its formation as free molecules or in complex with gRNA G4s.

The main role in facilitating the RTC reorganization may belong to G4s located at the ends of plus and minus gRNAs where they could regulate initiation or termination events. The first G4 in nascent minus gRNA is located at position 165 of minus gRNA with a potential U-A-U triad in the second loop (Table S2B). A putative G4 that may form when RTC reaches the beginning of plus gRNA is PQS 29867 also stabilized by a U·A-U triad. This G4 may be necessary for initiating the synthesis of the plus-strand RNA and its processing.

In addition to participating in replication (at least in the formation of pore complexes), SUD protein in nsp3 may stimulate translation by binding to 40S/80S ribosome and increasing the interaction between PABP (poly(A)-binding protein) and Paip1 (PABP)-interacting protein 1) [79]. It is unknown whether SUD interacts with G4 during translation initiation, particularly during cap-independent processes. According to Lemak et al. [80], SUD binds to the region 301–545 base including PQSs 353, 359 and 370.

### 3.5. PQSs in nsp4 and nsp5 (8687, 10058/10085, 10254/10260, 10466, 10548/10573 and 10674)

Nsp4, together with nsp3, shapes DMVs (Section 3.4.2). The single PQS (PQS 8687) in gene nsp4 corresponds to the Ecto domain in this protein (Figure S3D), which is located between transmembrane domains 1 and 2. PQS 8687 has an analogous sequence in the SARS-CoV genome, but this sequence does not contain one of the GG repeats to form G4 (Table S4). SARS-CoV gRNA contains other PQSs, corresponding to the Ecto domain. We included PQS 8687 in Table 1, since it is short (23 nts) and forms an unstable hairpin (with ΔG = −0.6 kcal/mol). However, it is unclear in which structural element of gRNA this PQS is located, since the models in the literature for it are too different. The structure based on the Huston model is presented in Figure S8. PQS 8687 is located in it in a small hairpin.

PQS 8687 is located within a very conserved RNA region (Table S9). In 2024, more than 90% of SARS-CoV-2 genomes studied had no base changes in this region. A frequent base change, C169U, occurred in China in 2022 and 2023 but was not found in 2024.

Four GRSs are located in the SARS-CoV-2 gRNA region corresponding to the nsp5 protein; three of them contain overlapping PQSs (Figure S3E, Table S2). Nsp5 is the main protease for processing of the replicase polyprotein [81]. It consists of three domains. PQSs 10058 and 10085, which correspond to the very beginning of domain 1, and PQS 10674, corresponding to the beginning of domain 3, may serve for proper folding of nsp4 and nsp5, respectively.

PQSs 10058, 10085, 10254 and 10260 (like many other PQSs in SARS-CoV-2 gRNA) are located in similar minimal structural elements in the models in the literature, but the structures of gRNA regions containing these elements are different. The secondary structures of the region containing these PQSs based on the Sun and Huston models are shown in Figure S9. PQSs 10254/10260 are either mostly located in similar hairpins, or they are located in duplexes and linkers. A stable dominant double mutation, C19U+C188U (with the frequency of 88–97% in 2024), was found in the studied region (Table S10). It did not affect the structure and location of PQSs in the foldings based on both the models in the literature.

**Figure 5:**
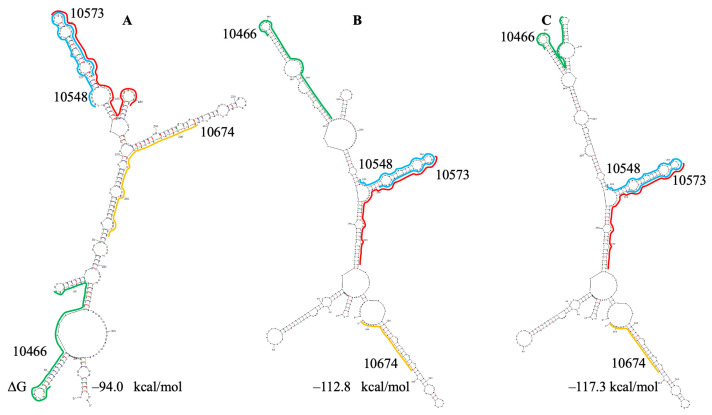
Secondary structure prediction of the of the SARS-CoV-2 region, containing PQSs 10466, 10548/10573 and 10674. (**A**) Based on the Sun model. (**B**,**C**) Based on the Huston model. (**C**) with double mutation. ΔG—change in free energy. ΔΔG—the energy increment of the lowest change in free energy.

The secondary structures of the region containing PQSs 10466 and 10674 based on the Sun and Huston models are shown in Figure 5. PQSs 10466 and 10674 start at the same hairpins in both of the models in the literature and in UNAFold predictions, while the localization of PQS 10573 is different in the models and predictions.

The stable double mutation G102A+G104A with a frequency of 89–96% was found in the region of the RNA genome containing PQSs 10466–10674 (Table S11). It did not impact the structure of the domain based on the Sun model but altered the structure of the hairpin containing PQS 10466 in folding based on the Huston model (Figure 5C). Similar to PQS 28903, it is now located in two hairpins instead of a single one. Several PQSs (10085, 10466, 10548, 10562, 10674) have SARS-CoV analogs, while others lack a complete set of GG repeats (Table S4). Although an analog of PQS 10466 is not stabilized by the potential 3′ U-tetrad, it may also be significantly stabilized (by U·A-U triads at both sides of G4).

Triads or tetrads may stabilize the most putative G4s in gene nsp5. In addition, in some long PQSs, triplexes may be formed if any three GG repeats are located close to each other, for example, in PQS 10674. Three PQSs in gene nsp5 may be significantly stabilized by the 3′ U-tetrad. Two of them (10085 and 10466) we included in Table 1. Gene nsp5, along with gene N, harbous the highest number of PQS variants and the highest density of GRSs containing PQSs (Table S12), suggesting that G4-mediated control may be particularly important for the expression of these genes.

### 3.6. PQS in Gene nsp10 (13385)

PQS 13385 is located in SARS-CoV-2 gene nsp10. This gene contains an important regulatory element, FSE (frameshift stimulation element), which controls the programmed ribosomal frameshifting (Ref. [82] and refs therein). Since PQS 13385 is located 77 nts upstream of the FSE “slippery” sequence, it can impact the structure of the RNA region encompassing FSE. The secondary structures of this region differ across the models in the literature. The Sun model contains a small subdomain and a hairpin within a large domain, which are competent for forming the canonical pseudoknot. In contrast, the Huston model presents two small, separate hairpins, which are competent for forming an alternative pseudoknot. UNAFold program predicts a structure similar to the Sun model (Figure S10) only in the suboptimal folding with a very high value of ΔΔG (7.3 kcal/mol).

**Figure 6:**
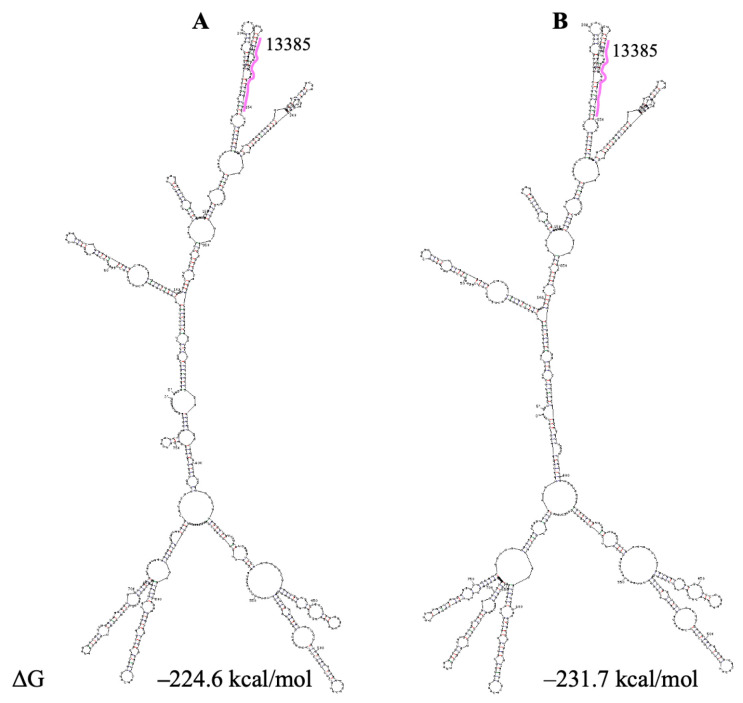
Secondary structure prediction of the SARS-CoV-2 region, containing PQS 13385. (**A**) based on the Sun model. (**B**)—based on the Huston model. G4 is indicted by pink. ΔG—change in free energy.

**Figure 7:**
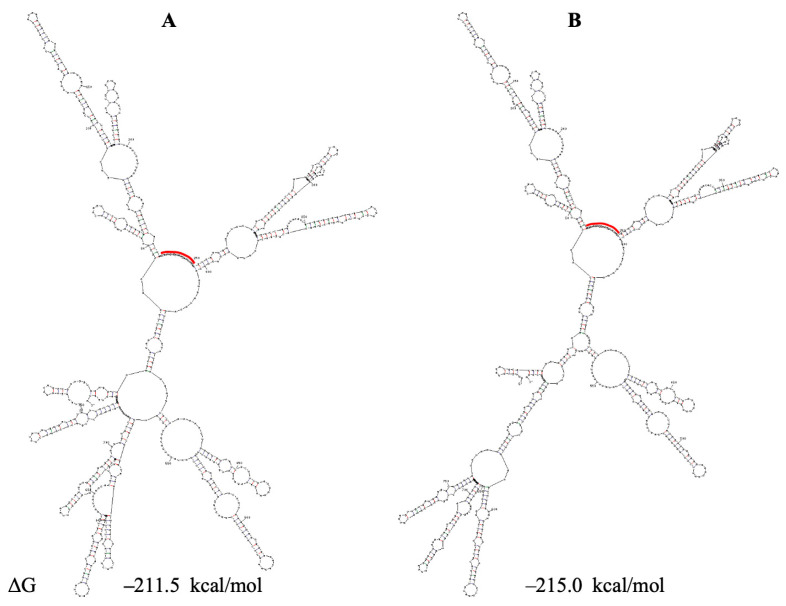
Secondary structure prediction of the SARS-CoV-2 region, containing PQS 13385 with imitation of G4 formation. (**A**) based on the Sun model. (**B**) based on the Huston model. G4 is indicated by red. ΔG—change in free energy.

In the Huston model, the presence of a large linker region between two small hairpins forming the alternative pseudoknot does not coincide with the authors’ experimental data. Therefore, we predicted, by the UNAFold program, the secondary structure of the regions encompassing two large domains in each model in the literature containing PQS 13385 and putative pseudoknots (Figure 6). The upper parts of the structures presented in this figure are identical and do not contain the hairpin involved in the formation of the classic pseudoknot. However, this hairpin is presented in the optimal foldings with the imitation of G4 13385 formation (Figure 7).

Maybe, the identical upper parts in the folding shown in Figure 6 and Figure 7 present the structures of the domain competent for the formation of the classical pseudoknot. This domain is not presented in the models in the literature.

The structure of a minimal RNA fragment involved in the formation of a classical pseudoknot in the SARS-CoV-2 genome is shown in Figure S11A. It consists of a small domain and a hairpin. In Figure S11B,C, the structures of the corresponding fragment in SARS-CoV are also shown. These structures in the genomes of two coronaviruses are similar; in SARS-CoV, the slippery sequence and the 3′ arm of the stem 1 are more exposed.

PQS 13385 has an almost identical analog in the SARS-CoV genome (Table S4). In 2022 and 2023, mutations in the SARS-CoV-2 gRNA region containing PQS 13385 were absent in most genomes (in 80–90%, Table S13), except base change U188C, which occurred with a frequency 46% in the UK in 2023. In 2024 and 2025, this mutation became dominant (70–90%). It practically does not alter the structure of the subdomain participating in the formation of the pseudoknot.

Thus, PQS 13385 is conserved and has an analog in SARS-CoV. SARS-CoV-2 and SARS-CoV gRNAs both contain regions that form the classical pseudoknot. PQS 13385 is short, does not compete with the hairpin formation and may be additionally stabilized by the classical U·A-U triad at each side. We therefore classify it as one of the most functionally significant PQSs in the genome.

### 3.7. PQSs in Gene S

#### 3.7.1. PQS 22316

**Figure 8:**
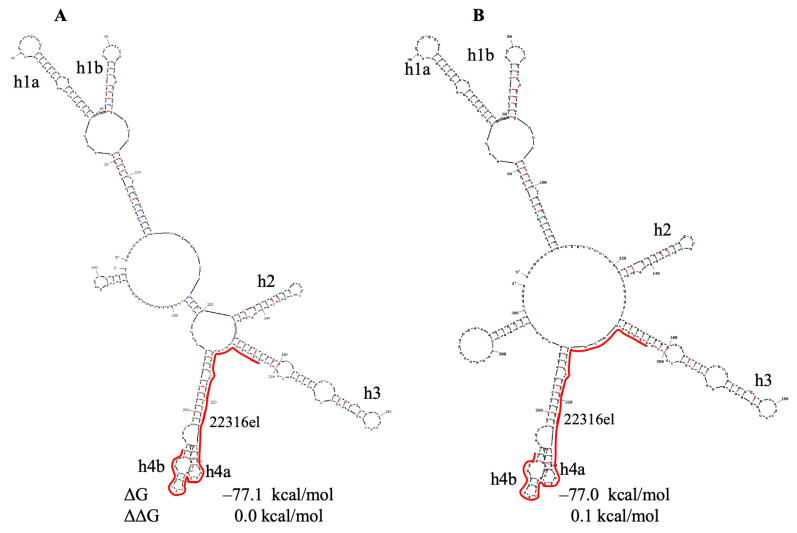
Secondary structure prediction of the of the SARS-CoV-2 region, containing PQS 22316el. ΔG—change in free energy. ΔΔG—the energy increment of the lowest change in free energy. PQS is indicated by line.

PQS 22316 is located in gene S. In spike protein structure [83], it corresponds to the end of the largest domain of this protein (N-terminal domain, NTD, Figure S3F). Therefore, G4 22316 formation may serve for the proper folding of a nascent polypeptide chain. PQS 22316 has no analog in the SARS-CoV genome. In the Sun model, it is located in a hairpin and a small domain. The structure of the SARS-CoV-2 gRNA region containing this PQS (based on the Sun model) is shown in Figure 8.

According to Razzak et al. [27], base changes in the G4 22316 sequence occur rarely (mutation rate < 2%). Our mutation search (Table S14A) shows that early in the pandemic, PQS 22316 was mainly conserved, but in 2022–2023, two G-to-non-G mutations (G68A and G54U) temporarily reached high frequency and disrupted G4s 22316/22316el. On the other hand, a new base change, A57G, appeared in 2022 (Table S14A), and its frequency increased up to 19% in 2023. This mutation results in the formation of a new GG repeat in PQS 22316 and the formation of shorter G4 (21 nts), which does not form a hairpin (ΔG = +0.4 kcal/mol). Additional analysis of 2024–2025 genomes (Table S14B) showed a disappearance of G4-destroying or G4-stabilizing mutations in this region, indicating adverse selection against long-term disruption of G4 22316. Gene S is highly mutable. In 2025, five to seven frequent mutations (with the frequency of 16–46%) were found in the gRNA region shown in Figure 8. Even seven mutations did not affect the structure of the region encompassing hairpins 2, 3 and the subdomain with hairpins 4a/4b, except of small alterations in the upper part of this subdomain (Figure S12).

PQS 22316 contains one of eight pseudouridines (Ψs), found in the SARS-CoV-2 genome [84]. Frequent mutations did not affect its location. Other Ψs in this genome are not located in PQSs, but some of them are present nearby. Pseudouridylation is an abundant RNA modification which can affect its structure and function, in particular, translation (for example, Ref. [85]).

PQS 22316 has been added to Table 1, since it forms a low-stability hairpin, and G4 formed by this sequence may be stabilized by U·A-U triads at each side. In addition, it is a single PQS which is co-located with Ψ in the SARS-CoV-2 genome.

#### 3.7.2. PQSs 24200/24215, 24267/24268 and 25197

**Figure 9:**
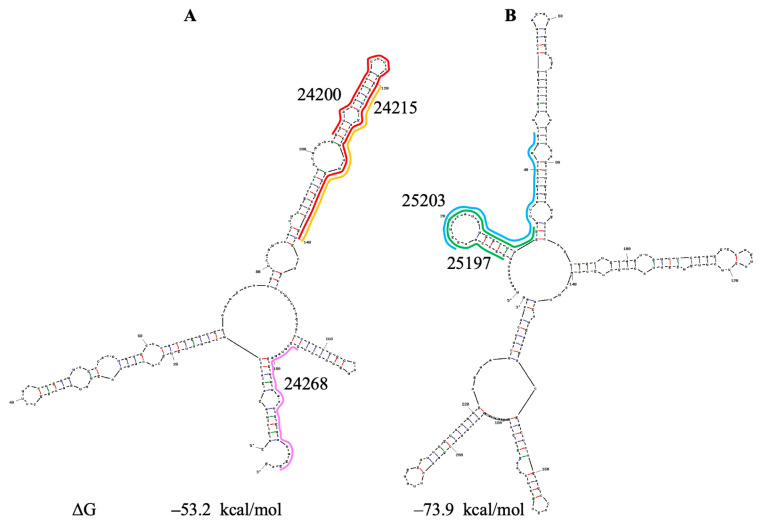
Secondary structure prediction of the SARS-CoV-2 gRNA fragments containing PQSs. (**A**) PQSs 24200/24215 and 24267/24268. (**B**) PQSs 25197/25203. ΔG—change in free energy.

PQS 24267/24268 is included in Table 1, since it is short, forms an unstable hairpin and may be stabilized by a U·A-U triad. The UNAFold program predicts a structure of the region containing PQSs 24267/24268 (and also PQSs 24200/24215) that is similar to the Sun model (Figure 9A). These PQSs correspond (Figure S3F) to the linker between S protein domains FP (fusion peptide) and HR1 (heptapeptide repeat sequence 1) and may serve to fold the preceding domains properly. Frequent base changes occurred in the domain containing PQSs under study in 2021 and 2022 (Table S15). However, in 2023, there were no mutations. There are analogs for all PQSs in the SARS-CoV genome (Table S4); however, the analog of PQS 24200 is unable to form a G-quadruplex stabilized by a 3′ U-tetrad. Conservation of the PQSs 24200, 24215, and 24268, and the availability of analogs, support our proposal regarding their role in the translation process.

At the end of the gene S, there are overlapping PQSs 25197 and 25203. The UNAFold program predicts a structure for the region containing these PQSs that is similar to the Sun model (Figure 9B). Both PQSs are located in two hairpins. A single dominant base change (C21U, about 90%, Table S16) is located in the apical loop of the first hairpin. It does not affect either the region structure or the stability of the hairpins formed by PQSs 25197 and 25203. PQS 25197 corresponds to the boundary between domains HR2 and TM (transmembrane) in S protein (Figure S3F). These PQSs have analogs in SARS-CoV (Table S4); however, they cannot form quadruplexes with a U·A-U triad. So, all PQSs in gene S discussed here may serve to fold S protein domains properly.

Spike protein, along with seven other structural and accessory proteins, is expressed from subgenomic RNAs (sgRNAs) [76]. The sgRNA S is the largest one; it contains the sequence of all SARS-CoV-2 genomes except the ORF1A/ORF1B region. To our knowledge, a structural model of whole sgRNA S is not presented in the literature, although the model of gene S is [86]. The structures of the regions containing different PQSs in gRNA, sgRNA S and gene S will differ (except for the possible presence of identical separated hairpins or small domains).

### 3.8. PQSs in Gene N

Contrary to spike sgRNA, nucleocapsid sgRNA is the smallest one and the most abundant [76]. The gene N model is reported by Zhao et al. [28]. The UNAFold program predicts a similar structure for this region, as shown in Figures S13 and S14. The gene N sequence is shown in Figure S15. The UNAFold program predicts two distinct optimal structures for the second domain; folding in Figure S14A is similar to that in the Zhao et al. model. In addition to the translation region, sgRNA also includes the leader region and the 3′ untranslated region. We conducted a search for mutations practically in the whole gene N (Tables S17–S20) and found two conserved dominant mutations (mainly ˃90%): a 9-nt deletion in PQS 28346 and a triple mutation in PQS 28903 [32].

**Figure 10:**
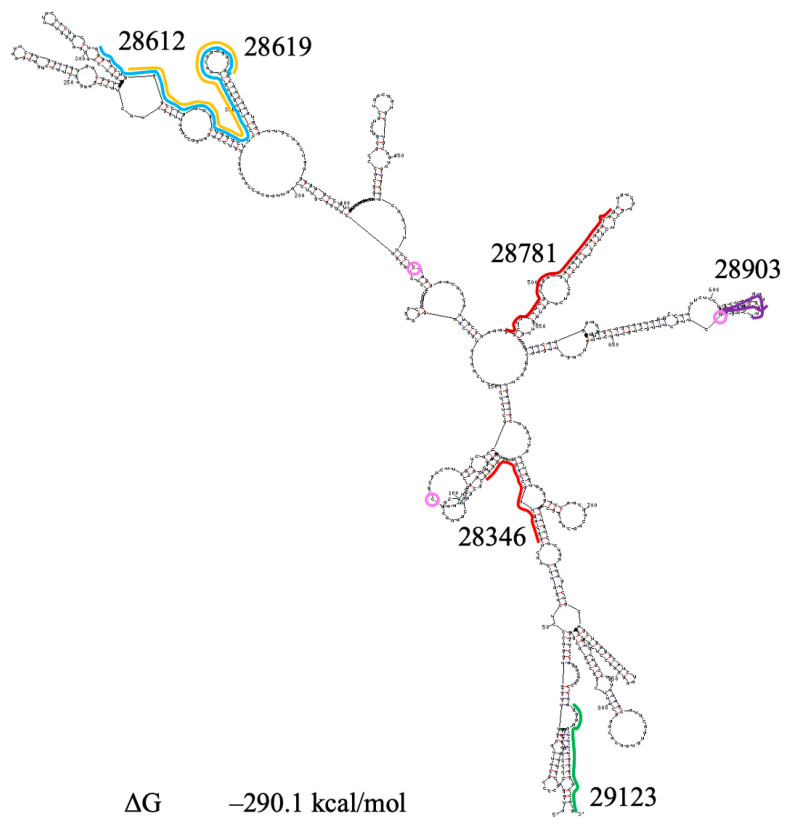
Secondary structure prediction of the domain in SARS-CoV-2 N sgRNA containing PQSs 28346, 28612/28619, 28781, and 28903 and partially 29123. PQSs are indicated by lines; Ψ-sites are indicated by pink circles. ΔG—change in free energy.

The whole sgRNA N has different secondary structures in folding states with close changes in free energy, but all of them contain a large, identical domain containing PQSs 28346, 28613/28619, 28781, and 28903 and partially 29123. The structure of this domain is presented in Figure 10. Two conserved mutations and two frequent mutations identified in 2025 are present in the domain sequence. The domain structure is similar to that of the first domain in gene N. Conserved and frequent mutations affected only the subdomains containing PQS 28903 and PQS 28346.

Gene N includes six GG-rich regions containing overlapping PQSs (Table S2, Figure S2) and four pseudouridine sites (28417, 28759, 28927 and 29418 [84]). Three of them are located in the first domain of gene N, not far from G4s. Contrary to U28759, the locations of U28417 and U28927 are altered in the sgRNA N structure by mutations, which may affect the modification of these uridines. Location of U29418 in a new structure of N gene terminal region was not altered.

The 9-nucleotide deletion in PQS 28346 significantly reduced the length of this PQS and probably increased the stability of G4, which it forms. We only included PQS 29123 in Table 1 as one of the shortest G4 sequences. Furthermore, this PQS can be elongated and stabilized by an A-A-A-A tetrad (Table S2). However, other PQSs in this gene also may form G-quadruplexes, in particular, the shortened PQS 28346 and PQSs with stabilizing triads at both ends of G4 (PQSs 28642 and 29255).

It is unclear which structures a short PQS 28903 forms in the cell—unimolecular G4 (with the help of cellular factors), triplexes [34], dimers [33] or all of them—and what role these structures play in the life cycle of the virus. Considering the great concentration of sgRNA N in a cell, PQSs 28903 can also form tetrameric G-quadruplexes. Moreover, these tetramers may be significantly stabilized by a 3′ U-tetrad, since their bottom part is almost identical to tetramers for which the formation of a highly stabilized structure was reported [56]. One chain of tetramer in PQS 28903 is UGGCUGGCAAUGGCGGU, while that in the work of Andrałojć et al. [56] is UGGUGGU.

All PQSs in gene N have analogous sequences in SARS-CoV; some of them have different GG repeats or have none of the four GG repeats and are not capable of forming a quadruplex (Table S4). Although the structures of nucleocapsid protein are slightly different in different works (for example, Refs. [87,88]), in all of them, PQS 28903 and partially PQS 28781 correspond to the linker region (Figure S3G) and may participate in proper protein N folding.

### 3.9. Study on the Interaction of G4s in SARS-CoV-2 with Ligands

#### 3.9.1. Factors Influencing the Interaction of Quadruplexes with Ligands

**Figure 11:**
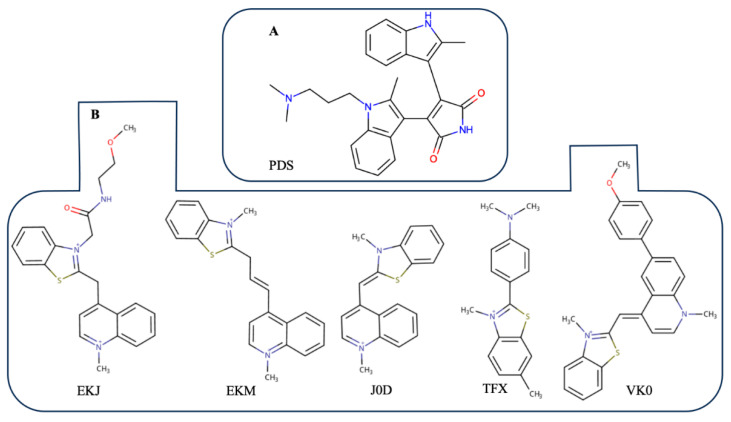
(**A**) PDS. (**B**) Compounds studied by molecular dynamics simulation.

Multiple studies have shown that small compounds targeting G-quadruplexes in SARS-CoV-2 gRNA stabilize certain G4s [18,21,27,28,29,30], which results in inhibition of the viral translation and replication processes [18,27]. In particular, Razzak et al. [27] conducted an extensive study of pyridostatin (PDS, Figure 11A) interaction with G4s in the SARS-CoV-2 genome. They showed that PDS stabilized many out of 25 G4s studied, increasing the melting temperature from 2 °C to 21 °C (in particular, by 14–21 °C for PQSs 644, 3467, 13385 and 29123).

Interestingly, six out of nine G4s which were most significantly stabilized (ΔTm ˃ 8 °C) were unable to form a hairpin (hairpins ΔG were ranged from +1.9 kcal/mol to −0.6 kcal/mol). However, three of these G4s (and each one from the 25 G4s) could be stabilized not intramolecularly but intermolecularly. In addition, all hairpins formed by the studied PQSs have regions with unpaired GG repeats (for example, Figure S4), which could promote the formation of multimeric structures. On the other hand, all nine PQSs forming low-stability hairpins (ΔG ranging from +1.9 kcal/mol to −1.8 kcal/mol) were stabilized by PDS by 4–21 °C, except one with ΔΔ = 0.0 kcal/mol. So, stabilization of G4s by PDSs could depend not only on G4’s structure and the number of chains in it (one, two or four) but also on competence between G4 and a hairpin. Also, flanking nucleotides, which could stabilize G4, were not considered in this work as in most works studying G4 in the SARS-CoV-2 genome. The existence of non-G-tetrads and triads in G4s also could impact the PDS-G4 interaction.

Three G4s in Razzak et al. [27] were destabilized by PDS. These are G4s 1463, 2714 and 25197 destabilized by 1 °C, 7 °C and 3 °C, respectively. PQSs 1463 and 2714 with long loops could not form multimeric G4s, while the formation of a U·A-U triad in G4 25197 might prevent PDS binding. In principle, compounds destabilizing G4 also may serve as therapeutic ligands. Ligands which stabilize G4s, hinder the movement of protein complexes along RNA and impact translation and replication, while destabilizing ligands, could interfere with the performance of important functions of G4s in the viral life cycle. For example, G4 can promote proper folding of nascent polypeptide chain, participate in initiation of translation by binding to 40S ribosome or maintain a certain structure of gRNA and mRNA, in particular, maintain the competent structure in gene nsp 10 for the frameshifting process. In such cases, G4 destabilization can be critical for the survival of the virus.

As a first step, we selected stabilizing ligands for G4s 3467, 13385 and 28903 in this work. For PQSs 3467 and 28903, we previously analyzed structural behavior using molecular dynamics and quantum chemical approaches [31,32]. PQS 3467, as Razzak et al. [27] noted, is located at the 5′ end of ORF1ab, and its stabilization is expected to influence the expression of a number of nonstructural proteins regulating critical processes of the viral life cycle. According to our proposal, G4 13385 may support a competent structure of SARS-CoV-2 gRNA for frameshifting, and destabilizing it could be critical. However, the choice of ligands in this case requires taking into account that U·A-U triads may cap G4 at each side, and this assumption needs to be tested first.

PQS 28903 could serve as an attractive target, as it is located in the gene N region corresponding to a single linker in the protein and may be essential for proper protein folding. However, as mentioned above, it is unclear which structures it forms in the cell. So, as a first step, we searched for stabilizing ligands and only for the monomolecular form of G4.

#### 3.9.2. Ligands Binding to PQS 3467, 13385 and 28903

Taking into account what is written above, molecular docking of 13 compounds into G4s 3467, 13385 and 28903 was performed.

The compounds are presented in Figure 11B and S16. They have the following PDB IDs: A1AEC, A1AED, A1AEE, A1AEF, A1BC9, EKJ, EKM, J0D, POH, R14, TFX, V5Z and VK0. All the compounds were retrieved from the PDB [47]. Four of them (EKM, POH, R14, TFX) are presented in PDB as RNA/DNA G4 binders (predominantly as RNA G4 binders).

In our opinion, there are a few comments about the most common features of docking conformations that are interesting (Figure 13). In the case of interaction with VK0 (Figure 13A), all the aromatic rings are positioned parallel to the plane of the upper guanine tetrad, creating strong π–π stacking interactions with one. As a result, the G-quadruplex is stable. In this binding configuration, the K^+^ ion does not interact with the ligand compound and stays in the central part of the G-quadruplex. The unique feature is that the 3-methyl-1,3-benzothiazol-3-ium fragment partially inserts into the G4 loop. Overall, this binding mode represents a configuration in which the ligand almost completely overlays the planar surface of the G-quadruplex, stabilizing the system predominantly through π–π stacking interactions. Regarding the second binding model shown in Figure 13B, the investigated ligand (EKM) occupies a central position above the tetrad and interacts minimally with it. The 1-methylquinolin-1-ium ligand part is located directly above the K^+^ ion, whereas 3-methyl-1,3-benzothiazol-3-ium (as in the case of VK0) occupies the loop of the G-quadruplex. And at last, for the binding model shown in Figure 13C, the ligand covers the central part of the G-quadruplex above the K^+^ ion. In addition, it forms stacking contacts with one side of the tetrad and partially fills the loop with a charged nitrogen atom. Under these conditions, cation–π interactions are likely to occur, which may further contribute to structural stabilization. Note that this binding mode combines the two previous configurations shown in Figure 13A,B.

The top-ranked molecular docking results (Figure 11B) were selected for a two-stage MD simulation: an initial 100 ns simulation, followed by an extended 500 ns simulation for the most promising complexes (see Materials and Methods for details). Subsequent complexes were investigated: the complex between G4 3467 and EKJ; complexes between G4 13385 and EKJ, VK0, and EKM; and complexes between the gRNA fragment containing G4 28903 and EKM and POH.

**Figure 12:**
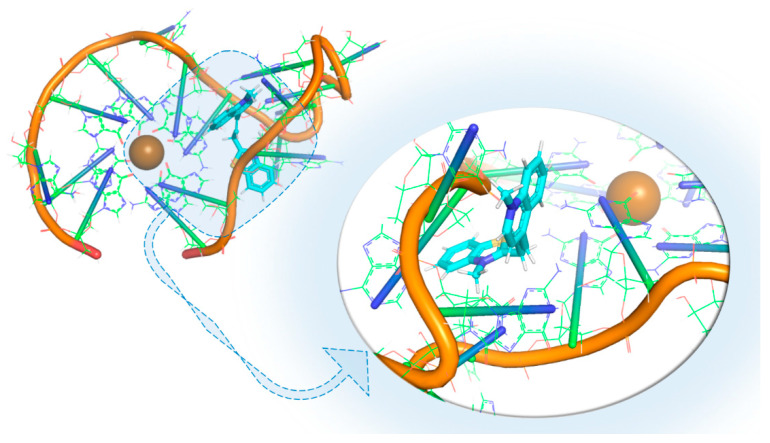
Visualization of the most interesting binding modes of compounds with known activity against G4 3467. All structures are shown as ribbon models, with the RNA strands depicted in orange and the guanine tetrads represented as green planes. The brown sphere in the center of each complex corresponds to a potassium ion (K^+^), which stabilizes the G-quadruplex structure.

MD analysis has shown that the G4 3467-EKM complex remained stable throughout all of the simulation (100, 500 ns). It was found that EKM stabilized in an unusual binding pocket that is located among the G4 3467 loops (Figure 12). We believe that this configuration is a potentially new model target for expanding the search for or creating similar ligands in the future.

**Figure 13:**
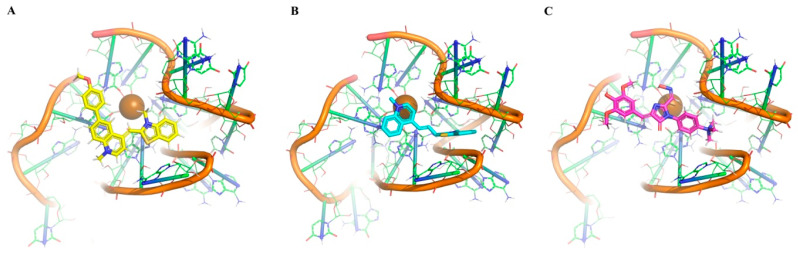
Visualization of the most interesting binding modes of compounds with known activity against G4 13385. All structures are shown as ribbon models, where the RNA strands are depicted in orange and the guanine tetrads are represented as green planes. The brown sphere in the center of each complex corresponds to a potassium ion (K^+^), which stabilizes the G-quadruplex structure. (**A**)—binding with VK0 (**B**)—binding with EKM, (**C**)—binding with V5Z.

In the case of 13385, its interaction with VK0 and EKM results in a stable complex (Figure 13). Furthermore, the interaction mode with those two compounds (creating strong interaction) suggests them as very promising candidates for further investigation and optimization.

Table 2 summarizes the binding free energy (ΔG, kcal/mol) calculated for the investigated ligand–G4 complexes over 100 ns MD simulations. EKM most strongly binds to G4 3467, while EKJ binds to G4 13385 [27] according to MD simulation conducted for the complex of G4 3467 with PDS. This complex is stabilized via π-stacking along with electrostatic interactions between the RNA phosphate backbone and PDS amines. Contrary to EKM, PDS did not interact with G4 3467 loops and, therefore, may be a less selective ligand.

The stabilization of a G4–ligand complex can be realized by contacts with adjacent RNA regions [89]. Indeed, we observed this behavior for the 36-nt gRNA fragment containing G4 28903 in complex with POH. This fragment is the hairpin in the SARS-CoV-2 reference genome (without the triple mutation) containing PQS 28903.

In the pre-MD binding mode, POH is positioned largely parallel to the terminal G-tetrad plane and, in addition to contacts with the G4 core, forms extensive van der Waals interactions with the terminal nucleotides C35 and U36 (Figure S17A, B). Notably, this initial arrangement is dominated by shape/packing complementarity: only one favorable π–π stacking interaction is detected, while strong directional interactions (conventional hydrogen bonds or salt bridges) are absent. During molecular dynamics simulation, the ligand undergoes a pronounced change in its local environment relative to the initial docking pose. The nucleic acid architecture rearranges, and the ligand shifts into a new binding mode that is optimized for the post-MD conformation rather than for the starting structure. In this final state, contacts with the adjacent unpaired segment become a major stabilizing factor and help maintain the ligand–nucleic acid complex over time. Concomitantly, the G4-containing fragment undergoes a major structural transition, with the quadruplex core converting into a G-triplex (Figure S17C,D). Thus, a practical outcome of such remodeling can be the emergence of selective ligand recognition of an RNA region adjacent to a G4. This is precisely the type of specificity that appears to occur in our system.

Finally, we would like to mention the following. The compounds used in this work might bind to both cellular and SARS-CoV-2 viral G-quadruplexes. However, during viral infection, the amount of viral G4s considerably exceeds that of cellular G4s (Ref. [18] and refs therein, Refs. [88,90]). Therefore, we expect that EKM will bind preferentially to viral RNA G4 rather than cellular RNA/DNA G4s.

The SARS-CoV-2 genome contains at least 42 G-rich regions with PQSs, including 28 regions that harbor overlapping PQSs (Table S2). Possibly, the occurrence of redundant GG repeats in the regions with the overlapping G4s ensures the performance of an important function by G4 in these locations. GRSs with PQSs are distributed unevenly in the genome (Table S12), with the largest number of them in genes nsp3 (7 PQSs), N (6 PQSs), S (5 PQSs), nsp2 and nsp5 (4 PQSs each), nsp1 and nsp12 (3 PQSs each).

About 60% PQSs in the SARS-CoV-2 genome may be stabilized by non-G-tetrads, triads or base pairs. Among triads, we considered only the most stable U·A-U triad; however, in some putative G4s, including unstabilized ones, we found an A·A-U triad which also occurs rather frequently in RNAs [53]. Overlapping PQSs and stabilizing elements are not only features of G-quadruplexes in the SARS-CoV-2 genome, but they are also characteristic of G4s in genomes of other viruses, and in each virus, G4s have their own distinctive features. In particular, we did not find putative G4s stabilized by the 3′ U-tetrad in HIV-1 genome, but it contains three regions with 3 tetrad-G4, which are absent in the SARS-CoV-2 genome. Such very stable G4s may serve to maintain a certain structure of RNA. Many putative G4s in the SARS-CoV-2 genome contain U·A-U triads, while those in HIV-1 contain more A·A-U triads than U·A-U ones.

Phylogenetic analysis performed in this work and reported in the literature [27] showed that despite the high mutational rate of the SARS-CoV-2 genome, mutations directly in PQSs (in GG repeats or in loops) occurred rarely (Table S21). Moreover, base changes destroying some G4s (in particular, 22316 and 28903) turned out to be temporary. Contrary to this, in some PQSs (644, 22316, 28346), base changes led to stabilization of G4s. In particular, mutation in the loop of PQS 22316 decreases the stability of the hairpin formed by this PQS by 2.3 kcal/mol, correspondingly increasing the probability of G4 formation. Conservation of G4s in SARS-CoV-2 gRNA and the existence of analogs for many PQSs in the SARS-CoV genome indicates their functional importance.

To our knowledge, the functions of G4s in the SARS-CoV-2 life cycle are not known. Eight Ψ-sites were found in the SARS-CoV-2 genome (in the second half of it); only one of them was co-located with G4, and three Ψ-sites were located near G4s. Their functions are also unknown. Moreover, it is unclear how SARS-CoV-2 G-quadruplexes form in gRNA or sgRNA in the cell. Usually, PQSs are located in hairpins or in several structural elements (hairpins, internal duplexes and linkers) within large RNA domains or subdomains. Due to G4s’ low stability, their formation in RNA domains is disadvantageous, even if there is no competition with hairpin formation.

However, in cells, factors such as crowding agents, peptides, and proteins can promote G4 formation (Mukherjee et al. [17] and refs. therein). In addition, PQSs are mostly partially exposed in the hairpin apical loops, and the hairpins, including them, have internal loops (Figure 1, Figure 2, Figure 3, Figure 4, Figure 5, Figure 6, Figure 8, Figure 9 and Figure 10) that may promote PQS interaction with different cellular factors and proteins.

In this work, we also studied the interactions of G4s 3467, 13385 and 28903 with 13 compounds by molecular docking. For further molecular dynamics simulations, we selected EKM (for G4 3467) and EKJ, EKM, and VK0 (for G4 13385). EKM was found as a promising antiviral agent for the interaction with G4 at position 3467, as it has binding sites both to the quadruplex and to the G4 loop, which contributes to its selectivity. The compounds used in this work might preferentially bind to cellular DNA/RNA rather than to viral RNAs. However, it was proposed that during viral infection, the amount of viral G4s considerably exceed that of cellular G4s (Ref. [18] and refs therein, Refs. [88,90]), and ligands will primarily bind to viral G-quadruplexes.

The main stages of our workflow are summarized in Table 3. This scheme can also be applied to search for significant G-quadruplexes in genomes of other viruses. While quantum chemical calculations were not performed within the present study, we previously applied them to G4s 3467 and 28903 [31,32]. Investigation of G4 structures stabilized by tetrads and triads, docking of ligands on G4s as parts of a minimal gRNA structural elements, and other studies are planned to be carried out in the future.

Experimental studies are now required to elucidate the biological functions of the prioritized G4S and to select the most effective ligands for developing antiviral drugs. Since most PQSs are located in large RNA domains, G-quadruplexes’ formation may be a regulated process. It is important to understand these regulatory mechanisms.

## Figures and Tables

**Table 1:** The most significant PQSs in SARS-CoV-2 gRNA.

PQS	Gene	Sequence	Length, nts	ΔG kcal/mol	Caps
		The Shortest PQSs *			
370	nsp1	u**gg**a**gg**a**gg**ucuuaucaga**gg**c	20	+0.1	-
644		c**gg**uaauaaa**gg**agcu**gg**u**gg**c	20	+1.1	-
653m		a**gg**agcu**gg**u**gg**ccaua**gG**ua	18	−0.7	-
3467	nsp3	au**gg**a**gg**a**gg**uguugca**gg**a	17	+1.1	-
8687	Nsp4	ua**gg**auacaa**gg**cuauugau**gg**u**gg**u	23	−0.6	-
13385	nsp10	c**gg**uaugu**gg**aaa**gg**uuau**gg**c c**ggu**augu**gga**aa**ggu**uau**gg**c c**gg**uatg**ugg**aa**agg**uua**ugg**c	20	+1.9	-
13385a			20	+1.9	3′ uau
13385b			20	+1.9	5′ uau
24267	S	a**ugg**cuuau**agg**uuuaa**ugg**uauu**gg**a au**gg**cuuaua**gg**uuuaau**gg**uauu**gg**a	25	−0.2	5′ uau
24268			24	−0.2	-
29123	N	a**gg**aaauuuu**gggg**acca**gg**a	19	−0.6	-
PQSs Stabilized by Non-G-Tetrads or Base Pairs					
1804	nsp2	a**agg**aaaagcuaaaaa**agg**ugcc**ugg**aauau**ugg**ug a**gga**aaagcuaaaaaa**ggu**gccu**gga**auauu**ggu**g	33	−1.9	5′ aauu
1805a			33	−1.9	3′ auau
PQSs Stabilized by 3′ U-Tetrad					
509	nsp1	u**ggu**cauguuau**ggu**ugagcu**ggu**agcagaacucgaaggcauucaguac**ggu**c	51	−6.2	3′ uuuu
4485	nsp3	ua**agg**guauuaaaatacaag**agg**gug**ugg**uugauua**ugg**u g**ggu**auuaaaauacaagag**ggu**gu**ggu**gauuau**ggu**g	37	−3.0	5′ aauu
4487			35	−3.0	3′ uuuu
10085	nsp5	cu**ggu**aaaguugag**ggu**uguau**ggu**acaaguaacuugu**ggu**a u**ugg**uaaaguug**agg**guugua**ugg**uacaaguaacuugu**gg**u	39	−4.6	3′ uuuu
10084			39	−4.6	5′ uau
10466a		auuaag**ggu**ucauuccuuaau**ggu**ucaugu**ggu**aguguu**ggu**uuuaac a**agg**guucauuccuuaa**ugg**uucaugu**gg**uagugu**ugg**u	36	−7.8	3′ uuuu
10464			37	−5.7	5′ uau
PQSs Stabilized by U·A-U Triad					
4256a	nsp3	g**ggu**cag**gg**uuuaaau**ggu**uacacuguaga**gga**g	32	−1.0	3′ uau
22315	S	c**ugg**ugauucuucuuc**agg**u**ugg**acagcu**gg**u u**gg**ugauucuucuuca**ggu**u**gga**cagcu**ggu**gc	30	−1.8	5′ uau
22316a			30	−1.8	3′ uau

* Dinucleotides and trinucleotides gg, gga, ggu, agg and ugg are indicated by red. Base change in PQS 653 is marked with a capital letter.

**Table 2:** The binding free energy (ΔG, kcal/mol) calculated for ligand–G4 complexes.

**Ligand**	**ΔG kcal/mole**	
	**G4 3467**	**G4 13385**
EKJ	−15.33	−21.03
EKM	−25.22	−14.83
J0D	−13.35	−17.71
TFX	−11.78	−14.37
VK0	−15.02	−9.83

ΔG calculations are based on 100 ns MD at 35 C.

**Table 3:** Searching for significant G-quadruplexes in SARS-CoV-2 gRNA (steps).

	**Primary Structure**
1	Search for GG-rich regions with ≥4 closely located GG repeats.
2	Visual search for putative stabilizing non-G-tetrads and triads in the PQSs found.
3	Search for PQS analogs in SARS-CoV genome.
4	Determination of PQSs’ correspondence to the positions in proteins.
5	Consideration of the literature data on PQSs.
	**Secondary Structure**
6	Secondary structure prediction of the hairpins formed by PQSs.
7	Secondary structure prediction of gRNA regions containing PQSs (based on the literature data).
8	Search for mutations in these regions and prediction of their structure with frequent mutations.
	**Selection of Ligands**
9	Selection of stabilizing or destabilizing ligands based on putative G4 function.
10	Docking of different ligands on G4s directly.
11	Docking of selected ligands on G4s as parts of a minimal gRNA structural elements.
	**Detailed Structure**
12	Molecular dynamics assay.
13	Quantum chemical calculations.

## Data Availability

The original contributions presented in this study are included in the article/supplementary material. Further inquiries can be directed to the corresponding author(s).

## Supplementary Materials

The following supporting information can be downloaded at: https://www.mdpi.com/article/10.3390/v18020253/s1, Figure S1. Secondary structure prediction of the SARS-CoV-2 5′ leader. PQSs 236 and 353–370 are indicated by blue and green lines, respectively. PQS 236 elongation is indicated by a dotted line. Start codon AUG indicated by red. Hairpin numeration is as in [59]. ΔG—change in free energy. ΔΔG—the energy increment of the lowest change in free energy; Figure S2. Overlapping PQSs. GG repeats (together with other bases) in PQS stabilized by tetrads or triads are indicated by pink color, those in unstabilized PQSs are indicated by red color. The mutation in PQS 653m is marked with a capital letter; Figure S3. The domain organization of proteins in SARS-CoV-2 and G4s correspondence to the regions in them. AA—aminoacids. G4—protein regions corresponding to G-quadruplexes. Protein domains are indicated by different colors, while those corresponding to the positions of G4s are indicated by red (see Refs. [63,81,83,87,91,92] for details); Figure S4. Hairpins formed by PQSs 353, 353el, 359, 370 and elongated PQS 353 (as in [22]. GG repeats are indicated by green. Repeats in putative G4 stabilized by triad are indicated by blue. ΔG—change in free energy; Figure S5. Secondary structure prediction of SARS-CoV-2 region containing PQSs 1463, 1558–1574, and 1804/1805. Based on the Huston model. PQSs are underlined by green, blue and yellow, respectively. ΔG—change in free energy. ΔΔG—the energy increment of the lowest change in free energy. PQSs are indicated by lines; Figure S6. Secondary structure prediction of the SARS-CoV-2 5′ region, containing PQSs 2714/2717. (A) based on the Sun model. (B) based on the Huston model. PQSs are underlined, PQS 2717 is underlined by a dotted line. Hairpins are numerated based on the Sun model; h1(2,4)sh—shortened variants of the hairpins. ΔG—change in free energy; Figure S7. Secondary structure prediction of the SARS-CoV-2 region, containing PQSs 4256/4262 and 4485. PQSs are underlined by green and blue, respectively. (A and B)—two different optimal structures with identical ΔG. ΔG—change in free energy; Figure S8. Secondary structure prediction of the SARS-CoV-2 region containing PQS 8687. PQS is underlined. ΔG—change in free energy. ΔΔG—the energy increment of the lowest change in free energy; Figure S9. Secondary structure prediction of the SARS-CoV-2 region, containing PQSs 10058/10085 and 10254/10260. (A,B) Based on the Sun model. (C,D) Based on the Huston model. ΔG—change in free energy. ΔΔG—the energy increment of the lowest change in free energy; Figure S10. Secondary structure prediction of the SARS-CoV-2 region containing PQSs 13385 based on the Sun model. (A) wild. (B) with constraints on the folding of the structure competent for classic pseudoknot formation [93]. The arms of stem one are indicated by red. The arms of stems 2 and 3 are indicated by blue and green, respectively. Slippery sequence (SS) is indicated by a rectangle. G4 is indicated by yellow. ΔG—change in free energy. ΔΔG—the energy increment of the lowest change in free energy; Figure S11. Secondary structure prediction of the minimal RNA fragment containing the sequence of the classical pseudoknot participating in the frameshifting process. (A) SARS-CoV-2, (B and C) SARS-CoV. Slippery site is indicated by yellow; the arms of pseudoknot duplexes are indicated by numbers. Figure is based on Kelly et al.’s model [93]. ΔG—change in free energy. ΔΔG—the energy increment of the lowest change in free energy; Figure S12. Secondary structure prediction of the SARS-CoV-2 region, containing PQS 22316el with 7 mutations. Optimal folding. PQS is indicated by line. ΔG—change in free energy; Figure S13. Secondary structure prediction of SARS-CoV-2 region, containing 1st domain of gene N RNA. ΔG—change in free energy. PQSs are indicated by line; Figure S14. Secondary structure prediction of SARS-CoV-2 region, containing 2nd domain of gene N RNA. PQSs are indicated by line. Ψ-site is indicated by pink circle/ΔG—change in free energy; Figure S15. Gene N, nts 28274–29533. Start and stop codons are indicated by pink. GG(GGG) repeats are indicated by red. Pseudouridine sites are indicated by blue and shown in capital letters. The start of the second domain is indicated by yellow; Figure S16. Chemical formulas of compounds; Figure S17. Spatial structure of the SARS-CoV-2 genomic RNA fragment nts 28903–28927 in complex with POH before (A) and after (C) molecular dynamics and 2D interaction diagram at the corresponding site before (B) and after (D) molecular dynamics; Table S1 Biophysical assays used to validate G4 formation of PQSs in SARS-CoV-2 genomic RNA; Table S2A. PQSs in positive-sense SARS-CoV-2 gRNA; Table S2B. PQSs in negative-sense SARS-CoV-2 gRNA; Table S2C. Putative G-triplex sequences in SARS-CoV-2 genomic RNA; Table S3. Mutational analysis of the SARS-CoV-2 5′ terminal region (%); Table S4. Comparison of PQSs in SARS-CoV and SARS-CoV-2 genomic RNAs; Table S5. Mutational analysis of the SARS-CoV-2 region containing PQSs 509, 644 and 653 (%); Table S6A. Mutational analysis of the SARS-CoV-2 region containing PQSs 1463 and 1558 (%); Table S6B. Mutational analysis of the SARS-CoV-2 region containing PQS 1805 (%); Table S7. Mutational search in SARS-CoV-2 region containing PQS 3467 (%); Table S8A. Mutational search in SARS-CoV-2 gRNA region corresponding to SUD subdomain in nsp3 protein (%); Table S8B. Mutational search in SARS-CoV-2 gRNA region corresponding to SUD subdomain in nsp3 protein (%); Table S9. Mutational search in SARS-CoV-2 gRNA region containing PQS 8687 (%); Table S10. Mutational search in SARS-CoV-2 gRNA region containing PQSs 10058/10085 and 10254/10260 (%); Table S11. Mutational search in SARS-CoV-2 gRNA region containing PQSs 10466–10674, (%); Table S12. GRSs in SARS-CoV-2 genome; Table S13. Mutational search in SARS-CoV-2 gRNA region containing PQS 13385 (%); Table S14A. Mutational search in SARS-CoV-2 gRNA 120 nts region containing PQS 22316 and Ψ-site 22322 (%); Table S14B. Additional search in the elongated region; Table S15. Mutational search in SARS-CoV-2 gRNA region containing PQSs 24200/24215 and 24267/24268 (%); Table S16. Mutational search in SARS-CoV-2 gRNA region containing PQSs 25197/25203 (%); Table S17. Mutational search in SARS-CoV-2 gRNA region containing PQS 28346 (%); Table S18. Mutational search in SARS-CoV-2 gRNA region containing PQSs 28613 and 28781 (%); Table S19. Mutational search in SARS-CoV-2 gRNA region containing PQS 28903 (%); Table S20. Mutational search in SARS-CoV-2 gRNA region containing PQS 29123 and 29234 (%); Table S21. The most frequent mutations in SARS-CoV-2 gRNA regions containing PQSs.

## References

[1] Fay M.M., Lyons S.M., Ivanov P. (2017). RNA G-Quadruplexes in Biology: Principles and Molecular Mechanisms. J. Mol. Biol..

[2] Lavezzo E., Berselli M., Frasson I., Perrone R., Palù G., Brazzale A.R., Richter S.N., Toppo S. (2018). G-quadruplex forming sequences in the genome of all known human viruses: A comprehensive guide. PLoS Comput. Biol..

[3] Kharel P., Becker G., Tsvetkov V., Ivanov P. (2020). Properties and biological impact of RNA G-quadruplexes: From order to turmoil and back. Nucleic Acids Res..

[4] Bohálová N., Cantara A., Bartas M., Kaura P., Šťastný J., Pečinka P., Fojta M., Mergny J.-L., Brázda V. (2021). Analyses of viral genomes for G-quadruplex forming sequences reveal their correlation with the type of infection. Biochimie.

[5] Dumas L., Herviou P., Dassi E., Cammas A., Millevoi S. (2021). G-Quadruplexes in RNA Biology: Recent Advances and Future Directions. Trends Biochem. Sci..

[6] Lyu K., Chow E.Y.-C., Mou X., Chan T.-F., Kwok C.K. (2021). RNA G-quadruplexes (rG4s): Genomics and biological functions. Nucleic Acids Res..

[7] Qi T., Xu Y., Zhou T., Gu W. (2021). The Evolution of G-quadruplex Structure in mRNA Untranslated Region. Evol. Bioinform..

[8] Zareie A.R., Dabral P., Verma S.C. (2024). G-Quadruplexes in the Regulation of Viral Gene Expressions and Their Impacts on Controlling Infection. Pathogens.

[9] Abiri A., Lavigne M., Rezaei M., Nikzad S., Zare P., Mergny J.-L., Rahimi H.-R. (2021). Unlocking G-Quadruplexes as Antiviral Targets. Pharmacol. Rev..

[10] Ruggiero E., Zanin I., Terreri M., Richter S.N. (2021). G-Quadruplex Targeting in the Fight against Viruses: An Update. Int. J. Mol. Sci..

[11] Ruggiero E., Richter S.N. (2023). Targeting G-quadruplexes to achieve antiviral activity. Bioorganic Med. Chem. Lett..

[12] Zhai L.-Y., Su A.-M., Liu J.-F., Zhao J.-J., Xi X.-G., Hou X.-M. (2022). Recent advances in applying G-quadruplex for SARS-CoV-2 targeting and diagnosis: A review. Int. J. Biol. Macromol..

[13] Bezzi G., Piga E.J., Binolfi A., Armas P. (2021). CNBP Binds and Unfolds In Vitro G-Quadruplexes Formed in the SARS-CoV-2 Positive and Negative Genome Strands. Int. J. Mol. Sci..

[14] Zhang A.Y.Q., Balasubramanian S. (2012). The Kinetics and Folding Pathways of Intramolecular G-Quadruplex Nucleic Acids. J. Am. Chem. Soc..

[15] Zarudnaya M.I., Kolomiets I.M., Potyahaylo A.L., Hovorun D.M. (2019). Structural transitions in poly(A), poly(C), poly(U), and poly(G) and their possible biological roles. J. Biomol. Struct. Dyn..

[16] Faure G., Ogurtsov A.Y., Shabalina S.A., Koonin E.V. (2017). Adaptation of mRNA structure to control protein folding. RNA Biol..

[17] Mukherjee S.K., Knop J.-M., Winter R. (2022). Modulation of the Conformational Space of SARS-CoV-2 RNA Quadruplex RG-1 by Cellular Components and the Amyloidogenic Peptides α-Synuclein and hIAPP. Chem. A Eur. J..

[18] Qin G., Zhao C., Liu Y., Zhang C., Yang G., Yang J., Wang Z., Wang C., Tu C., Guo Z. (2022). RNA G-quadruplex formed in SARS-CoV-2 used for COVID-19 treatment in animal models. Cell Discov..

[19] Basu P., Kejnovská I., Gajarský M., Šubert D., Mikešová T., Renčiuk D., Trantírek L., Mergny J.-L., Vorlíčková M. (2024). RNA G-quadruplex formation in biologically important transcribed regions: Can two-tetrad intramolecular RNA quadruplexes be formed?. Nucleic Acids Res..

[20] Belmonte-Reche E., Serrano-Chacón I., Gonzalez C., Gallo J., Bañobre-López M. (2021). Potential G-quadruplexes and i-Motifs in the SARS-CoV-2. PLoS ONE.

[21] Cervenak M., Molnár O.R., Horváth P., Smeller L. (2024). Stabilization of G-Quadruplex Structures of the SARS-CoV-2 Genome by TMPyP4, BRACO19, and PhenDC3. Int. J. Mol. Sci..

[22] Cui H., Zhang L. (2020). G-Quadruplexes Are Present in Human Coronaviruses Including SARS-CoV-2. Front. Microbiol..

[23] Ji D., Juhas M., Tsang C.M., Kwok C.K., Li Y., Zhang Y. (2021). Discovery of G-quadruplex-forming sequences in SARS-CoV-2. Brief. Bioinform..

[24] Kabbara A., Vialet B., Marquevielle J., Bonnafous P., Mackereth C.D., Amrane S. (2022). RNA G-quadruplex forming regions from SARS-2, SARS-1 and MERS coronoviruses. Front. Chem..

[25] Liu G., Du W., Sang X., Tong Q., Wang Y., Chen G., Yuan Y. (2022). RNA G-quadruplex in TMPRSS2 reduces SARS-CoV-2 infection. Nat. Commun..

[26] Paul R., Dutta D., Bhattacharyya T., Paul R., Pradhan S., Dash J. (2025). SARS-CoV-2 RNA G-quadruplex-templated synthesis of a small molecule to regulate Nsp10 expression. Cell Rep. Phys. Sci..

[27] Razzaq M., Han J.H., Ravichandran S., Kim J., Bae J.-Y., Park M.-S., Kannappan S., Chung W.-C., Ahn J.-H., Song M.J. (2023). Stabilization of RNA G-quadruplexes in the SARS-CoV-2 genome inhibits viral infection via translational suppression. Arch. Pharmacal Res..

[28] Zhao C., Qin G., Niu J., Wang Z., Wang C., Ren J., Qu X. (2021). Targeting RNA G-Quadruplex in SARS-CoV-2: A Promising Therapeutic Target for COVID-19?. Angew. Chem. Int. Ed..

[29] Miclot T., Hognon C., Bignon E., Terenzi A., Marazzi M., Barone G., Monari A. (2021). Structure and Dynamics of RNA Guanine Quadruplexes in SARS-CoV-2 Genome. Original Strategies against Emerging Viruses. J. Phys. Chem. Lett..

[30] D’Anna L., Miclot T., Bignon E., Perricone U., Barone G., Monari A., Terenzi A. (2023). Resolving a guanine-quadruplex structure in the SARS-CoV-2 genome through circular dichroism and multiscale molecular modeling. Chem. Sci..

[31] Gorb L., Voiteshenko I., Hurmach V., Zarudnaya M., Nyporko A., Shyryna T., Platonov M., Roszak S., Rasulev B. (2024). From RNA sequence to its three-dimensional structure: Geometrical structure, stability and dynamics of selected fragments of SARS-CoV-2 RNA. NAR Genom. Bioinform..

[32] Nyporko A., Voiteshenko I., Zarudnaya M., Hurmach V., Shyryna T., Platonov M., Roszak S., Rasulev B., Gorb L. (2025). SARS-CoV-2 RNA’s Dual Identity: G-Quadruplex versus Hairpin. ACS Omega.

[33] Wang S., Song Y., He Z., Saneyoshi H., Iwakiri R., Xu P., Zhao C., Qu X., Xu Y. (2023). Unusual topological RNA G-quadruplex formed by an RNA duplex: Implications for the dimerization of SARS-CoV-2 RNA. Chem. Commun..

[34] Campanile M., Improta R., Esposito L., Platella C., Oliva R., Del Vecchio P., Winter R., Petraccone L. (2024). Experimental and Computational Evidence of a Stable RNA G-Triplex Structure at Physiological Temperature in the SARS-CoV-2 Genome. Angew. Chem. Int. Ed..

[35] Kikin O., D’Antonio L., Bagga P.S. (2006). QGRS Mapper: A web-based server for predicting G-quadruplexes in nucleotide sequences. Nucleic Acids Res..

[36] Bedrat A., Lacroix L., Mergny J.-L. (2016). Re-evaluation of G-quadruplex propensity with G4Hunter. Nucleic Acids Res..

[37] Beaudoin J.-D., Jodoin R., Perreault J.-P. (2014). New scoring system to identify RNA G-quadruplex folding. Nucleic Acids Res..

[38] Warner K.D., Chen M.C., Song W., Strack R.L., Thorn A., Jaffrey S.R., Ferré-D’Amaré A.R. (2014). Structural basis for activity of highly efficient RNA mimics of green fluorescent protein. Nat. Struct. Mol. Biol..

[39] Xiao C.-D., Ishizuka T., Zhu X.-Q., Li Y., Sugiyama H., Xu Y. (2017). Unusual Topological RNA Architecture with an Eight-Stranded Helical Fragment Containing A-, G-, and U-Tetrads. J. Am. Chem. Soc..

[40] Guédin A., Lin L.Y., Armane S., Lacroix L., Mergny J.-L., Thore S., Yatsunyk L.A. (2018). Quadruplexes in ‘Dicty’: Crystal structure of a four-quartet G-quadruplex formed by G-rich motif found in the *Dictyostelium discoideum* genome. Nucleic Acids Res..

[41] Živković M.L., Rozman J., Plavec J. (2018). Adenine-Driven Structural Switch from a Two- to Three-Quartet DNA G-Quadruplex. Angew. Chem. Int. Ed..

[42] Trachman R.J., Autour A., Jeng S.C.Y., Abdolahzadeh A., Andreoni A., Cojocaru R., Garipov R., Dolgosheina E.V., Knutson J.R., Ryckelynck M. (2019). Structure and functional reselection of the Mango-III fluorogenic RNA aptamer. Nat. Chem. Biol..

[43] Escaja N., Mir B., Garavís M., González C. (2022). Non-G Base Tetrads. Molecules.

[44] Feng H., Kwok C.K. (2022). Spectroscopic analysis reveals the effect of hairpin loop formation on G-quadruplex structures. RSC Chem. Biol..

[45] Markham N.R., Zuker M. (2008). UNAFold. Bioinformatics.

[46] Halgren T.A., Murphy R.B., Friesner R.A., Beard H.S., Frye L.L., Pollard W.T., Banks J.L. (2004). Glide: A New Approach for Rapid, Accurate Docking and Scoring. 2. Enrichment Factors in Database Screening. J. Med. Chem..

[47] wwPDB consortium (2019). Protein Data Bank: The single global archive for 3D macromolecular structure data. Nucleic Acids Res..

[48] Abraham M.J., Murtola T., Schulz R., Páll S., Smith J.C., Hess B., Lindahl E. (2015). GROMACS: High performance molecular simulations through multi-level parallelism from laptops to supercomputers. SoftwareX.

[49] Huang J., MacKerell A.D. (2013). CHARMM36 all-atom additive protein force field: Validation based on comparison to NMR data. J. Comput. Chem..

[50] Fogolari F., Brigo A., Molinari H. (2003). Protocol for MM/PBSA Molecular Dynamics Simulations of Proteins. Biophys. J..

[51] Genheden S., Ryde U. (2015). The MM/PBSA and MM/GBSA methods to estimate ligand-binding affinities. Expert Opin. Drug Discov..

[52] Valdés-Tresanco M.S., Valdés-Tresanco M.E., Valiente P.A., Moreno E. (2021). gmx_MMPBSA: A New Tool to Perform End-State Free Energy Calculations with GROMACS. J. Chem. Theory Comput..

[53] Brown J.A. (2020). Unraveling the structure and biological functions of RNA triple helices. WIREs RNA.

[54] Cheong C., Moore P.B. (1992). Solution structure of an unusually stable RNA tetraplex containing G- and U-quartet structures. Biochemistry.

[55] Xu Y., Ishizuka T., Kimura T., Komiyama M. (2010). A U-Tetrad Stabilizes Human Telomeric RNA G-Quadruplex Structure. J. Am. Chem. Soc..

[56] Andrałojć W., Małgowska M., Sarzyńska J., Pasternak K., Szpotkowski K., Kierzek R., Gdaniec Z. (2019). Unraveling the structural basis for the exceptional stability of RNA G-quadruplexes capped by a uridine tetrad at the 3′ terminus. RNA.

[57] Andrałojć W., Pasternak K., Sarzyńska J., Zielińska K., Kierzek R., Gdaniec Z. (2020). The origin of the high stability of 3′-terminal uridine tetrads: Contributions of hydrogen bonding, stacking interactions, and steric factors evaluated using modified oligonucleotide analogs. RNA.

[58] Sun L., Li P., Ju X., Rao J., Huang W., Ren L., Zhang S., Xiong T., Xu K., Zhou X. (2021). In vivo structural characterization of the SARS-CoV-2 RNA genome identifies host proteins vulnerable to repurposed drugs. Cell.

[59] Huston N.C., Wan H., Strine M.S., de Cesaris Araujo Tavares R., Wilen C.B., Pyle A.M. (2021). Comprehensive in vivo secondary structure of the SARS-CoV-2 genome reveals novel regulatory motifs and mechanisms. Mol. Cell.

[60] Manfredonia I., Nithin C., Ponce-Salvatierra A., Ghosh P., Wirecki T.K., Marinus T., Ogando N.S., Snijder E.J., van Hemert M.J., Bujnicki J.M. (2020). Genome-wide mapping of SARS-CoV-2 RNA structures identifies therapeutically-relevant elements. Nucleic Acids Res..

[61] Miao Z., Tidu A., Eriani G., Martin F. (2021). Secondary structure of the SARS-CoV-2 5′-UTR. RNA Biol..

[62] Bassett M., Salemi M., Magalis B.R. (2022). Lessons Learned and Yet-to-Be Learned on the Importance of RNA Structure in SARS-CoV-2 Replication. Microbiol. Mol. Biol. Rev..

[63] Karousis E.D. (2024). The art of hijacking: How Nsp1 impacts host gene expression during coronaviral infections. Biochem. Soc. Trans..

[64] Shitaye G., Ventserova N., D’Abrosca G., Dragone M., Maina E.W., Fattorusso R., Iacovino R., Russo L., Isernia C., Malgieri G. (2025). The role of intrinsically disordered regions of SARS-CoV-2 nucleocapsid and non-structural protein 1 proteins. Front. Chem..

[65] Hoque M.E., Mahendran T., Basu S. (2022). Reversal of G-Quadruplexes’ Role in Translation Control When Present in the Context of an IRES. Biomolecules.

[66] Deforges J., de Breyne S., Ameur M., Ulryck N., Chamond N., Saaidi A., Ponty Y., Ohlmann T., Sargueil B. (2017). Two ribosome recruitment sites direct multiple translation events within HIV1 Gag open reading frame. Nucleic Acids Res..

[67] Zarudnaya M., Potyahaylo A.L., Kolomiets I.M., Gorb L.G. (2022). Genome sequence analysis suggests coevolution of the DIS, SD, and Psi hairpins in HIV-1 genomes. Virus Res..

[68] Kretsch R.C., Xu L., Zheludev I.N., Zhou X., Huang R., Nye G., Li S., Zhang K., Chiu W., Das R. (2024). Tertiary folds of the SL5 RNA from the 5′ proximal region of SARS-CoV-2 and related coronaviruses. Proc. Natl. Acad. Sci. USA.

[69] Hognon C., Miclot T., Garcı C., Francés-Monerris A., Grandemange S., Terenzi A., Marazzi M., Barone G., Monari A. (2020). Role of RNA Guanine Quadruplexes in Favoring the Dimerization of SARS Unique Domain in Coronaviruses. J. Phys. Chem. Lett..

[70] Tan J., Vonrhein C., Smart O.S., Bricogne G., Bollati M., Kusov Y., Hansen G., Mesters J.R., Schmidt C.L., Hilgenfeld R. (2009). The SARS-Unique Domain (SUD) of SARS Coronavirus Contains Two Macrodomains That Bind G-Quadruplexes. PLoS Pathog..

[71] Lavigne M., Helynck O., Rigolet P., Boudria-Souilah R., Nowakowski M., Baron B., Brülé S., Hoos S., Raynal B., Guittat L. (2021). SARS-CoV-2 Nsp3 unique domain SUD interacts with guanine quadruplexes and G4-ligands inhibit this interaction. Nucleic Acids Res..

[72] Qin B., Li Z., Tang K., Wang T., Xie Y., Aumonier S., Wang M., Yuan S., Cui S. (2023). Identification of the SARS-unique domain of SARS-CoV-2 as an antiviral target. Nat. Commun..

[73] Zhang Y.-H., Su A.-M., Hou X.-M. (2025). Structural and functional insights into the SARS-CoV-2 SUD domain and its interaction with RNA G-Quadruplexes. Biochem. Biophys. Res. Commun..

[74] Kusov Y., Tan J., Alvarez E., Enjuanes L., Hilgenfeld R. (2015). A G-quadruplex-binding macrodomain within the “SARS-unique domain” is essential for the activity of the SARS-coronavirus replication–transcription complex. Virology.

[75] Giuseppina M., Farthing R.J., Lale-Farjat S.L.M., Bergeron J.R.C. (2020). Structural Characterization of SARS-CoV-2: Where We Are, and Where We Need to Be. Front. Mol. Biosci..

[76] Brant A.C., Tian W., Majerciak V., Yang W., Zheng Z.-M. (2021). SARS-CoV-2: From its discovery to genome structure, transcription, and replication. Cell Biosci..

[77] Chen A., Lupan A.-M., Quek R.T., Stanciu S.G., Asaftei M., Stanciu G.A., Hardy K.S., Magalhães T.d.A., Silver P.A., Mitchison T.J. (2024). A coronaviral pore-replicase complex links RNA synthesis and export from double-membrane vesicles. Sci. Adv..

[78] Yang J., Tian B., Wang P., Chen R., Xiao K., Long X., Zheng X., Zhu Y., Sun F., Shi Y. (2025). SARS-CoV-2 NSP3/4 control formation of replication organelle and recruitment of RNA polymerase NSP12. J. Cell Biol..

[79] Lei J., Ma-Lauer Y., Han Y., Thoms M., Buschauer R., Jores J., Thiel V., Beckmann R., Deng W., Leonhardt H. (2021). The SARS-unique domain (SUD) of SARS-CoV and SARS-CoV-2 interacts with human Paip1 to enhance viral RNA translation. EMBO J..

[80] Lemak S., Skarina T., Patel D.T., Stogios P.J., Savchenko A. (2025). Structural and functional analyses of SARS-CoV-2 Nsp3 and its specific interactions with the 5′ UTR of the viral genome. Microbiol. Spectr..

[81] Roe M.K., Junod N.A., Young A.R., Beachboard D.C., Stobart C.C. (2021). Targeting novel structural and functional features of coronavirus protease nsp5 (3CLpro, Mpro) in the age of COVID-19. J. Gen. Virol..

[82] Aruda J., Grote S.L., Rouskin S. (2024). Untangling the pseudoknots of SARS-CoV-2: Insights into structural heterogeneity and plasticity. Curr. Opin. Struct. Biol..

[83] Rajpal V.R., Sharma S., Sehgal D., Singh A., Kumar A., Vaishnavi S., Tiwari M., Bhalla H., Goel S., Raina S.N. (2022). A Comprehensive Account of SARS-CoV-2 Genome structure, Incurred mutations, Lineages and COVID-19 Vaccination Program. Future Virol..

[84] Fleming A.M., Mathewson N.J., Manage S.A.H., Burrows C.J. (2021). Nanopore Dwell Time Analysis Permits Sequencing and Conformational Assignment of Pseudouridine in SARS-CoV-2. ACS Cent. Sci..

[85] Izadpanah A., Rappaport J., Datta P.K. (2022). Epitranscriptomics of SARS-CoV-2 Infection. Front. Cell Dev. Biol..

[86] Wu R., Luo K.Q. (2021). Developing effective siRNAs to reduce the expression of key viral genes of COVID-19. Int. J. Biol. Sci..

[87] Wu W., Cheng Y., Zhou H., Sun C., Zhang S. (2023). The SARS-CoV-2 nucleocapsid protein: Its role in the viral life cycle, structure and functions, and use as a potential target in the development of vaccines and diagnostics. Virol. J..

[88] Estelle A.B., Forsythe H.M., Yu Z., Hughes K., Lasher B., Allen P., Reardon P.N., Hendrix D.A., Barbar E.J. (2023). RNA structure and multiple weak interactions balance the interplay between RNA binding and phase separation of SARS-CoV-2 nucleocapsid. PNAS Nexus.

[89] Vianney Y.M., Klaus W. (2022). High affinity binding at quadruplex-duplex junctions rather the rule than the exception. Nucleic Acid Res..

[90] Ruggiero E., Richter S.N. (2018). G-quadruplexes and G-quadruplex ligands: Targets and tools in antiviral therapy. Nucleic Acids Res..

[91] Babot M., Boulard Y., Agouda S., Pieri L., Fieulaine S., Bressanelli S., Gervais V. (2024). Oligomeric assembly of the C-terminal and transmembrane region of SARS-CoV-2 nsp3. J. Virol..

[92] Huang Y., Wang T., Zhong L., Zhang W., Zhang Y., Yu X., Yuan S., Ni T. (2024). Molecular architecture of coronavirus double-membrane vesicle pore complex. Nature.

[93] Kelly J.A., Olson A.N., Neupane K., Munshi S., Emeterio J.S., Pollack L., Woodside M.T., Dinman J.D. (2020). Structural and functional conservation of the programmed −1 ribosomal frameshift signal of SARS coronavirus 2 (SARS-CoV-2). J. Biol. Chem..

