# Supplementary material for: In Search of the Most Significant Potential G-Quadruplexes in SARS-CoV-2 RNA: Genomic Analysis
Source: Viruses. 2026 Feb 16;18(2):253. doi: 10.3390/v18020253 (PMC12945054; doi:10.3390/v18020253)
Supplement: Supplementary file 1 [file viruses-18-00253-s001.zip › viruses-4097896-supplementary.pdf]

# Supplementary information

**to**

**In search of the most significant potential G-quadruplexes in SARS-CoV-2 RNA, genomic analysis.**

Margarita Zarudnaya, Ivan Voiteshenko, Vasyl Hurmah, Tetiana Shyryna, Alex Nyporko, Maksym Platonov, Szczepan Roszak, Bakhtiyor Rasulev, Karina Kapusta and Leonid Gorb

# Tables

### Table S1

Biophysical assays used to validate G4 formation of PQSs in SARS-CoV-2 genomic RNA*.

| PQS | Assay | | | | | | |
|---|---|---|---|---|---|---|---|
| | **CD** | **Fluorescence** | **FRET** | **NMR** | **TDS** | **UV melting** | **PAGE** |
| **644** | [13], [18], [22], [27] | [13], [18], [22], [27] | nd | [13] | [13] | nd | nd |
| **1574** | [18], [21], [27] | [18], [27] | [21] | nd | nd | nd | nd |
| **3467** | [13], [18], [20], [24], [27] | [13], [18], [27] | nd | [13], [18], [20], [24] | [13], [24] | nd | [18] |
| **8687** | [27] | [18], [27] | nd | nd | nd | nd | nd |
| **13385** | [18], [19], [21], [22], [23], [25], [26], [27], [28] | [18], [22], [23], [25], [27], [28] | [21] | [19] | [19] | [23] | [28] |
| **24215** | [22], [27] | [22], [27] | nd | nd | nd | nd | nd |
| **24268** | [22], [23], [25], [26], [27] | [18] [22], [23], [25], [27], [28] | nd | nd | nd | [23] | nd |
| **25197** | [22], [27] | [22], [27], [28] | nd | nd | nd | nd | nd |
| **28903** | [13], [17], [18], [19], [20], [21], [24], [26], [27], [28] | [13], [18], [19], [27], [28] | [17], [18], [21], [28] | [13], [19], [20], [28] | [13], [19], [24] | nd | [28] |

* Razzak et al. [27] conducted CD and fluorescence assays for 16 more PQSs (353, 1463, 2714, 4162, 4261, 10261, 14947, 15208, 15448, 18296, 22316, 25951, 26746, 28781, 29123, 29234). The authors confirmed G4 formation for all 25 PQSs studied. Only in [22], PQSs were taken with the flanking nucleotides; however for PQS 28903 they were not natural, therefore authors data for this PQS was not included into Table. ** Refs in which the assay does not confirm G4 formation are indicated by red. CD – circular dichroism spectroscopy, FRET - Forster Resonance Energy Transfer, NMR – nuclear magnetic resonance, PAGE – polyacrylamide gel electrophoresis, TDS – thermal differential spectroscopy, UV – ultraviolet. Nd – not determined.

## Table S2

**A.** PQSs in positive sence SARS-CoV-2 gRNA.

| 1 | 2 | 3 | 4 | 5 | 6 |
|---|---|---|---|---|---|
| **PQS*** | **Gene** | **Sequence** | **Leng-th, nts** | **ΔG of hairpin, kcal/mol** | **Cap** |
| **236** | 5' UTR | aggguuucguccgggugugaccgaaagguaagaugga | 34 | -8,2 | |
| **236a*** | 5' UTR | agguuucguccgggugugaccgaaagguaagauggag | 34 | -9,8 | 3' uau |
| **236el*** | 5' UTR | uagguuucguccggguguggaccgaaagguaagauggagagccuugucccugguu | 51 | -15,5 | 3' uuuu |
| **353** | nsp1 | guggcuuuggagacuccguggagga | 22 | -3,4 | |
| **S53el** | nsp1 | guggcuuuggagacuccguggaggaggu | 26 | -6,0 | 5' uau |
| **359** | nsp1 | uggagacuccguggaggaggu | 19 | -6,0 | |
| **370** | nsp1 | uggaggaggucuuaucagaggc | 20 | +0,1 | |
| **508*** | nsp1 | auggucauguuaugguugagcugguagcagaacucgaaggc | 39 | -4,9 | 5' uau |
| **509*** | nsp1 | uggucauguuaugguugagcugguagcagaacucgaaggcauucaguacgguc | 51 | -6,2 | 3' uuuu |
| **529*** | nsp1 | ugguagcagaacucgaaggcauucaguacggucguaguggu | 39 | -4,5 | |
| **529el*** | nsp1 | cugguagcagaacucgaaggcauucaguacggucguaguggugagacacuuggugu | 52 | -9,2 | 3' uuuu |
| **545** | nsp1 | aaggcauucaguacggucguaguggugagacacuuggug | 35 | -4,9 | |
| **644** | nsp1 | cgguaauaaaggagcugguggc | 20 | +1,1 | |
| **653*** | nsp1 | aggagcugguggccauaguuacggc | 23 | -1,9 | |
| **653m*** | nsp1 | aaggagcugguggccauaggua | 18 | -0,7 | |
| **659m*** | nsp1 | ugguggccauagguacggc | 17 | -1,2 | |
| **1463** | nsp2 | aggguggucgcacuauugccuuuggaggc | 26 | -5,1 | |
| **1558*/** | nsp2 | uagguuguaaccauacagguguuguuggagaagguuccgaaggu | 42 | -6,0 | 5' aaaa / |
| **1559*** | nsp2 | agguuguaaccauacagguguuguuggagaagguuccgaagguc | 42 | -6,0 | 3' uuuu |
| **1574** | nsp2 | cagguguuguuggagaagguuccgaaggu | 26 | -4,0 | |
| **1574a*** | nsp2 | cagguguuguuggagaagguuccgaaggu | 26 | -4,0 | 3' uau |
| **1784*** | nsp2 | gugguaauuuuaaaguuacaaaaggaaaagcuaaaaaaggugccuggaa | 46 | -5,1 | 3' uaua |
| **1804/** | nsp2 | aaggaaaagcuaaaaaaggugccuggaauauuggug | 33 | -1,9 | 5' aauu |
| **1805a*** | nsp2 | aggaaaagcuaaaaaaggugccuggaauauuggug | 33 | -1,9 | 3' auau |
| **1805** | nsp2 | aggaaaagcuaaaaaaggugccuggaauauuggug | 32 | -1,9 | |
| **2714** | nsp2 | aggcggugcaccaacaaagguuacuuuuggug | 29 | -4,7 | |
| **2717** | nsp2 | cggugcaccaacaaagguuacuuuuggugaugacacugugauagaagugcaagguu | 54 | -12,1 | 3' uuuu |
| **3467** | nsp3 | auggaggagguguugcagga | 17 | +1,1 | |
| **4127a*/** | nsp3 | ugggugauguuguucaagaggguguuuuaacugcugugguuauaccuacuaaaaggcuggu | 60 | -8,6 | 5' uau/ |
| **4127b*** | nsp3 | gggugauguuguucaagaggguguuuuaacugcugugguuauaccuacuaaaaggcuggug | 60 | -8,6 | 3' uuuu |
| **4143*** | nsp3 | gaggguguuuuaacugcugugguuauaccuacuaaaaaggcuggug | 43 | -7,2 | 5' auau |
| **4161*** | nsp3 | ugugguuauaccuacuaaaaaggcugguggc | 28 | -3,7 | 5' uau |
| **4256*** | nsp3 | gggucaggguuuaaaugguuacacuguagagga | 31 | -1,0 | |
| **4256a*** | nsp3 | gggucaggguuuaaaugguuacacuguagaggag | 32 | -1,0 | 3' uau |
| **4262** | nsp3 | aggguuuaaaugguuacacuguagaggaggc | 28 | +0,1 | |
| **4485*/** | nsp3 | uaaggguauuaaaauacaagaggguggugguugauuauggu | 37 | -3,0 | 5' aauu/ |
| **4487** | nsp3 | ggguauuaaaauacaagaggguggugguugauuauggug | 36 | -3,0 | 3' uuuu |
| **4616** | nsp3 | uggcuauguaacacauggcuuaaauuuggaagaagcugcucggu | 42 | -8,1 | |
| **5036** | nsp3 | guggacaacaguuugguccaacuuauuuggaugga | 32 | -5,3 | |
| **7213*** | nsp3 | ugggauuuaacugcuuuuggcuuaguugcagagugguuuuuggc | 41 | -8,2 | |
| **7228*** | nsp3 | uuggcuuaguugcagagugguuuuuggcauauauucuuuucacuaggu | 46 | -6,5 | 5' uau |
| **7245*** | nsp3 | gugguuuuuggcauauauucuuuucacuagguuuuucuauguacuugga | 46 | -3,4 | 5' uau |
| **7245a*** | nsp3 | ugguuuuuggcauauauucuuuucacuagguuuuucuauguacuuggau | 47 | -3,4 | 3' uau |

| | | | | | |
|---|---|---|---|---|---|
| **7252*** | nsp3 | uuggcauauauucuuuucacuagguuuuucuauguacuuggauuggc | 45 | -3,4 | 5' uau |
| **8687** | nsp4 | uaggauacaaggcuauugauggugguu | 23 | -0,6 | |
| **10058*** | nsp5 | gugguuuuagaaaaauggcauucccaucugguaaaguugaggg | 41 | -3,1 | 5' uau |
| **10072*** | nsp5 | uggcauucccaucugguaaaguugaggguuguauggu | 35 | -6,9 | 5 uau |
| **10084*/** | nsp5 | cuugguaaaguugaggguuguaugguacaaguaacuuguggu | 39 | -4,6 | 5' uau/ |
| **10085*** | nsp5 | cugguaaaguugaggguuguaugguacaaguaacuuguggua | 39 | -4,6 | 3 uuuu |
| **10095*** | nsp5 | ugaggguuguaugguacaaguaacuugugguacaacuacacuuaacggu | 46 | -11,6 | 5' uau/ |
| **10097*** | nsp5 | aggguuguaugguacaaguaacuugugguacaacuacacuuaacgguc | 45 | -11,6 | 3' uuuu |
| **10254*** | nsp5 | uugguacaggcugguaauguucaacucagggu | 29 | -2,2 | 5' uaua |
| **10255** | nsp5 | ugguacaggcugguaauguucaacucagggu | 28 | -2,2 | |
| **10260a*** | nsp5 | caggcugguaauguucaacucaggguuauugga | 31 | -3,6 | 5' auau |
| **10261** | nsp5 | aggcugguaauguucaacucaggguuauugga | 30 | -3,6 | |
| **10466** | nsp5 | uaaggguucauuccuuaaugguucaugugguaguguuggu | 35 | -5,7 | |
| **10464*** | nsp5 | uaaggguucauuccuuaaugguucaugugguaguguuggu | 37 | -5,7 | 5' uau. |
| **10466a*** | nsp5 | auuaagg**gu**ucauuccuuaau**ggu**ucaugu**ggu**aguguu**ggu**uuuaac | 36 | -7,8 | 3' uuuu |
| **10548*** | nsp5 | uauggaauuaccaacuggaguucaugcuggcacagacuuagaaggu | 43 | -6,4 | 5' uau |
| **10562*** | nsp5 | cuggaguucaugcuggcacagacuuagaagguaacuuuuaugga | 41 | -5,5 | 5' uau |
| **10573*** | nsp5 | cuggcacagacuuagaagguaacuuuuauggaccuuuuguugacaggc | 46 | -5,8 | 5' uaua |
| **10588*** | nsp5 | uagaaagguaacuuuuauggaccuuuuguugacaggcaaacagcacaagcagcuggu | 51 | -9,7 | 5' auau |
| **10600*** | nsp5 | auggaccuuuuguugacaggcaaacagcacaagcagcugguacgga | 44 | -8,5 | 5' uau |
| **10674*** | nsp5 | cuugguuguacgcugcuguuauaaauggagacaggugguu | 36 | -5,3 | 5' uau |
| **11221*** | nsp6 | uggucuauaugccugcuaguuggguгaugcguauuaugacaugguuggauaugguu | 54 | -10,7 | 3' uuuu |
| **11242*** | nsp6 | ugggugaugcguauuaugacaugguuggauaugguugauacuaguuugucugguu | 52 | -7,9 | 3' uuuu |
| **12961*/** | nsp9 | aaggauuaaacaaccuaaauagagguaugguacuuggua | 36 | -3,6 | 5' aauu/ |
| **12962*** | nsp9 | aggauuaaacaaccuaaauagagguaugguacuuggua | 36 | -3,6 | 3' uau |
| **13172*** | nsp10 | cugguacuggucaggcaauaacaguuacaccgga | 32 | -5,1 | 5' uau |
| **13172a*** | nsp10 | cugguacuggucaggcaauaacaguuacaccggaa | 32 | -5,1 | 3' uau |
| **13201** | nsp10 | cggaagccaauauggaucaagaauccuuugguggu | 33 | -7,0 | |
| **13201a*** | nsp10 | accggaagccaauauggaucaagaauccuuuggugguug | 34 | -7,7 | 3' aauu |
| **13385** | nsp10 | cgguaugugggaaagguuauggc | 20 | +1,9 | |
| **13385a*** | nsp10 | cgguauguggaaagguuauggc | 20 | +1,9 | 5' uau |
| **13385b*** | nsp10 | cgguauguggaaagguuauggc | 20 | +1,9 | 3' uau |
| **14946*** | nsp12 | cugguuuuccauuuaauaaaugggguaaggc | 29 | -3,9 | 5' uau |
| **14947** | nsp12 | ugguuuuccauuuaauaaaugggguaaggc | 28 | -3,9 | |
| **15208** | nsp12 | uggaacaagcaaauucuaugguugguuggc | 27 | -1,5 | |
| **15437*** | nsp12 | auggucaugugugggcgguucacuauauguuaaaccaggug | 37 | -8,1 | 5' uau |
| **15448** | nsp12 | guggcgguucacuauauguuaaaccaggugga | 29 | -2,7 | |
| **15451*** | nsp12 | cgguucacuauauguuaaaccagguggaaccucaucaggaga | 39 | -6,5 | 3' uuaa |
| **18296** | nsp14 | uggauuggcuucgaugucgaggggu | 23 | -5,4 | |
| **20869** | nsp16 | uggugcugguucugauaaaggaguugcaccaggu | 32 | -11,4 | |
| **20868*/** | nsp16 | uuggugcugguucugauaaaggaguugcaccaggu | 33 | -12,1 | 5' uuaa/ |
| **20869a*** | nsp16 | uggugcugguucugauaaaggaguugcaccagguac | 34 | -11,4 | 3' uau |
| **20868el*** | nsp16 | uggugcugguucugauaaaggaguugcaccagguacagcuguuuuaagacagugguu | 55 | -17.1 | 3 uuuu |
| **20874*/** | nsp16 | cugguucugauaaaggaguugcaccagguacagcuguuuuaagacagauggu | 49 | -8,2 | 5' uaau/ |
| **20875*** | nsp16 | ugguucugauaaaggaguugcaccagguacagcuguuuuaagacagugu | 48 | -7,5 | 3' uau |
| **20887*** | nsp16 | aggaguugcaccagguacagcuguuuuaagacagugguugccuacgggua | 48 | -13,7 | 3 uau |
| **22216** | S | aggguuuuucggcuuuagaaccauugguagauuugccaauaggu | 42 | -9,3 | |
| **22316** | S | uggugauucuucuucagguuggacagcuggu | 29 | -1,8 | |
| **22315*** | S | cuggugauucuucuucagguuggacagcuggu | 30 | -1,8 | 5' uau |

| | | | | | |
|---|---|---|---|---|---|
| **22316a*** | S | uggugauucuucuucagguuggacagcuggugc | 30 | -1,8 | 3' uau |
| **22316el*** | S | uggugauucuucuucagguuggacagcuggugcugcagcuuauuaugugggguu | 51 | -9,4 | 3' uuuu/ |
| **22315el*** | S | cuggugauucuucuucagguuggacagcuggugcugcagcuuauuauguggggu | 51 | -9,4 | 5' uau |
| **22330*** | S | cagguuggacagcuggugcugcagcuuauuaugugggu | 35 | -9,4 | 5' uau |
| **22331*** | S | cagguuggacagcuggugcugcagcuuauuaugugggu | 35 | -9,4 | 3' uau |
| **24200*/** | S | cgguacaaucacuucugguuggaccuuuggugcaggu | 35 | -9,2 | 5' uau/ |
| **24200a*** | S | ggguacaaucacuucugguuggaccuuuggugcaggug | 36 | -8,2 | 3' uuuu |
| **24214*** | S | cugguuggaccuuuggugcaggu | 21 | -3,8 | 5' uau |
| **24215a*** | S | ugguuggaccuuuggugcaggu | 20 | -3,8 | 3' uau |
| **24215** | S | ugguuggaccuuuggugcaggu | 20 | -3,8 | |
| **24267*** | S | auggcuuauagguuuaaugguauugga | 25 | -0,2 | 5' uau |
| **24268** | S | auggcuuauagguuuaaugguauuggag | 24 | -0,2 | |
| **25196*** | S | uggccaugguacauuuggcuaggu | 22 | -5,8 | |
| **25197** | S | uggccaugguacauuuggcuaggu | 22 | -5,8 | 5' uau |
| **25203*** | S | ugguacauuuggcuagguuuuauagcuggc | 28 | -5,7 | 5' uau |
| **25951** | ORF3a | auuggugguuauacugaaaaaugggaaucuggag | 29 | -2,3 | |
| **26746** | M | uggaucaccgguggaauugcuaucgcaauggc | 30 | -4,8 | |
| **26754*** | M | cgguggaauugcuaucgcaauggcuugucuuguaggc | 35 | -5,8 | |
| **26757*** | M | uggaauugcuaucgcaauggcuugucuuguaggcuugauguggc | 44 | -8,6 | 5' uau |
| **28346** | N | uggcaguaaccagaauggagaacgcaguggggc | 31 | -2,4 | |
| **28346d*** | N | uggcaguaaccagaauggugggc | 22 | -2,3 | |
| **28612*** | N | uaggaacugggccagaagcuggacuucccuauggu | 33 | -6,1 | 5' uau |
| **28613** | N | uaggaacugggccagaagcuggacuucccuauggu | 32 | -6,1 | |
| **28619** | N | ugggccagaagcuggacuucccuauggugcuaacaaagacggc | 41 | -6,0 | |
| **28620*** | N | ugggccagaagcuggacuucccuauggugcuaacaaagacggc | 40 | -6,0 | |
| **28631*** | N | uggacuucccuaugguguaacaaagacggcaucauaugggguu | 41 | -14,5 | 3' uau |
| **28642*** | N | auggugcuaacaaagacggcaucauaugggguugcaacugagggag | 41 | -6,6 | 5' uau/ |
| **28643*** | N | auggugcuaacaaagacggcaucauaugggguugcaacugagggag | 42 | -6,6 | 3' uau |
| **28781** | N | aggcuucuacgcagaagggagcagaggcggc | 29 | -8,3 | |
| **28903** | N | uggcuggcaauggcggu | 15 | -2,5 | |
| **29104*** | N | gugguccagaacaaacccaaggaaauuuuggggaccagga | 38 | -10,1 | 5' uaua |
| **29123** | N | aggaaauuuuggggaccagga | 19 | -0,6 | |
| **29123el*** | N | aggaaauuuuggggaccaggaacuaaucagacaaggaa | 36 | -1,5 | 3' aaaa |
| **29132*** | N | uggggaccaggaacuaaucagacaaggaac | 26 | +0,5 | |
| **29219*** | N | cggaaugucgcgcauuggcauggaagucacaccuucggga | 37 | -6,5 | |
| **29234** | N | uggcauggaagucacaccuucgggaacguggu | 30 | -5,7 | |
| **29238*** | N | cauggaagucacaccuucgggaacgugguugaccuacacaggu | 40 | -10,1 | 5' uau/ |
| **29239*** | N | uggaagucacaccuucgggaacgugguugaccuacacaggugc | 40 | -9,8 | 3' aauu |
| **29254** | N | cgggaacgugguugaccuacacaggugccaucaaauugga | 38 | -7,9 | |
| **29255*** | N | cgggaacgugguugaccuacacaggugccaucaaauuggau | 38 | -7,9 | 3' auua/ |
| **29255a*** | N | gggaacgugguugaccuacacaggugccaucaaauugga | 37 | -7,9 | 5' uau |
| **Sum** | 133 | | | | |

Column 1: *not reported in literature; overlapping PQSs are marked in the same colors. Column 3: GG repeats (together with other bases) in PQS stabilized by tetrads or triads are indicated by pink color, those in unstabilized PQSs are indicated by red colour. Column 3: sequences of the internal hairpins in G4 loops are indicated by grey. Column 4: PQS length without flanking nucleotides. Column 5: the most unstable hairpins are marked in yellow. Column 6: potential G4 capped by tetrads or triads at both ends are marked in green or blue. ΔG of internal hairpins: 236el – -1.2 kcal/mol; 509 – -1.9 kcal/mol; 2717el – 4.2 kcal/mol; 20869el – -5.4 kcal/mol.

**B.** PQSs in negative sence SARS-CoV-2 gRNA.

| 1 | 2 | 3 | 4 | 5 | 6 |
|---|---|---|---|---|---|
| **PQS***<br>**-/+** | **Gene** | **Sequence** | **Len-gth, nts** | **ΔG of hairpin, kcal/mol** | **Cap** |
| **165/29739** | 3' UTR | uggccucggugaaaaugugguggc | 22 | -2,8 | uau in loop |
| **1358/28546*** | N | ugguagcucuucgguaguagccaauuuggucaucuggacugcuauuggugu | 48 | -12,9 | 3' uuuu |
| **1384/28520*** | | uggucaucuggacugcuauugguguuaauuggaac | 32 | -3,2 | 3' uaua |
| **13136/16768** | nsp13 | cgguuaagugguggucuagguu | 19 | +0,7 | |
| **13944/15960*** | nsp12 | cggccccuaggauucuugauggaucugggu | 27 | -9,1 | |
| **13952/15952*** | | cuaggauucuugauggaucuggguaagga | 26 | -5,0 | 5' auua |
| **13963/15941** | | ugauggaucuggguaaggaaggu | 19 | -0,2 | 5' uuaa |
| | | gauggaucuggguaaggaaggua | 19 | -0,6 | 3' auau |
| **16623/13281** | nsp10 | uaggauuuggaugaucuaugugcaacggc | 28 | -3,6 | 5' uau |
| **19865/10039** | nsp4 | aggugauagagguuuguggugguug | 21 | +1,6 | |
| **19874/10030** | | agguuuguggugguuggua | 16 | +4,3 | |
| **23877/6027** | nsp3 | guuuggauaugguugguuuggua | 18 | -0,6 | 3' uau |
| **25003/4901** | | uagguggaaugugguaggauu | 16 | +3,3 | 3' uaua |
| **27432/2472** | nsp2 | uggggcuuuuagaggcaugaguaggc | 24 | -2,7 | |
| **28690/1214*** | | aaggcacauugguugcauucauuuggugacgcaacugga | 38 | -9,1 | 5' uau |
| **29867/37** | 5' UTR | uugguugguuuguuaccugggaaggu | 23 | -1,9 | |
| | | uugguugguuuguuaccugggaaggu | 24 | -1,9 | 5' uau |

Column 1: Localization is indicated both in minus and plus chain. *not reported in literature; Column 2: GG repeats (together with other bases) in PQS stabilized by tetrads or triads are indicated by pink color, those in unstabilized PQSs are indicated by red color. Column 4 Column 5: the most unstabled hairpins are marked in yellow. Column 6: potential G4 capped by tetrads or triads at both ends are marked in green.

**C.** Putative G-triplex sequences in SARS-CoV-2 genomic RNA

| **PQS** | **Gene** | **Sequence** | **Length** | **h** |
|---|---|---|---|---|
| **4179** | nsp3 | aggcugguggc | 9 | +3,4 |
| **6158** | nsp3 | uggugaugugguggc | 13 | +4,1 |
| **6358** | nsp3 | aggacgcgcagggaaugga | 17 | +1,0 |
| **7583** | nsp3 | uggagguaaaggc | 11 | - |
| **10697** | nsp5 | uggagacagguggu | 12 | - |
| **11375** | nsp6 | auggugcuaggagaguggga | 19 | -0,9 |
| **11800** | nsp6 | ggguguugguggc | 11 | +2,5 |
| **12793** | nsp9 | gggagguaggu | 9 | - |
| **21869** | S | gaggcuggauuuuuggu | 15 | +1,2 |
| **23195** | S | ugguuuaacaggcacaggu | 17 | +1,1 |
| **25769** | ORF3a | gaggcuuggcuuugcugga | 18 | -2,2 |
| **28079** | ORF8 | guggaugaggcuggu | 13 | +1,7 |
| **28559** | N | ugguggugacggu | 11 | -1,5 |

GG repeats (together with other bases) in PQS stabilized by tetrads or triads are indicated by pink color, those in unstabilized PQSs are indicated by red color.

**Table S3**

Mutational analysis of the SARS-CoV-2 5' terminal region (%).

| | **Country** | **China** | **India** | **Ukraine** | **UK** | **USA** |
|---|---|---|---|---|---|---|
| **Year** | | **Numbers of genomes** | | | | |
| **2022** | **Mutation** | **3823** | **4070** | **370** | **7573** | **7964** |
| | **C21U** | 2 | 0 | 0.3 | 0.04 | 0.02 |
| | **C44U** | 26 | 5 | 5 | 0 | 7 |
| | **C193U** | 0.05 | 0.05 | 0 | 8,0 | 0.1 |
| | **C203U** | 0.1 | 0.1 | 2 | 0.3 | 0.4 |
| | **G204A** | 0.05 | 1 | 0,5 | 0.07 | 0.1 |
| | **G210U** | 15 | 2 | 0 | 0 | 0.1 |
| | **C241U** | 55 | 88 | 81 | 74 | 80 |
| | **C355U** | 0 | 0.02 | 2 | 0.04 | 0.04 |
| | **Country** | **China** | **India** | **Ukraine** | **UK** | **USA** |
| **Year** | | **Numbers of genomes** | | | | |
| **2023** | **Mutation** | **4239** | **1028** | **2182** | **8556** | **7207** |
| | **U10A** | 0 | 0 | 0 | 0 | 2 |
| | **U13A** | 0 | 0 | 0 | 0 | 2 |
| | **C21U** | 0.,05 | 0 | 0 | 12 | 3 |
| | **C44U** | 52 | 2 | 0.2 | 13 | 9 |
| | **C193U** | 0.02 | 0 | 2 | 0.06 | 0.,2 |
| | **G210U** | 9 | 0.1 | 0 | 0.01 | 0.01 |
| | **218U** | 0,02 | 0 | 6 | 1 | 0,4 |
| | **C241U** | 32 | 90 | 84 | 66 | 75 |
| | **Country** | **China** | **India** | **Ukraine** | **UK** | **USA** |
| **Year** | | **Numbers of genomes** | | | | |
| **2024** | **Mutation** | **2232** | **1272** | **767** | **3415** | **1013** |
| | **C21U** | 20 | 2 | 0 | 0.3 | 16 |
| | **C44U** | 51 | 0 | 0 | 1 | 11 |
| | **U111C** | 2 | 0.2 | 0.3 | 0.2 | 0.3 |
| | **C228U** | 0 | 4 | 0 | 0.3 | 0 |
| | **C241U** | 34 | 87 | 81 | 89 | 70 |

Only mutations occurring with the frequency ≥2% (in at least one of the countries) are presented in the Table. Mutations that occurr with frequencies of ≥2%, ≥15% and ≥50% are indicated by yellow, blue and green color, respectively.

Mutation research

auuaaagguuuauaccuucccagguaacaaaccaaccaacuuucgaucucuuguagaucuguucucuaaacgaacuuuaaaaucugu
guggcugucacucggcugcaugcuuagugcacucacgcaguauaauuaauaacuaauuacugucguugacaggacacgaguaacucg
ucuaucuucugcaggcugcuuacgguuucguccguguugcagccgaucaucagcacaucuagguuucguccggguguga ccgaaagg
uaagAUGgagagccuugucccugguuucaacgagaaaacacacguccaacucaguuugccuguuuuacagguucgcgacgugcucgu
acgugg cuuuggagacuccguggaggaggucuuaucagaggcacgucaaca

GG-repeats are indicated by red. CC-repeats, corresponding to GG-repeats in minus genomic RNA chain are indicated by brown. AUG - start codon. The most frequent mutations are indicated by color.

## Table S4

Comparison of PQSs in SARS-CoV and SARS-CoV-2 genomic RNAs.

| Line | PQS in SARS-CoV-2 | Sequence | SARS |
|---|---|---|---|
| 1 | 236a | agguuucguccggugugaccgaaagguaagAUGgag<br>agguuucguccggugugaccgaaagguaagAUGgag | CoV<br>CoV-2 |
| 2 | 236el | agguuucguccggugugaccgaaagguaagAUGgagagccuuguucuuggug<br>agguuucguccggugugaccgaaagguaagAUGgagagccuuguccuggu | CoV<br>CoV-2 |
| 3 | SL7 (353-370) | Gcguggcuucggggacucuguggaagaggcccuaucggaggcacgu<br>acguggcuuuggagacuccguggaggagguccuuaucagaggcacgu | CoV<br>CoV-2 |
| 4 | 509 | cggccacaaggucguugagcugguugcagaaauggacggcauucaguacggucggua<br>uggucauguuaugguugagcugguagcagaacucgaaggcauucaguacggucguag | CoV<br>CoV-2 |
| 5 | 644 | cgguaauaagggagccgguggu<br>cgguaauaaaggagcugguggc | CoV<br>CoV-2 |
| 6 | 653 | gggagccgguggucauagcuauggc<br>aggagcugguggccauaguuacggc | CoV<br>CoV-2 |
| 7 | 1463 | agggaggtaggactagatgttttggaggc<br>agggtggtcgcactattgcctttggaggc | CoV<br>CoV-2 |
| 8 | 1784 | gcgguaacuauaaaguuaccaagggaaagcccguaaaaggugcuuggac<br>gugguaauuuuaaaguuacaaaaggaaaagcuaaaaaaggugccuggaa | CoV<br>CoV-2 |
| 9 | 1804 | aagggaaagcccguaaaaggugcuuggaacauuggac<br>aaggaaaagc-uaaaaaaggugccuggaauauuggug | CoV<br>CoV-2 |
| 10 | 2714 | agggggugcaccaauuaaagguguaaccuuuggag<br>aggcggugcaccaa-caaaggu-ua-cuuuuggug | CoV<br>CoV-2 |
| 11 | 4256 | cuggacaaggaugugcugguuauacacuugaggaa<br>cggguucaggguuuaaauggauuacacuguagaggag | CoV<br>CoV-2 |
| 12 | 8687 | ugguuacaaagccauucaggauggu<br>aggauacaaggcuauugauggu ggu | CoV<br>CoV-2 |
| 12 | 10085 | caggcaaaguugaaggugcaugguacaaguaaccuguggaa<br>cugguaaaguugaggguuguauggucacaaguaacuuguggua | CoV<br>CoV-2 |
| 13 | 10254 | cuuguucaggcuggcaauguucaacuucgug<br>uugguacaggcugguaauguucaacucaggg | CoV<br>CoV-2 |
| 14 | 10260 | caggcuggcaauguucaacuucguguuauuggcc<br>caggcugguaauguucaacucaggguuauuggac | CoV<br>CoV-2 |
| 15 | 10466 | uaaagguucuuuccuuaauggaucaugugguaguguugguu<br>uaaggguucauuccuuaaugguucaugugguaguguugguu | CoV<br>CoV-2 |
| 16 | 10548 | uauggagcuuccaacaggaguacacgcugguacugacuuagaaggu<br>uauggaauuaccaacuggaguucaugcuggcacagacuuagaaggu | CoV<br>CoV-2 |

| | | | |
|---|---|---|---|
| **17** | **10562** | caggaguacacgcugguacugacuuagaagguaaauucuauggu<br>cuggaguucaugcuggcacagacuuagaagguaacuuuuaugga | CoV<br>CoV-2 |
| **18** | **10573** | cugguacugacuuagaagguaaauucuaugguccauuuguugacagac<br>cuggcacagacuuagaagguaacuuuuauggaccuuuuguugacaggc | CoV<br>CoV-2 |
| **19** | **10674** | uggcuguaugcugcuguuaucaauggugauagguggau<br>ugguuguacgcugcuguuauaaauggagacagguggu | CoV<br>CoV-2 |
| **20** | **13385** | cggaauguggaaagguuauggc<br>cgguauguggaaagguuauggc | CoV<br>CoV-2 |
| **21** | **15941** | accugccuuacccagaucca 1<br>accuuccuuacccagaucca 2 | CoV<br>CoV-2 |
| **22** | **24200/24215** | ggguacugccacugcuggauggacauuuggugcuggcg<br>ggguacaaucacuucugguuggaccuuuggugcaggug | CoV<br>CoV-2 |
| **23** | **24267/24268** | auggcauauagguucaauggcauugga<br>auggcuuauagguuuaaugguauugga | CoV<br>CoV-2 |
| **24** | **25197/25203** | auggccuugguauguuuggcucggcuucauugcugga<br>auggccaugguacauuuggcuagguuuuauagcuggc | CoV<br>CoV-2 |
| **25** | **28346** | ugacaauaaccagaauggaggacgcaauggggc<br>uggcaguaaccagaauggagaacgcaguggggc | CoV<br>CoV-2 |
| **26** | **28612** | uaggaacuggcccagaagcuucacuucccuacggc<br>uaggaacugggccagaagcuggacuucccuauggu | CoV<br>CoV-2 |
| **27** | **28620** | uggcccagaagcuucacuucccuacggcgcuaacaaagaaggc<br>ugggccagaagcuggacuucccuauggugcuaacaaagacggc | CoV<br>CoV-2 |
| **28** | **28631** | uucacuucccuacggcgcuaacaaagaaggcaucguaugggu<br>uggacuucccuauggugcuaacaaagacggcaucauaugggu | CoV<br>CoV-2 |
| **29** | **28642** | acggcgcuaacaaagaaggcaucguaugguugcaacugagggag<br>augguggcuaacaaagacggcaucauaugguugcaacugagggag | CoV<br>CoV-2 |
| **30** | **28781** | aggcuucuacgcagagggaagcagaggcggc<br>aggcuucuacgcagaagggagcagaggcggc | CoV<br>CoV-2 |
| **31** | **28903** | uggcuagcggagguggu<br>uggcuggcaauggcggu | CoV<br>CoV-2 |
| **32** | **29123** | aggaaauuucggggaccaaga<br>aggaaauuuuggggaccagga | CoV<br>CoV-2 |
| **33** | **29123 el** | aggaaauuucggggaccaagaccuaaucagacaaggaa<br>aggaaauuuuggggaccaggaacuaaucagacaaggaa | CoV<br>CoV-2 |
| **34** | **29234** | uggcauggaagucacaccuucgggaacauggc<br>uggcauggaagucacaccuucgggaacguggu | CoV<br>CoV-2 |
| **35** | **29239** | uggaagucacaccuucgggaacauggcugacuuaucaugga | CoV |

| | | | |
|---|---|---|---|
| | | uggaagucacaccuucgggaacguggguugaccuacacaggu | CoV-2 |
| **36** | **29255** | cgggaacauggcugacuuaucauggagccauuaaauugga<br>cgggaacgugguugaccuacacaggugccaucaaauugga | CoV<br>CoV-2 |

GG-and trinucleotides with GG are indicated by red color. Mismatches are underlined. The start codon AUG in lines 1 and 2 is shown in capital letters and bold. In the last column, PQSs without four GG-repeats are indicated by red.

**Table S5**

Mutational analysis of the SARS-CoV-2 region containing PQSs 509, 644 and 653 (%).

| | **Country** | **China** | **India** | **Ukraine** | **UK** | **USA** |
|---|---|---|---|---|---|---|
| **Year** | **Mutation** | **Numbers of genomes** | | | | |
| **2022** | | **3851** | **4584** | **460** | **8721** | **9059** |
| | **no** | 7 | 11 | 28 | 0 | 0.1 |
| | **U271G** | 87 | 71 | 64 | 80 | 70 |
| | **A6G+U271G** | 0,8 | 5 | 0,6 | 7 | 3 |
| | **A6G+G111A+U271G** | 0 | 3 | 0 | 0.3 | 0.3 |
| | **U109-U124 del** | 0.5 | 0.4 | 0.6 | 6 | 3 |
| | **A287-U295 del** | 1 | 0.2 | 3 | 1 | 12 |

| | **Country** | **China** | **India** | **Ukraine** | **UK** | **USA** |
|---|---|---|---|---|---|---|
| **Year** | **Mutation** | **Numbers of genomes** | | | | |
| **2023** | | **4253** | **1178** | **2321** | **8620** | **8357** |
| | **U271G** | 64 | 7 | 20 | 44 | 7 |
| | **A6G+U271G** | 27 | 81 | 66 | 39 | 76 |
| | **A6G+G8U+U271G** | 0 | 3 | 0 | 0 | 0 |
| | **A6G+A61G+U271G** | 2 | 0.1 | 0.3 | 0 | 0.5 |
| | **U109-U124 del** | 0.4 | 0.1 | 1 | 2 | 3 |

| | **Country** | **China** | **India** | **Ukraine** | **UK** | **USA** |
|---|---|---|---|---|---|---|
| **Year** | **Mutation** | **Numbers of genomes** | | | | |
| **2024** | | **2257** | **1593** | **827** | **3415** | **1385** |
| | **U271G** | 73 | 81 | 74 | 86 | 77 |
| | **A6G+U271G** | 4 | 5 | 13 | 6 | 6 |
| | **A6G+C142U+U271G** | 17 | 0.1 | 0 | 0.1 | 0.4 |
| | **C76U+U271G** | 0.1 | 0.1 | 2 | 0.03 | 0 |
| | **U109-U124 del** | 0.1 | 2 | 3 | 0 | 1 |
| | **A287-U295 del** | 0.2 | 1.3 | 2 | 0.2 | 1 |

Only mutations occurring with the frequency ≥2% (in at least one of the countries) are presented in the Table. Mutations that occur with frequencies of ≥2%, ≥15% and ≥50% are indicated by yellow, blue and green color, respectively. Mutations in PQSs are indicated by red.

Mutation search

ucuuaaagauggcacuuguggcuuaguagaaguugaaaaaggcguuuugccucaacuugaacagcccuauguguucaucaaacguuc
ggaugcucgaacugcaccucau**ggu**cauguuau**ggu**ugagcu**ggu**agcagaacucgaaggcauucaguac**ggu**cguagguggugagac
acuugguguccuugucccucaugugggcgaaauaccaguggcuuaccgcaagguucuucuucguaagaac**gg**uaauaaa**gg**agcu**gg**
u**gg**ccauag**U**uac**gg**cgccgaucuaaagucauuugacuuaggcgacgagcuuggcacugauccuuaugaagauuu

GG-and GGU-repeats are indicated by red. U271 is indicated by blue. Deletion regions are indicated by yellow.

**Table S6A**

Mutational analysis of the SARS-CoV-2 region containing PQSs 1463 and 1558 (%).

| | Country | UK | USA |
|---|---|---|---|
| **Year** | **Mutation** | **Numbers of genomes** | |
| | | **8304** | **8592** |
| **2022** | **C19U** | 0.04 | 2 |
| | **C179U** | 0.02 | 4 |
| | **C261U** | 27 | 5 |

| | Country | UK | USA |
|---|---|---|---|
| **Year** | **Mutation** | **Numbers of genomes** | |
| | | **8650** | **8697** |
| **2023** | **C183U** | 0.5 | 2 |

Only mutations occurring with the frequency ≥2% (in at least one of the countries) are presented in the Table. Mutations that occur with the frequencies of ≥2% and ≥15% are indicated by yellow and blue respectively.

Mutations search

Auuuauuguccagcaugucacaauucagaaguaggaccugagcauagucuugccgaauaccauaaugaaucuggcuugaaaccauu
cuucguaag**gg**u**gg**ucgcacuauugccuuu**gg**a**gg**cuguguguucucuuauguugguugccauaacaagugugccuauugguucca
cgugcuagcgcuaacaua**ggu**uguaaccauaca**ggu**guuguuggagaa**ggu**uccgaa**ggu**cuuaaugacaaccuucuugaaauacuc
caaaaagagaaagucaacaucaauauuguuggugacuuuaaacuuaaugaagagaucgccauuauuuggcaucuuuuucugcuucc
acaagugcuuuuguggaaacugugaaagguuuggauuauaaag

GG-repeats are indicated by red. GGU-repeats are indicated by pink. The most frequent mutations are indicated by color.

**Table S6B**

Mutational analysis of the SARS-CoV-2 region containing PQS 1805 (%).

| | Country | UK | USA |
|---|---|---|---|
| **Year** | **Mutation** | **Numbers of genomes** | |
| **2023** | | 8734 | **8116** |
| | **C18A** | 1 | 4 |
| | **G76A** | 2 | 1 |
| | **C145U** | 2 | 6 |
| | **C229U** | 2 | 1 |

Only mutations occurring with the frequency ≥2% (in at least one of the countries) are presented in the Table. Mutations that occur with the frequencies of ≥2% are indicated by yellow. Mutations in PQSs are indicated by red.

Mutation search

Uuggauuauaaagcauucaaacaaauuguugaauccugugguaauuuuaaaguuacaaaa**gga**aaagcuaaaaaa**ggu**gccu**gga**au
auu**ggu**gaacagaaaucaauacugaguccucuuuaugcauuugcaucagaggcugcucguguuguacgaucaauuuucucccgcacu
cuugaaacugcu**c**aaaauucugugcguguuuuacagaaggccgcuauaacaauacuagauggaauuucacaguauucacugagacuc
auugaugcuaugauguucacaucugauuuggcuacuaacaaucuaguuguaauggccuacauua

GGA and GGU-repeats are indicated by pink. The most frequent mutations are indicated by color.

## Table S7

Mutational search in SARS-CoV-2 region containing PQS 3467 (%).

| | Country | China | India | Ukraine | UK | USA |
|---|---|---|---|---|---|---|
| **Year** | **Mutation** | **Numbers of genomes** | | | | |
| **2023** | | **4246** | **1108** | **2663** | **8725** | **8290** |
| | **no** | 90 | 89 | 87 | 46 | 85 |
| | **G62U** | 0,2 | - | 0,2 | 16 | 0,4 |
| | **C427U** | 1 | 0,3 | 4 | 0,3 | 1 |
| | **U433C** | 2 | - | - | - | 0,6 |
| | **G62U+U196C** | 0,5 | 5 | 0,1 | 26 | 4 |
| | **C418U +C427U** | - | - | 2 | - | 0,01 |

| | Country | China | India | Ukraine | UK | USA |
|---|---|---|---|---|---|---|
| **Year** | **Mutation** | **Numbers of genomes** | | | | |
| **2024** | | **2295** | **1596** | **931** | **3514** | **1558** |
| | **no** | 19 | 6 | 14 | 7 | 8 |
| | **G62U** | 1 | - | 0,9 | 3 | 0,5 |
| | **G62U+U196C** | 73.0 | 82 | 79 | 68 | 76 |
| | **A48d+G62U+U196C** | - | 3 | - | - | - |
| | **U43d+A48d+G62U+U196C** | - | 2 | - | - | - |

Only mutations occurring with a frequency ≥2% (in at least one of the countries) are presented in the Table. Mutations that occur with the frequencies of ≥2%, ≥15% and ≥50% are indicated by yellow, blue and green colours, respectively. d – deletion.

Mutation search

ugacaauguauacauuaaaaaugcagacauuguggaagaagcuaaaaagguaaaaccaacaguggguuguuaaugcagccaauguuuaccuuaa

acau**gg**a**gg**a**gg**uguugca**gg**agccuuaaauaaggcuacuaacaaugccaugcaaguugaaucugaugauuacauagcuacuaauggaccacuuaa

aguggguggguaguuguguuuuaagcggacacaaucuugcuaaacacugucuucauguugucggcccaaauguuaacaaaggugaagacauucaacu

ucuuaagagugcuuaugaaaauuuuaaucagcacgaaguucuacuugcaccauuauuaucagcugguauuuuuggugcugacccuauacauucuuu

aagaguuuguguagauacuguucgcacaaa**uguc**uacuuagcugucuuugauaaaaau

GG-repeats are indicated by red. The most frequent mutations are indicated by colour.

**Table S8A**

Mutational search in SARS-CoV-2 gRNA region corresponding to SUD subdomain in nsp3 protein (%).

| | **Country** | **China** | **India** | **UA** | **UK** | **USA** |
|---|---|---|---|---|---|---|
| **Year** | **Mutation** | **Numbers of genomes** | | | | |
| **2023** | | **4213** | **708** | **2661** | **9517** | **9067** |
| **SUD 1** | **G342A** | - | - | - | - | 6 |
| | **G380A** | 91 | 74 | 75 | 87 | 74 |
| | **C123U+G380A** | 1 | 0.3 | 4 | - | 0.3 |
| | **G380A+C430U** | 1 | 23 | 11 | 4 | 7 |

| | **Country** | **China** | **India** | **UA** | **UK** | **USA** |
|---|---|---|---|---|---|---|
| **Year** | **Mutation** | **Numbers of genomes** | | | | |
| **2023** | | **4233** | **708** | **2661** | **9515** | **9075** |
| **SUD 2** | **C34U** | 97 | 97 | 96 | 97 | 96 |
| | **C34U+C169U** | 0.2 | 2 | - | 0.3 | 0.2 |

| | **Country** | **China** | **India** | **UA** | **UK** | **USA** |
|---|---|---|---|---|---|---|
| **Year** | **Mutation** | **Numbers of genomes** | | | | |
| **2023** | | **4229** | **680** | **2657** | **9438** | **9049** |
| | **no** | 88 | 97 | 89 | 95 | 91 |
| **SUD 3** | **C104U** | 4 | - | - | - | - |
| | **C117U** | 3 | 0.3 | 5 | 0.5 | 2 |

Only mutations occurring with the frequency ≥2% (in at least one of the countries) are presented in the Table. Mutations that occur with frequencies of ≥2%, ≥15% and ≥50% are indicated by yellow, blue and green color, respectively. Mutations in PQSs are indicated by red.

Mutation search.

**SUD 1** (PQSs 4127, 4143, 4161, 4256, beginning of 4262)

aaaucucuaugacaaacuuguuucaagcuuuuuggaaaugaagagugaaaagcaaguugaacaaaagaucgcugagauuccuaaagaggaaguuaagccauuuauaacu
gaaaguaaaccuucaguugaacagagaaaacaagaugauaagaaaaucaaagcuuguguugaagaaguuacaacaacucuggaagaaacuaaguuccucacagaaaacu
uguuacuuuauauugacauuaauggcaaucuucauccagauucugccacucuuguuagugacauugacaucacuuucuuaaagaaagaugcuccauauauagugggugа
uguuguucaagagggugυuuuaacugcuguggu uauaccuacuaaaa**a**ggcuggu ggcacuacugaaaugcuagcgaaagcuuugagaaaagugccaacagacaauuau
auaaccacuuacccgggucagg guuuaaaugguuacacuguagagga

**SUD 2** (the end of PQS 4162)

ggcaaagacagugcuuaaaaaguguaaaagugccuuuuacauucuaccaucuau**u**aucucuaaugagaagcaagaaauucuuggaacuguuucuuggaauuugcgagaa
augcuugcacaugcagaagaaacacgcaaauuaaugccugucugugugg aaacuaaagccauaguuucaacua

**SUD 3** (PQSs 4485/4487, 4616)

Uacagcguaaauauaagggguauuaaaauacaagagggugugguugauuaugguugcuagauuuuacuuuuacaccaguaaaacaacuguagcgucacuuaucaacacacu
uaacgaucuaaaugaaacucuuguuacaaugccacuuggcuauguaacacauggcuuaaauuuggaagaagcugcucgguauaugagaucucucaaagugccagcuaca
guuucuguuucuucaccugaugcuguuacagcguauaaugguuaucuuacuucuucuucuaaaacaccugaagaacauuuuauugaaaccaucucacuugcugg

GG-repeats are indicated by red. GGU-repeats are indicated by pink. The most frequent mutations are indicated by color.

**Table S8B**

Mutational search in SARS-CoV-2 gRNA region corresponding to SUD subdomain in nsp3 protein (%).

| | Country | UK | USA |
|---|---|---|---|
| **Year** | **Mutation** | **Numbers of genomes** | |
| | | **3432** | **1836** |
| **2024** | **G106A** | 0.06 | 9 |
| | **G106A+C243U** | 96 | 84 |
| | **G106A+C243U+A286C** | 1.5 | 0.5 |

| | Country | UK | USA |
|---|---|---|---|
| **Year** | **Mutation** | **Numbers of genomes** | |
| | | **7470** | **4334** |
| **2025** | **G106A+C243U** | 56 | 80 |
| | **G106A+U136G+C243U** | 34 | 11 |
| | **G106A+C243U+A286C** | 3 | 3 |

Only mutations occurring with the frequency ≥2% (in at least one of the countries) are presented in the Table. Mutations that occur with frequencies of ≥2%, ≥15% and ≥50% are indicated by yellow, blue and green color, respectively. Mutations in PQSs are indicated by red.

Mutation search.

Agugacauugacaucacuuucuuaaagaaagaugcuccauauauagugggugauguuguucaagagggugguuuuaacugcugugggu
auaccuacuaaaaaggcugguggcacuacugaaaugcuagcgaaagcuuugagaaaagugccaacagacaauuauauaaccacuuac
ccgggucaggguuuaaauggguacacuguagaggaggcaaagacagugcuuaaaaaguguaaaagugccuuuuacauucuaccaucu
auuaucucuaaugagaagcaagaaauucuuggaacuguuucuuggaauuugcgagaaaugcuugcacaugcagaagaaacacgca
346

Trinucleotides with GG-repeats are indicated by pink. The most frequent mutations are indicated by color.

**Table S9**

Mutational search in SARS-CoV-2 gRNA region containing PQS 8687 (%).

**2022**

| Sequence | China | India | Ukraine | UK | USA |
|---|---|---|---|---|---|
| | **3915** | **5403** | **765** | **8205** | **9097** |
| **no** | 83 | 97 | 96 | 94 | 95 |
| **C169U** | 15 | 0,05 | 0,1 | 0,1 | 0,3 |
| **C188U** | 2 | 0,02 | 0 | 0,1 | 0 |

**2023**

| Sequence | China | India | Ukraine | UK | USA |
|---|---|---|---|---|---|
| | **4266** | **1204** | **2857** | **9402** | **8948** |
| **no** | 55 | 96 | 95 | 93 | 91 |
| **C169U** | 42 | 0 | 0,1 | 0,1 | 0,07 |
| **U280C** | 0,02 | 0 | 0,1 | 1 | 2 |

**2024**

| Sequence | China | India | Ukraine | UK | USA |
|---|---|---|---|---|---|
| | **2305** | **1702** | **946** | **3576** | **1609** |
| **no** | 94 | 95 | 93 | 95 | 94 |
| **G121A** | 0,6 | 0,1 | 3 | 0,4 | 0,6 |

Only mutations occurring with the frequency ≥2% (in at least one of the countries) are presented in the Table. Mutations that occur with frequencies of ≥2%, ≥15% and ≥50% are indicated by yellow, blue and green color, respectively.

Mutations search

ugcugcuaaaaagaauaacuuaccuuuuaaguugacaugugcaacuacuagacaaguuguuaauguuguaacaacaaagauagcacu
uaagggugguaaaauuguuaauaauugguugaagcaguuaauuaaaguuacacuuguguuccuuuuuguugcugcuauuuucuauuu
aauaacaccuguucaugucaugucuaaacauacugacuuuucaagugaaaucauaggauacaaggcuauugauggugguguucacucg
ugacauagcaucuacagauacuuguuuugcuaacaaacaugcugauuuugacacaugguuuagccagcg

GG-repeats are indicated by red. The most frequent mutations are indicated by color.

**Table S10**

Mutational search in SARS-CoV-2 gRNA region containing PQSs 10058/10085 and 10254/10260 (%).

| | Country | China | India | UA | UK | USA |
|---|---|---|---|---|---|---|
| **Year** | **Mutation** | **Numbers of genomes** | | | | |
| **2022** | | **3904** | **3966** | **205** | **7839** | **7868** |
| | **no** | 3 | 1 | 0,5 | 0 | 0 |
| | **C19U** | 5 | 12 | 49 | 0,3 | 0,1 |
| | **C19U+C188U** | 91 | 80 | 48 | 93 | 91 |

| | Country | China | India | Ukraine | UK | USA |
|---|---|---|---|---|---|---|
| **Year** | **Mutation** | **Numbers of genomes** | | | | |
| **2023** | | **4254** | **683** | **1603** | **9438** | **6717** |
| | **C19U+ C188U** | 96 | 93 | 89 | 94 | 87 |
| | **C19U+A32G+C188U** | 0 | 0 | 0,1 | 2 | 0,2 |
| | **C19U+C188U+U194C** | 0,7 | 1,5 | 2 | 0,2 | 8 |
| | **C19U+C188U+C218U** | 0,02 | 0 | 3 | 0,1 | 0,06 |

| | Country | China | India | Ukraine | UK | USA |
|---|---|---|---|---|---|---|
| **Year** | **Mutation** | **Numbers of genomes** | | | | |
| **2024** | | **2293** | **1347** | **814** | **3577** | **1530** |
| | **C19U+C188U** | 78 | 93 | 97 | 96 | 88 |
| | **C19U+A32G+C188U** | 18 | 0 | 0 | 0,1 | 0,3 |

Only mutautons occurring wiuh the frequency ≥2% (in at least one of the countries) are presented in uhe Table. Mutations that occur with frequencies of ≥2%, ≥15% and ≥50% are indicated by yellow, blue and green color, respectively. Mutations in PQSs are indicated by red.

Mutation search

uuuaccaaccaccacaaaccucuaucaccucagcuguuuugcagaguggguuuuagaaaaauggcauucccaucugguaaaguugagg guuguauggguacaaguaacuugugguacaacuacacuuaacggucuuuggcuugaugacguaguuuacuguccaagacaugugaucu gcaccucugaagacaugcuuaacccuaauuaugaagauuuacucauucguaagucuaaucauaauuucuugguacaggcugguaaug uucaacucaggguuauuggacauucuaugcaaaauuguguac

GG-repeat and trinucleotides with GG-repeats are indicated by pink. The most frequent mutations are indicated by color.

**Table S11**

Mutational search in SARS-CoV-2 gRNA region containing PQSs 10466-10674, (%).

| | Country | China | India | Ukraine | UK | USA |
|---|---|---|---|---|---|---|
| **Year** | **Mutation** | **Numbers of genomes** | | | | |
| **2022** | | **3919** | **4398** | **205** | **9184** | **8739** |
| | **no** | 3 | 6 | 6 | 0.1 | 0 |
| | **C104A** | 4 | 6 | 38 | 0.02 | 0.1 |
| | **G102A+C104A** | 63 | 84 | 46 | 93 | 92 |
| | **C24U+G102A+C104A** | 0.1 | 0.1 | 0.5 | 0.3 | 2 |
| | **G102A+C104A+U351C** | 27 | 0.04 | 0 | 0 | 0.01 |

| | Country | China | India | Ukraine | UK | USA |
|---|---|---|---|---|---|---|
| **Year** | **Mutation** | **Numbers of genomes** | | | | |
| **2023** | | **4268** | **700** | **1513** | **9506** | **7745** |
| | **G102A+C104A** | 94 | 96 | 91 | 92 | 93 |
| | **G102A+C104A+U411C+A412U** | 0 | 0 | 2 | 1.4 | 0.4 |

| | Country | China | India | Ukraine | UK | USA |
|---|---|---|---|---|---|---|
| **Year** | **Mutation** | **Numbers of genomes** | | | | |
| **2024** | | **2317** | **1517** | **821** | **3577** | **1602** |
| | **G102A+C104A** | 95 | 87 | 93 | 96 | 92 |
| | **G102A+C104A+A412U** | 0 | 2 | 0 | 0 | 0 |

Only mutations occurring wiuh the frequency ≥2% (in at least one of the countries) are presented in the Table. Mutations that occur with the frequencies of ≥2%, ≥15% and ≥50% are indicated by yellow, blue and green color, respectively.

## Mutations search

acaccuaaguauaaguuuguucgcauucaaccaggacagacuuuuucaguguuagcuuguuacaaugguucaccaucugguguuuac caaugugcuaugaggccaauuucacuauuaagguucauuccuuaaugguucaugugguaguguugguuuuaacauagauuaugac ugugucucuuuuuguuacaugcaccauauggaauuaccaacuggaguucaugcuggcacagacuuagaagguaacuuuuauggaccu uuuguugacaggcaaacagcacaagcagcugguacggacacaacuauuacaguuaauguuuuagcuugguuguacgcugcuguuaua aauggagacaggugguuucucaaucgauuuaccacaacucuuaaugacuuuaaccuuguggcua

GG-repeats are indicated by red. Trinucleotides with GG-repeats are indicated by pink. The most frequent mutations are indicated by color.

**Table S12**

GRSs in SARS-CoV-2 genome.

| Gene | Length, nts | PQS number | GRS number | GRS density, nts |
|---|---|---|---|---|
| 5 UTR | 265 | 3 | 1 | 265 |
| Nsp1 | 540 | 13 | 3 | 180 |
| Nsp2 | 1914 | 11 | 4 | 478 |
| Nsp3 | 5835 | 17 | 7 | 834 |
| Nsp4 | 1500 | 1 | 1 | 1500 |
| Nsp5 | 918 | 19 | 4 | 229 |
| Nsp6 | 870 | 2 | 1 | 870 |
| Nsp9 | 339 | 2 | 1 | 339 |
| Nsp10 | 417 | 7 | 2 | 208 |
| Nsp12 | 2796 | 6 | 3 | 932 |
| Nsp14 | 1581 | 1 | 1 | 1581 |
| Nsp16 | 894 | 6 | 1 | 894 |
| S | 3819 | 18 | 5 | 764 |
| ORF3a | 825 | 1 | 1 | 825 |
| M | 669 | 3 | 1 | 669 |
| N | 1257 | 22 | 6 | 209 |

The highest amount of PQSs is indicated by blue. The highest GRS density is indicated by yellow. Genes with highest amount of PQSs and highest GRS density are indicated by green.

## Table S13

Mutational search in SARS-CoV-2 gRNA region containing PQS 13385 (%).

| | **Country** | **India** | **Ukraine** | **UK** | **USA** |
|---|---|---|---|---|---|
| **Year** | **Mutation** | **Numbers of genomes** | | | |
| **2022** | | **5421** | **787** | **8756** | **9639** |
| | **no** | 91 | 81 | 94 | 95 |
| | **U44C** | 5 | 15 | 0.01 | 0.05 |

| | **Country** | **India** | **Ukraine** | **UK** | **USA** |
|---|---|---|---|---|---|
| **Year** | **Mutation** | **Numbers of genomes** | | | |
| **2023** | | **1218** | **2876** | **8828** | **7733** |
| | **no** | 90 | 92 | 43 | 83 |
| | **U188C** | 5 | 0.2 | 46 | 3 |
| | **U260C** | 0 | 2 | 0.01 | 0 |
| | **G276A** | 0 | 0 | 0 | 6 |
| | **U188C+G 276A** | 0 | 0 | 0 | 2 |

| **Year** | **Country** | **UK** | **USA** |
|---|---|---|---|
| **2024** | **Mutation** | **Numbers of genomes** | |
| | | **3428** | **1311** |
| | **no** | 3 | 19 |
| | **U188C** | 92 | 73 |

| **Year** | **Country** | **UK** | **USA** |
|---|---|---|---|
| **2025** | **Mutation** | **Numbers of genomes** | |
| | | **4969** | **3976** |
| | **no** | 11 | (0) |
| | **U188C** | 75 | 87 |
| | **C146U+U188C** | 1 | 2 |
| | **U188C+G276A** | 0.4 | 3 |

Only mutations occurring with the frequency ≥2% (in at least one of the countries) are presented in the Table. Mutations that occur with frequencies of ≥2%, ≥15% and ≥50% are indicated by yellow, blue and green color, respectively.

## Mutations search

agauguuguguacacacacugguacuggucaggcaauaacaguuacaccggaagccaauauggaucaagaauccuuuggugugcaucguguuguc
uguacugccguugccacauagaucauccaaauccuaaaggauuuugugacuuaaaagguaaguauguacaaauaccuacaacuugugcuaaugacc
cugugguuuuacacuuaaaaacacagucuguaccgucugc**ggu**augu**gga**aa**ggu**uau**gg**cuguaguugugaucaacuccgcgaacccaugcuuc
agucagcugaugcacaaucguuuuuaaacggguuugcgguguaagugcagcccgucuuacaccgugcggcacaggcacuaguacugaugucguaua
cagggcuuuugacaucuacaaugauaaaguagc

GG-repeats are indicated by red. The most frequent mutations are indicated by color.

**Table S14A**

Mutational search in SARS-CoV-2 gRNA 120 nts region containing PQS 22316 and Ψ-site 22322 (%).

| | Country | India | Ukraine | UK | USA |
|---|---|---|---|---|---|
| **Year** | **Mutation** | **Numbers of genomes** | | | |
| **2020** | | **7692** | **106** | **8285** | **3095** |
| | **no** | 97 | 99 | 97 | 95 |
| | **U24C** | 0.04 | 0 | 0 | 2 |

| **Year** | **Country** | **India** | **Ukraine** | **UK** | **USA** |
|---|---|---|---|---|---|
| **2021** | **Mutation** | **Numbers of genomes** | | | |
| | | **4214** | **569** | **6675** | **6963** |
| | **no** | 96 | 93 | 99 | 96 |
| | **C48U** | 0.1 | 2 | 0 | 0.1 |

| **Year** | **Country** | **India** | **Ukraine** | **UK** | **USA** |
|---|---|---|---|---|---|
| **2022** | **Mutation** | **Numbers of genomes** | | | |
| | | **3594** | **680** | **8204** | **7058** |
| | **no** | 66 | 95 | 62 | 94 |
| | **U41A** | 4 | 0 | 0 | 0 |
| | **G54U** | 3 | 0.6 | 4 | 1 |
| | **A57G** | 2 | 0 | 3 | 0,1 |
| | **G68A** | 22 | 1 | 25 | 2 |

| **Year** | **Country** | **India** | **Ukraine** | **UK** | **USA** |
|---|---|---|---|---|---|
| **2023** | **Mutation** | **Numbers of genomes** | | | |
| | | **1049** | **1586** | **8851** | **7336** |
| | **G54U** | 60 | 54 | 38 | 70 |
| | **A57G** | 19 | 15 | 4 | 7 |
| | **G68A** | 0.6 | 7 | 0.7 | 2 |
| | **C32A+C90A** | 15 | 0 | 52 | 6 |

Only mutations occurring wiuh the frequency ≥2% (in at least one of the countries) are presented in the Table. Mutations that occur with the frequencies of ≥2%, ≥15% and ≥50% are indicated by yellow, blue and green color, respectively. Mutations in PQSs are indicated by red.

**Mutations search**

caucacuagguuucaaacuuuacuugcuuuacauagaaguuauuugacucccu**gg**ugauu**c**uucuucaggu**ugg**acagcu**gg**ugcugc agcuuauuaugug**ggu**uaucuucaaccua**gg**ac

GG-repeats and trinucleotides with GG-repeats are indicated by red. Ψ-site is indicated by blue.

**Table S14B**

Additional search in the elongated region.

| Year | Country | UK | USA |
|---|---|---|---|
| 2024 | Mutation | Numbers of genomes | |
| | | 3428 | 1553 |
| | U87A+G204U | 2 | 6 |
| | U87G+C95U+C182A+C240A | (0-4) | 8 |
| | 81-83 del+U87G+C95U+C182A | 0 | 5 |
| | 81-83 del+U87G+C95U+C182A+C240A | 83 | (36)-62 |

| Year | Country | UK | USA |
|---|---|---|---|
| 2025 | Mutation | Numbers of genomes | |
| | | 7413 | 4263 |
| | 81-83 del+U87G+C95U+C182A+C240A | 31 | 46 |
| | G19U+81-83 del+U87G+C95U+C182A+C240A | 37 | 18 |
| | C7A+G19U+81-83 del+U87G+C95U+C182A+C240A | 16 | 25 |

Only mutations occurring with the frequency ≥2% (in at least one of the countries) are presented in the Table. Mutations that occur with the frequencies of ≥2%, ≥15% and ≥50% are indicated by yellow, blue and green color, respectively. Mutations in PQSs are indicated by red.

## Mutations search

uaauuucaaaaaucuuagggaauuuguguuuaagaauauugaugguuauuuuaaaauauauucuaagcacacgccuauuaauuuagu
gcgugaucucccucaggguuuuucggcuuuagaaccauugguagauuugccaauagguauuaacaucacuagguuucaaacuuuacu
ugcuuuacauagaaguuauuugacuccugg**u**gauucuucuucagguuggacagcugguugcugcagcuuauuaugugg**u**uaucuuca
accuaggacuuuucuauuaaaauauaaugaaaauggaaccauua 305

GGU-repeats are indicated by pink. The most frequent mutations are indicated by color. Ψ-site is indicated by blue.

**Table S15**

Mutational search in SARS-CoV-2 gRNA region containing PQSs 24200/24215 and 24267/24268 (%).

| | **Country** | **India** | **Ukraine** | **UK** | **USA** |
|---|---|---|---|---|---|
| **Year** | **Mutation** | **Numbers of genomes** | | | |
| **2020** | | **8849** | **106** | **9042** | **3274** |
| | **no** | 97 | 85 | 98 | 97 |
| | **C1U** | 0.1 | 13 | 0 | 0.03 |

| | **Country** | **India** | **Ukraine** | **UK** | **USA** |
|---|---|---|---|---|---|
| **Year** | **Mutation** | **Numbers of genomes** | | | |
| **2021** | | **2201** | **617** | **8745** | **9213** |
| | **no** | 98 | 93 | 4 | 43 |
| | **C35A** | 0 | 1 | 94 | 37 |
| | **C113U** | 0 | 0 | 0.01 | 17 |

| | **Country** | **India** | **Ukraine** | **UK** | **USA** |
|---|---|---|---|---|---|
| **Year** | **Mutation** | **Numbers of genomes** | | | |
| **2022** | | **6518** | **815** | **9205** | **9299** |
| | **no** | 88 | 84 | 96 | 98 |
| | **C35A** | 8 | 15 | 0.02 | 0.04 |
| | **C115U** | 0 | 0.1 | 2 | 0.3 |

| | **Country** | **India** | **Ukraine** | **UK** | **USA** |
|---|---|---|---|---|---|
| **Year** | **Mutation** | **Numbers of genomes** | | | |
| **2023** | | **6543** | **2965** | **9371** | **4059** |
| | **no** | 93 | 97 | 97 | 97 |

Only mutations occurring wiuh the frequency ≥2% (in at least one of the countries) are presented in the Table. Mutations that occur with the frequencies of ≥2%, ≥15% and ≥50% are indicated by yellow, blue and green color, respectively.

**Mutations search**

cugcuagagaccucauuugugcacaaaaguuuaacggccuuacuguuuugccaccuuugcucacagaugaaaugauugcucaauaca
cuucugcacuguuagcgg**gg**uacaaucacuucu**gg**uu**gg**accuuu**gg**ugca**gg**ugcugcauuacaaauaccauuugcuaugcaaau**gg**
cuuaua**gg**uuuaau**gg**uauu**gg**ag 198

GG repeats are indicated by red. Frequenu base changes are indicaued by green.

**Table S16**

Mutational search in SARS-CoV-2 gRNA region containing PQSs 25197/25203 (%).

| Year | Country | UK | USA |
|---|---|---|---|
| **2024** | **Mutation** | **Numbers of genomes** | |
| | | **3407** | **1842** |
| | **C21U** | 89 | 86 |
| | **C230U** | 3 | 7 |

| Year | Country | UK | USA |
|---|---|---|---|
| **2025** | **Mutation** | **Numbers of genomes** | |
| | | **6713** | **4343** |
| | **C21U** | 87 | 93 |

Only mutations occurring with the frequency ≥2% (in at least one of the countries) are presented in the Table. Mutations that occur with the frequencies of ≥2 and ≥50% are indicated by yellow and green color, respectively.

**Mutations search**

Tatataaaat**gg**ccat**gg**tacattt**gg**cta**gg**ttttatagct**gg**cttgattgccatagtaatggtgacaattatgctttgctgtatgaccagttgc
tgtagttgtctcaagggctgttgttcttgtggatcctgctgcaaatttgatgaagacgactctgagccagtgctcaaaggagtcaaattacattac
acataaacgaacttatggatttgtttatgagaatcttcacaa

GG repeats are indicated by red. Frequent base changes are indicated by blue.

**Table S17**

Mutational search in SARS-CoV-2 gRNA region containing PQS 28346 (%).

| Year | Country | Ukraine | UK | USA |
|---|---|---|---|---|
| 2022 | Mutation | Numbers of genomes | | |
| | | 321 | 8614 | 9082 |
| | Deletion, nts 19-27 | 93 | 96 | 86 |
| | G17U+G18U | 4 | 1 | 10 |

| Year | Country | Ukraine | UK | USA |
|---|---|---|---|---|
| 2023 | Mutation | Numbers of genomes | | |
| | | 2452 | 6757 | 9624 |
| | Deletion, nts 19-27 | 89 | 96 | 97 |
| | G18C | 7 | 1 | 0.03 |

| Year | Country | UK | USA |
|---|---|---|---|
| 2024 | Mutation | Numbers of genomes | |
| | | 990 | 1646 |
| | No | 0 | 9 |
| | Deletion, nts 19-27 | 96 | 79 |

| Year | Country | UK | USA |
|---|---|---|---|
| 2025 | Mutation | Numbers of genomes | |
| | | 6011 | 4074 |
| | Deletion, nts 19-27 | 82 | 88 |
| | G79A+del | 1 | 4 |
| | C88U+del | 12 | 4 |

Only mutations occurring wiuh the frequency ≥2% (in at least one of the countries) are presented in the Table. Mutations that occur with the frequencies of ≥2% and ≥50% are indicated by yellow and green color, respectively.

## Mutations search

u**gg**caguaaccagaau**gg**agaacgcagu**gggg**cgcgaucaaaacaacgucggccccaagguuuacccaauaa**u**acugcgucuugguu ca

GG-repeats are indicated by red. The most frequent mutations are indicated by color.

**Table S18**

Mutational search in SARS-CoV-2 gRNA region containing PQSs 28613 and 28781 (%).

| Year | Country | UK | USA |
|---|---|---|---|
| **2022** | **Mutation** | **Numbers of genomes** | |
| | | **9190** | **9356** |
| | **G247U** | 44 | 19 |
| | **C290U** | 0.4 | 11 |

| Year | Country | UK | USA |
|---|---|---|---|
| **2023** | **Mutation** | **Numbers of genomes** | |
| | | **9351** | **9401** |
| | **A13G** | 6 | 15 |

Only mutations occurring wiuh the frequency ≥2% (in at least one of the countries) are presented in the Table. Mutations that occur with the frequencies of ≥2% and ≥15% are indicated by yellow and blue color, respectively.

## Mutations search

cgcucucacucaacauggcaaggaagaccuuaaauucccucgaggacaaggcguuccaauuaacaccaauagcaguccagaugacca
aauuggcuacuaccgaagagcuaccagacgaauucgugguggugacgguaaaaugaaagaucucaguccaagaugguauuucuacua
ccua**gg**aac**ugg**gccagaagcu**gg**acuucccua**ugg**ugcuaacaaagac**gg**caucauauggguugcaacugagggagccuugaauac
accaaaagaucacauuggcacccgcaauccugcuaacaaugcugcaaucgugcuacaacuucc**u**caaggaacaacauugccaaaa**gg**
cuucuacgcagaa**gg**gagcaga**gg**c**gg**cagucaagccucuucucguuccucaucacguagucgca

GG-repeats are indicated by red. Trinucleotides with GG-repeats are indicated by pink. The most frequent mutations are indicated by color.

**Table S19**

Mutational search in SARS-CoV-2 gRNA region containing PQS 28903 (%).

| Year | Country | UK | USA |
|---|---|---|---|
| 2024 | Mutation | Numbers of genomes | |
| | | 3412 | 1876 |
| | G31A+G32A+G33C | 5 | 8 |
| | G31A+G32A+G33C+108A | 89 | 79 |
| | G31A+G32A+G33C+G34C+C108A | 2 | 7 |

| Year | Country | UK | USA |
|---|---|---|---|
| 2025 | Mutation | Numbers of genomes | |
| | | 7426 | 4163 |
| | G31A+G32A+G33C | 11 | **4** |
| | G31A+G32A+G33C+108A | 60 | 64 |
| | G31A+G32A+G33C+G34C+C108A | 19 | 26 |
| | A27U+G28C+G31A+G32A+G33C+C108A | 5 | 0.7 |

Only mutations occurring with the frequency ≥2% (in at least one of the countries) are presented in the Table. Mutations that occur with the frequencies of ≥2%, ≥15% and ≥50% are indicated by yellow, blue and green color, respectively.

**Mutations search**

guucaagaaauucaacuccaggcagcaguaggggaacuucuccugcuagaauggcuggcaauggcggugaugcugcucuugcuuugc ugcugcuugacagauugaaccagcuugaga

GG-repeats are indicated by red. The most frequent mutations are indicated by color.

**Table S20**

Mutational search in SARS-CoV-2 gRNA region containing PQS 29123 and 29234 (%).

| Year | Country | UK | USA |
|---|---|---|---|
| 2022 | Mutation | Numbers of genomes | |
| | | 9102 | 9191 |
| | C350U | 2 | 0.05 |
| | | | |

| Year | Country | UK | USA |
|---|---|---|---|
| 2023 | Mutation | Numbers of genomes | |
| | | 9651 | 8979 |
| | C383U | 6 | 15 |

Only mutations occurring with the frequency ≥2% (in at least one of the countries) are presented in the Table. Mutations that occur with the frequencies of ≥2% and ≥15% are indicated by yellow and blue respectively.

## Mutations search

aaacugucacuaagaaaucugcugcugaggcuucuaagaagccucggcaaaaacguacugccacuaaagcauacaauguaacacaag
cuuucggcagacg**ugg**uccagaacaaaccca**agg**aaauuu**ugggg**acc**agg**aacuaaucagacaa**gga**acugauuacaaacauuggc
cgcaaauugcacaauuugcccccagcgcuucagcguucuuc**gg**aaugucgcgcauu**gg**ca**ugg**aagucacaccuucgggaacguggu
ugaccuacaca**ggu**gccaucaaauu**gga**ugacaaagauccaaauuucaaagaucaagucauuuugcugaauaagcauauugacgcau
acaaaacauucccaccaacagagccuaaaaaggacaaaaagaagaaggcugaugaaacucaagccuuaccgcagagacagaagaaac
agcaaacugugacucuucuuccugcugcagauuuggaugauuucuccaaacaauugcaacaa

Trinucleotides with GG-repeats are indicated by pink. The most frequent mutations are indicated by color.

**Table S21**

The most frequent mutations in SARS-CoV-2 gRNA regions containing PQSs.

| PQSs in region under study | Mutations | Year | Countries | Mutation frequency (%) | Impact on G4 formation | Impact on G4 location |
|---|---|---|---|---|---|---|
| **236, 353, 359, 370** | C21U<br>C44U<br>C241U | 2024 | China, India, UA,<br>UK, USA | 2-20<br>0-51<br>34-89 | no | no |
| **509, 644, 653** | U271G<br>A6G+U271G<br>A6G+C142U+U271G | 2024 | China, India,UA, UK, USA | 73-86<br>4-13<br>0-17 | yes | yes |
| **1463, 1558, 1805** | C18A<br>C145U | 2023 | UK, USA | 1-4<br>2-6 | no | no |
| **3467** | no<br>G62U+U196C | 2024 | China, India,UA, UK, USA | 6-19<br>68-82 | no | no |
| **4127, 4143, 4161, 4256, 4262** | G380A<br>G380A+C430U | 2023 | China, India,UA, UK, USA | 74-91<br>4-23 | yes for 4127, 4143, 4161 | yes for, 4256, 4262 |
| | G106A+C243U<br>G106A+U136G+C243U | 2025 | UK, USA | 56-80<br>11-34 | | |
| **4485/4487** | no | 2025 | China, India, UA, UK, USA | 88-97 | no | no |
| **8687** | no | 2024 | China, India, UA, UK, USA | 93-95 | nd | nd |
| **10058/10085, 10254/10260** | C19U+C188U<br>C19U+A32G+C188U | 2024 | China, India, UA, UK, USA | 78-97<br>0-18 | no | no |
| **10466/10674** | G102A+C104A | 2024 | China, India, UA, UK, USA | 87-96 | no | yes for 10466 |
| **13385** | U188C | 2025 | UK, USA | 75-87 | no | no |
| **22316** | 81-83 del +U87G+C95U +C182A +C240A<br>G19U+81-83del+U87G+C95U+C182A +C240A<br>C7A+G19U+81-83 del+U87G+C95U+ C182A+C240A | 2025 | UK, USA | 31-46<br>18-37<br>16-25 | base change in the 3rd loop | Small alteration |
| **24200/24215, 24267/24268** | no | 2023 | India, UA, UK, USA | 93-97 | no | no |
| **25197/25203** | C21U | 2025 | UK, USA | 87-93 | base change in the 2nd/3rd loops | no |
| **28346** | Deletion, nts 19-27<br>C88U+del | 2025 | UK, USA | 82-88<br>4-12 | yes | yes |
| **28613/28619** | A13G | 2023 | UK, USA | 6-15 | no | no |
| **28781** | A13G | 2023 | UK, USA | 6-15 | no | no |
| **28903** | G31A+G32A+G33C+108A<br>G31A+G32A+G33C+G34C+C108A | 2025 | UK, USA | 60-64<br>19-26 | no | yes |

Mutations are numerated according to gRNA region in which the search was conducted. G4 destroying mutation is idicated by red.

# Figures

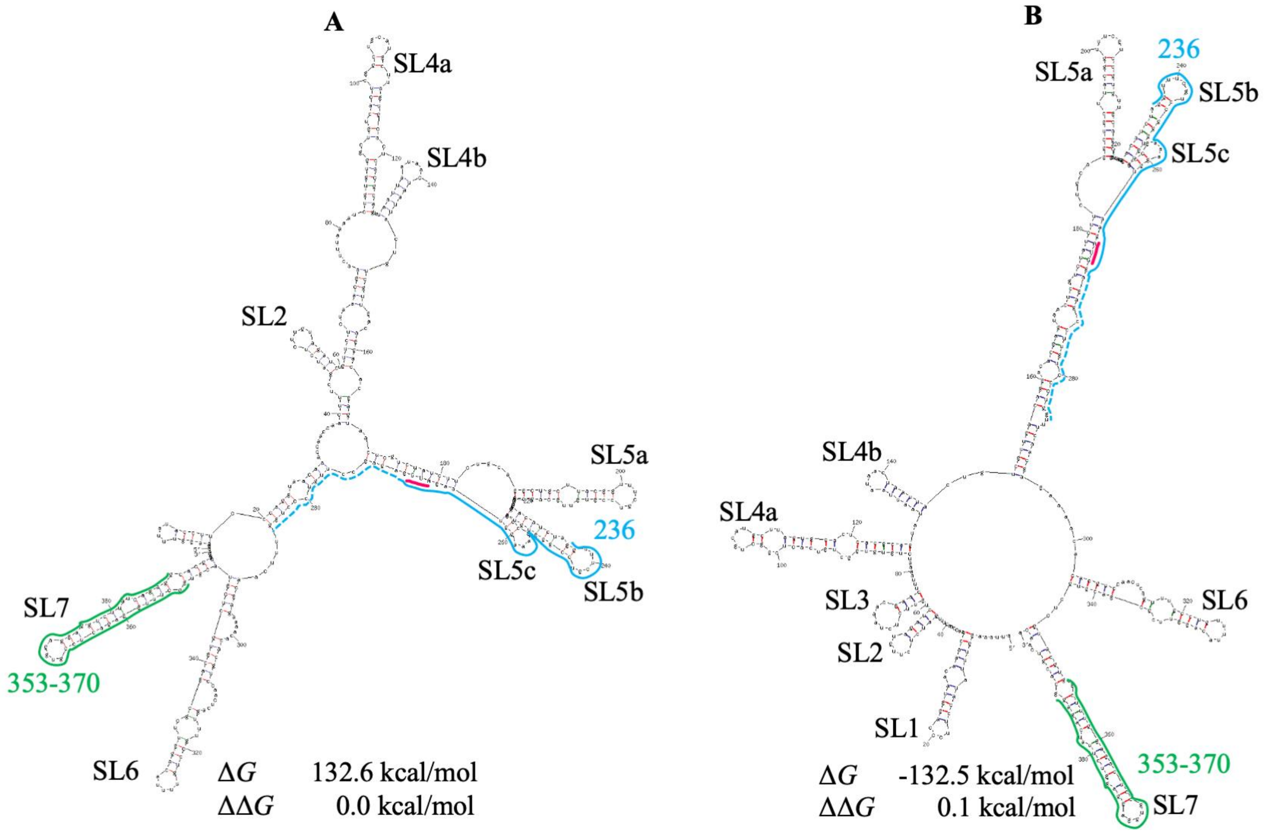


**Figure S1**. Secondary structure prediction of the SARS-CoV-2 5' leader. PQSs 236 and 353-370 are indicated by blue and green lines, respectively. PQS 236 elongation is indicated by a dotted line. Start codon AUG indicated by red. Hairpin numeration is as in [59]. ΔG – change in free energy. ΔΔG – the energy increment of the lowest change in free energy.

236 aggguuucguccgggugugaccgaaagguaagaugga

236el uagguuucguccgggugugaccgaaagguaagauggagagccuugucccugguuucaacgaga

353 uggcuuuggagacuccguggagga

353el guggcuuuggagacuccguggaggaggu

359 uggagacuccguggaggaggu

370 uggaggaggucuuaucagaggc

508 auggucauguuaugguugagcugguagcagaacucgaaggc

509 uggucauguuaugguugagcugguagcagaacucgaaggcauucaguacgguc

529 ugguagcagaacucgaaggcauucaguacggucguaguggu

529el ugguagcagaacucgaaggcauucaguacggucguaguggugagacacuuggug

545 aggcauucaguacggucguaguggugagacacuuggu

644 cgguaauaaaggagcugguggc

653 aggagcugguggccauaguuacggc

653m aggagcugguggccauagGu

659m ugguggccauagGuacggc

1558 uagguuguaaccauacagguguuguuggagaagguuccgaaggu

1559 agguuguaaccauacagguguuguuggagaagguuccgaagguc

1574 agguguuguuggagaagguuccgaaggu

1574a agguguuguuggagaagguuccgaaggu

1784 ugguaauuuuaaaguuacaaaaggaaaagcuaaaaaaggugccuggaa

1804 aaggaaaagcuaaaaaaggugccuggaauauuggu

1805 aggaaaagcuaaaaaaggugccuggaauauuggug

2714 aggcggugcaccaacaaagguuacuuuuggug

2717 cggugcaccaacaaagguuacuuuuggugaugacacugugauagaagugcaagguu

4127 ggggugauguuguucaagaggguguuuuaacugcugugguuauaccuacuaaaaaggcuggug

4143 gaggguguuuuaacugcugugguuauaccuacuaaaaaggcuggu

4161 gugguuauaccuacuaaaaaggcugguggc

4256a gggucaggguuuaaaugguuacacuguagaggag

4262 ggguuuaaaugguuacacuguagaggaggc

4485 aaggguauuaaaauacaagagggugugguugauuauggu

4487 ggguauuaaaauacaagagggugugguugauuauggug

10058 gugguuuuagaaaaauggcauucccaucugguaaaguugaggg

10072 uggcauucccaucugguaaaguugaggguuguauggu

10085 ugguaaaguugaggguuguaugguacaaguaacuugugguа

10097 aggguuguaugguacaaguaacuuguggu acaacuac…

10085 ugguaaaguugaggguuguaugguacaaguaacuuguggua

10097 aggguuguaugguacaaguaacuugugguacaacuacacuuaacgguc

10254 uugguacaggcugguaauguucaacucagggu

10260 caggcugguaauguucaacucaggguuauugga

10548 auggaauuaccaacuggaguucaugcuggcacagacuuagaaggu

10562 uggaguucaugcuggcacagacuuagaagguaacuuuuaugga

10573 cuggcacagacuuagaagguaacuuuuauggaccuuuuguugacaggc

10588 aagguaacuuuuauggaccuuuuguugacaggcaaacagc…

22315 cuggugauucuucuucagguuggacagcuggu

22316 uggugauucuucuucagguuggacagcuggu

22316a uggugauucuucuucagguuggacagcuggug

22315el uggugauucuucuucagguuggacagcuggugcugcagcuuauuaugugggu

22316el uggugauucuucuucagguuggacagcuggugcugcagcuuauuaugugggu

22330 cagguuggacagcuggugcugcagcuuauuaugugggu

22331 agguuggacagcuggugcugcagcuuauuaugugggu

24200a cggguacaaucacuucugguuggaccuuuggugcaggu

24200a cggguacaaucacuucugguuggaccuuuggugcaggug

24214 cugguuggaccuuuggugcaggu

24215 ugguuggaccuuuggugcaggu

24215a ugguuggaccuuuggugcaggu

25196 auggccaugguacauuuggcuaggu

25197 uggccaugguacauuuggcuaggu

25203 ugguacauuuggcuagguuuuauagcuggc

28612 uaggaacugggccagaagcuggacuucccuauggu

28613 uaggaacugggccagaagcuggacuucccuauggu

28619 ugggccagaagcuggacuucccuauggugcuaacaaagacggc

28620 gggccagaagcuggacuucccuauggugcuaacaaagacggc

28631 uggacuucccuauggugcuaacaaagacggcaucauauggguu

28642 auggugcuaacaaagacggcaucauaugggguugcaacugaggg

28643 auggugcuaacaaagacggcaucauaugguugcaacugaggag

29104 gugguccagaacaaacccaaggaaauuuuggggaccagga

29123 aggaaauuuuggggaccagga

29123el aggaaauuuuggggaccaggaacuaaucagacaagga

29132 uggggaccaggaacuaaucagacaagga

29219 cggaaugucgcgcauuggcauggaagucacaccuucggga

29234 uggcauggaagucacaccuucgggaacguggu

29238 cauggaagucacaccuucgggaacgugguugaccuacacaggu

29239 uggaagucacaccuucgggaacgugguugaccuacacaggugc

29254 cgggaacgugguugaccuacacaggugccaucaaauugga

29255 cgggaacgugguugaccuacacaggugccaucaaauuggau

29255a gggaacgugguugaccuacacaggugccaucaaauugga

**Figure S2**. Overlapping PQSs. GG repeats (together with other bases) in PQS stabilized by tetrads or triads are indicated by pink color, those in unstabilized PQSs are indicated by red color. The mutation in PQS 653m is marked with a capital letter.

**A Nsp1**

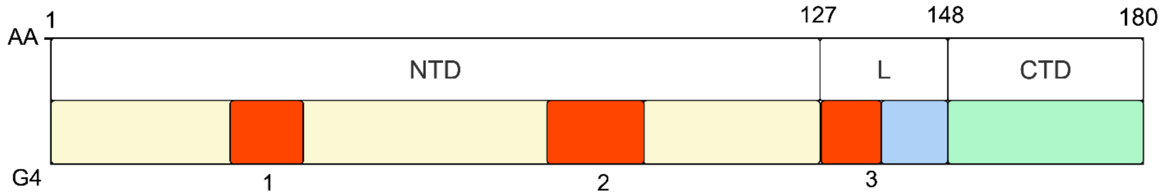


NTD – N-terminal domain. L – linker, CTD – C-terminal domain. Protein scheme as in Karousis [63]. 1 – G4 353-370, 2 – G4 509, 3 – G4 644.

**B Nsp2**

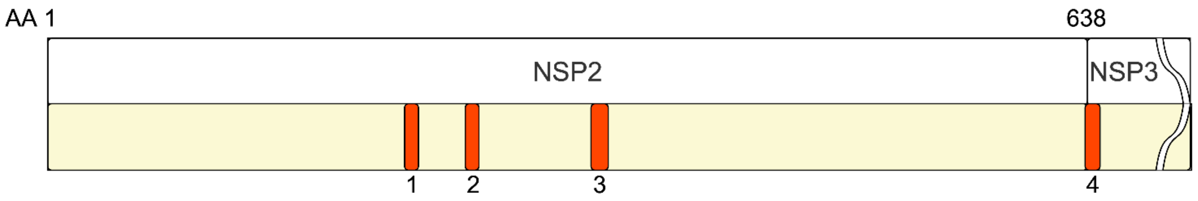


1 – G4 1463, 2 – G4 1574, 3 – G4 1805, 4 – G4 2714.

**C Nsp3 (part)**

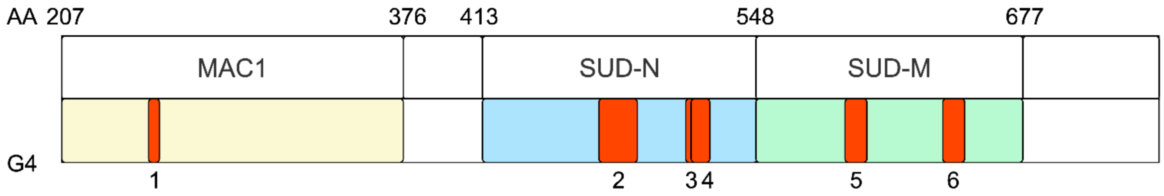


Mac1 - Macrodomain 1; SUD – SARS-unique domain. 1 – G4 3467, 2 – G4 4127, 3 – G4 4256, 4 – G4 4262, 5 – G4 4485/4487, 6 – G4 4616. Protein scheme as in Babot et al. [91].

**D Nsp4**

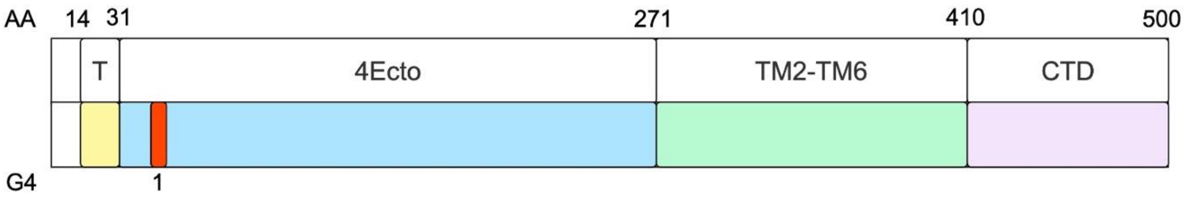


T – TM1 – transmembrane 1, 4Ecto – 4 ectodomain, TM2-TM6 – transmembrane 2-6, CTD – C-terminal domain. 1 - G4 8687. Protein scheme as in Huang et al. [92].

**E Nsp5**

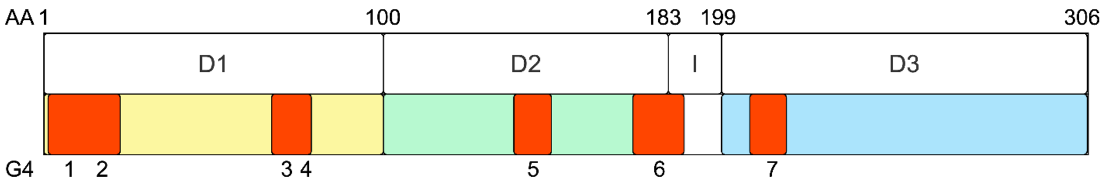


D1-D3 – domains 1-3; I – IDL – interdomain loop. 1/2 and 3/4 are overlapping PQSs. 1 – G4 10058, 2 – G4 10085, 3 – G4 10254, 4 – G4 10260, 5 – G4 10466, 6 – G4 10573, 7 – G4 10674. Protein scheme as in Roe et al. [81].

**F Spike**

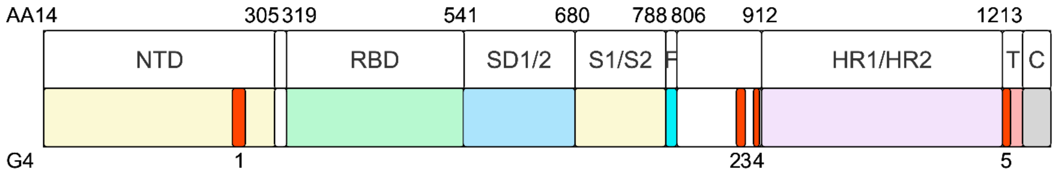


NTD – N-terminal domain, RBD – Receptor binding domain, SD1/SD2 – Subdomains 1 and 2, F – FP - Fusion peptide, HR1/HR2 – Heptapeptide repeat sequence 1 and 2, T – TM - Trans membrane (AA 1213-1237), C - CT - Cytoplasmic tail (1237-1273). Protein scheme as in Rajpal et al. [83]. 1 - G4 22316, 2 - G4 24200, 3 - G4 24215, 4 – G4 24268, 5 – G4 25197.

**G Nucleocapsid**

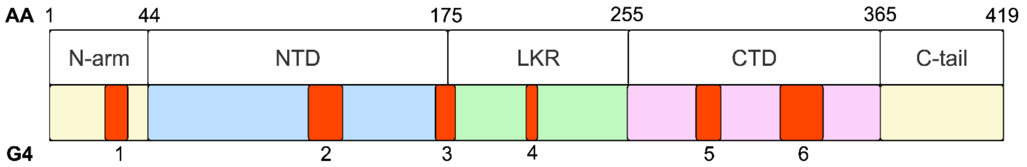


NTD – N-terminal domain (RNA-binding domain). LKR -linker region. CTD – C-terminal domain (dimerization domain, RNA-binding domain). Protein scheme as in Wu et al. [87]. 1 – G4 28346, 2 – G4 28613/28619, 3 – G4 28781, 4 – G4 28903, 5 – G4 29123, 6 – G4 29234/29254.

**Figure S3**. The domain organization of proteins in SARS-CoV-2 and G4s correspondence to the regions in them. AA – aminoacids. G4 – protein regions corresponding to G-quadruplexes. Protein domains are indicated by different colors, while those corresponding to the positions of G4s are indicated by red (see Refs. [63, 81, 83, 87, 91, 92] for details).

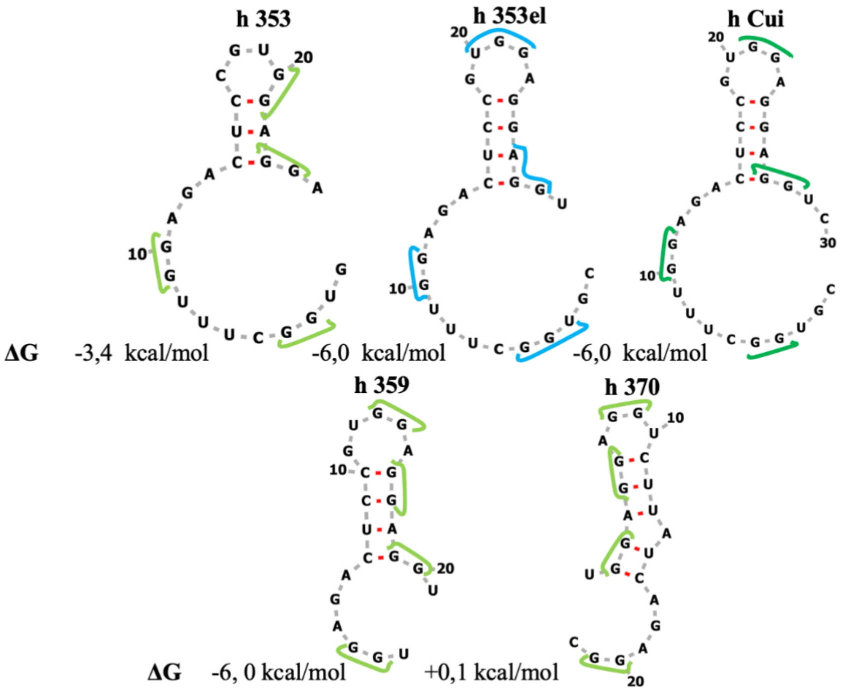


**Figure S4**. Hairpins formed by PQSs 353, 353el, 359, 370 and elongated PQS 353 (as in [22]. GG-repeats are indicated by green. Repeats in putative G4 stabilized by triad are indicated by blue. ΔG – change in free energy.

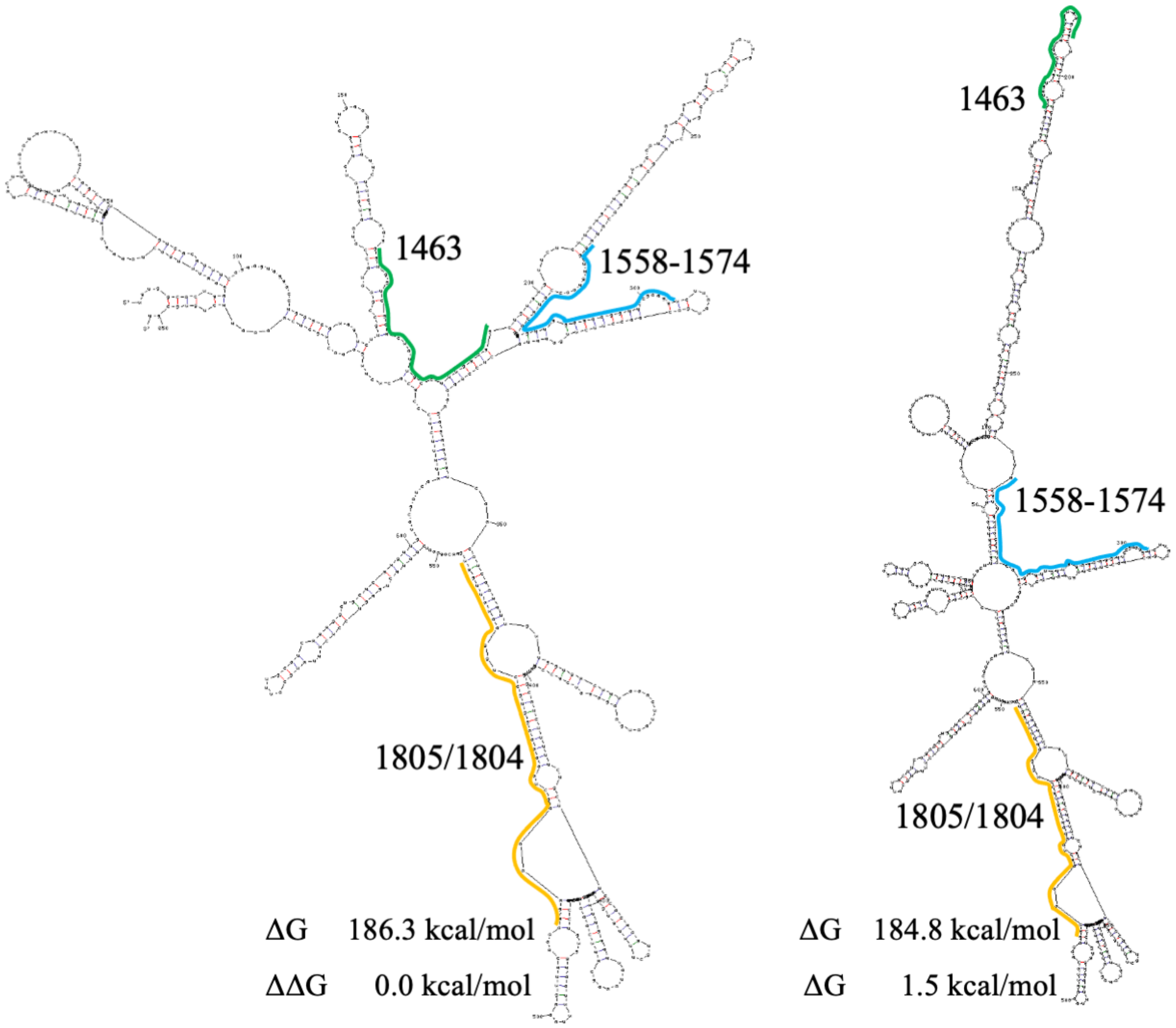


**Figure S5**. Secondary structure prediction of SARS-CoV-2 region containing PQSes 1463, 1558-1574, and 1804/1805. Based on Huston model. PQSs are underlined by green, blue and yellow, respectively. ΔG – change in free energy. ΔΔG – the energy increment of the lowest change in free energy. PQSs are indicated by lines.

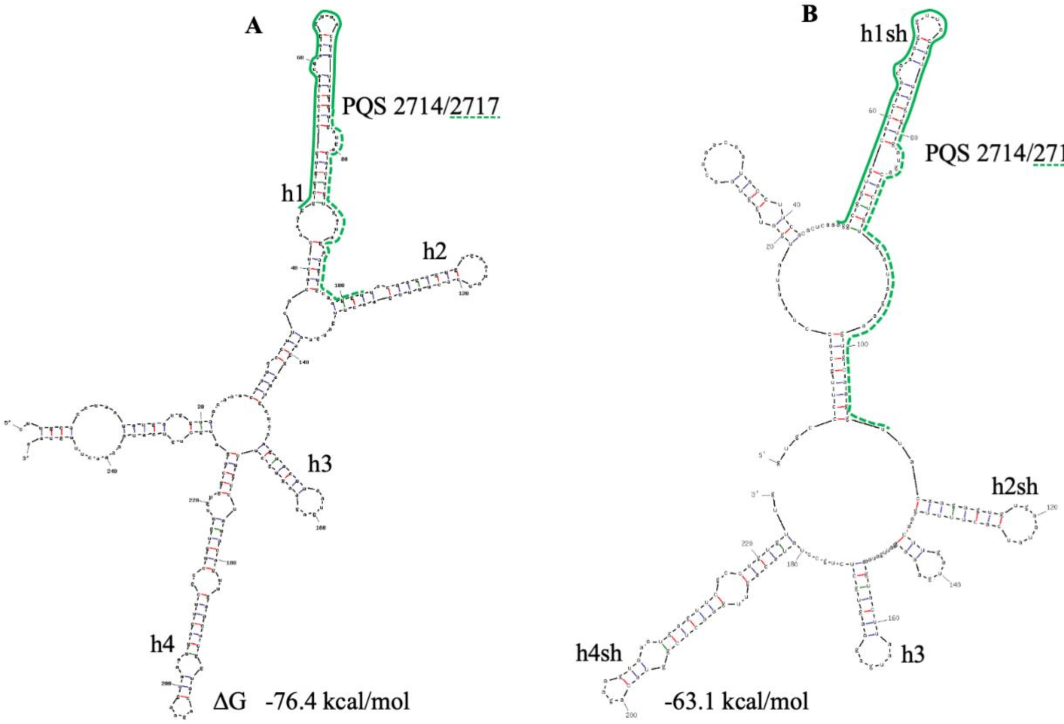


**Figure S6.** Secondary structure prediction of the SARS-CoV-2 5' region, containing PQSs 2714/2717. (A) based on the Sun model. (B) based on the Huston model. PQSs are underlined, PQS 2717 is underlined by a dotted line. Hairpins are numerated based on the Sun model; h1(2,4)sh – shortened variants of the hairpins. ΔG – change in free energy.

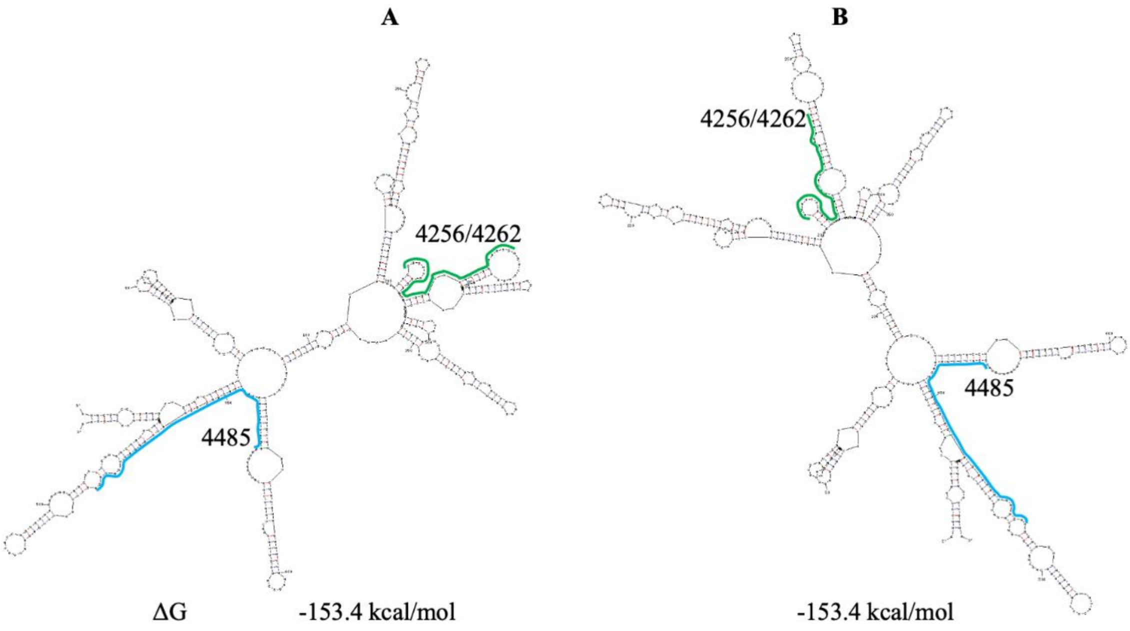


**Figure S7.** Secondary structure prediction of the SARS-CoV-2 region, containing PQSs 4256/4262 and 4485. PQSs are underlined by green and blue, respectively. (A and B) – two different optimal structures with identical ΔG. ΔG – change in free energy.

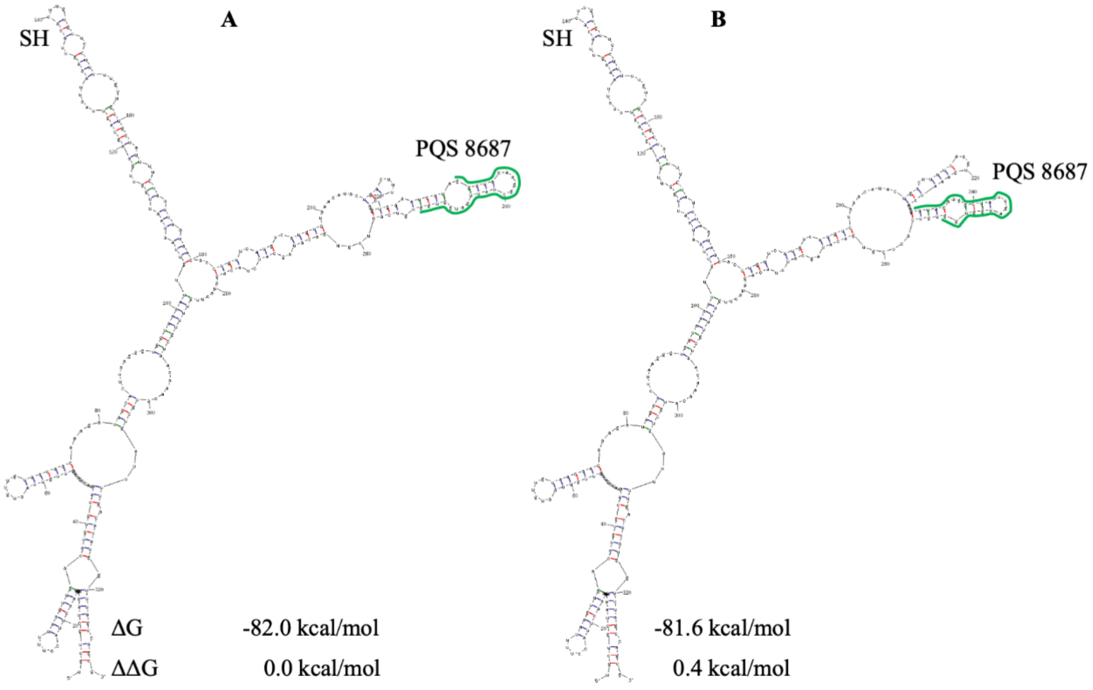


**Figure S8**. Secondary structure prediction of the SARS-CoV-2 region containing PQS 8687. PQS is underlined. ΔG – change in free energy. ΔΔG – the energy increment of the lowest change in free energy.

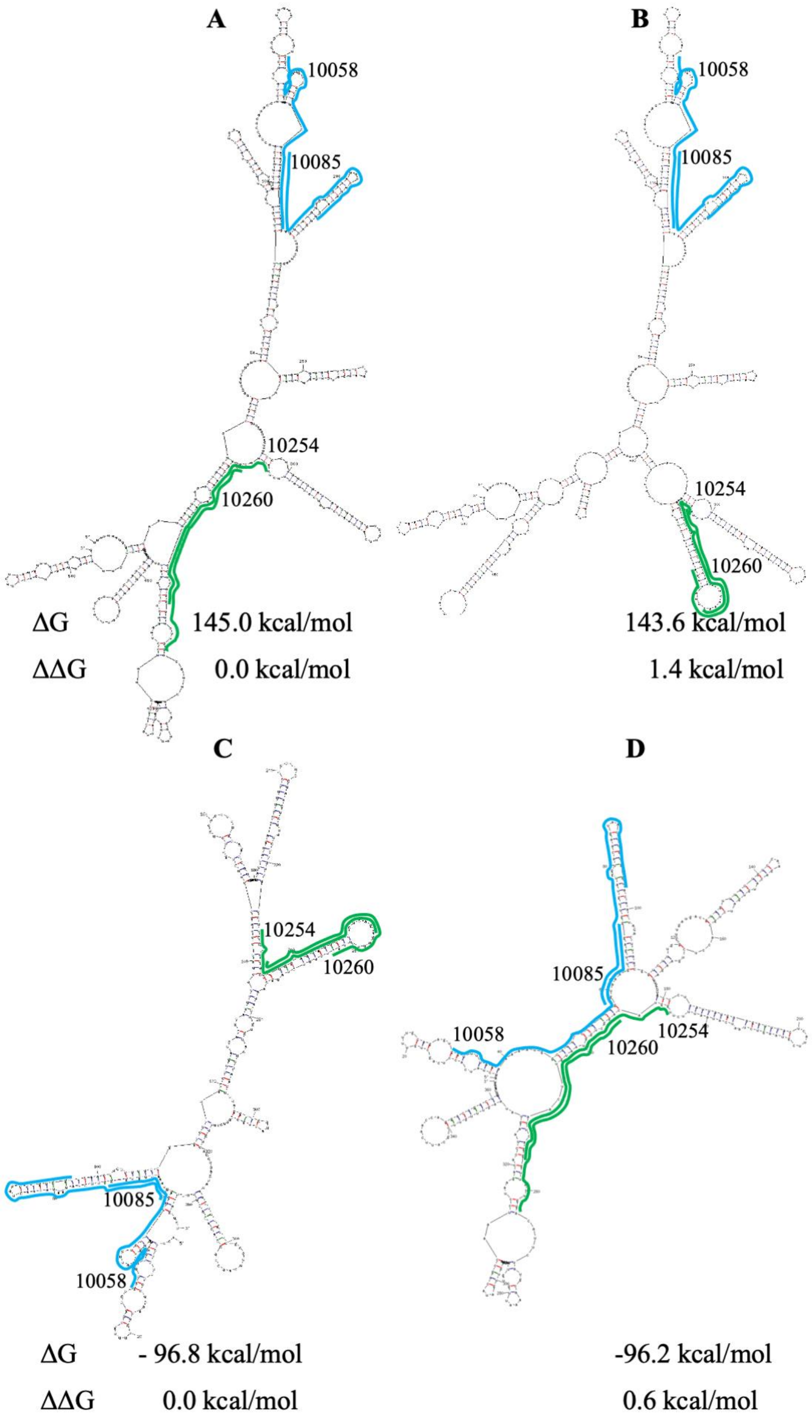


**Figure S9.** Secondary structure prediction of the SARS-CoV-2 region, containing PQSs 10058/10085 and 10254/10260. (A, B) Based on the Sun model. (C, D) Based on the Huston model. ΔG – change in free energy. ΔΔG – the energy increment of the lowest change in free energy.

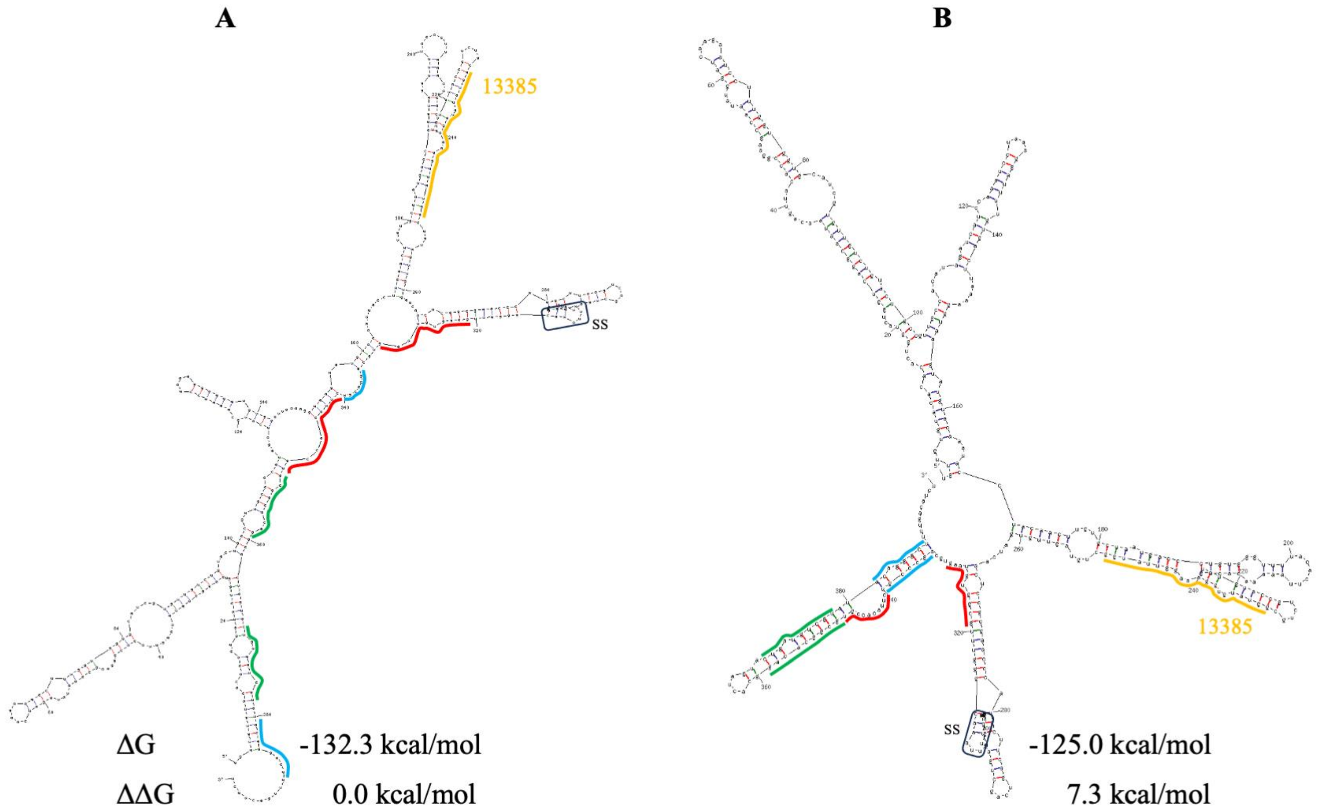


**Figure S10.** Secondary structure prediction of the SARS-CoV-2 region containing PQSes 13385 based on the Sun model. (A) wild. (B) with constraints on the folding of the structure competent for classic pseudoknot formation [93]. The arms of stem one are indicated by red. The arms of stems 2 and 3 are indicated by blue and green, respectively. Slippery sequence (SS) s indicated by a rectangle. G4 is indicated by yellow. ΔG – change in free energy. ΔΔG – the energy increment of the lowest change in free energy.

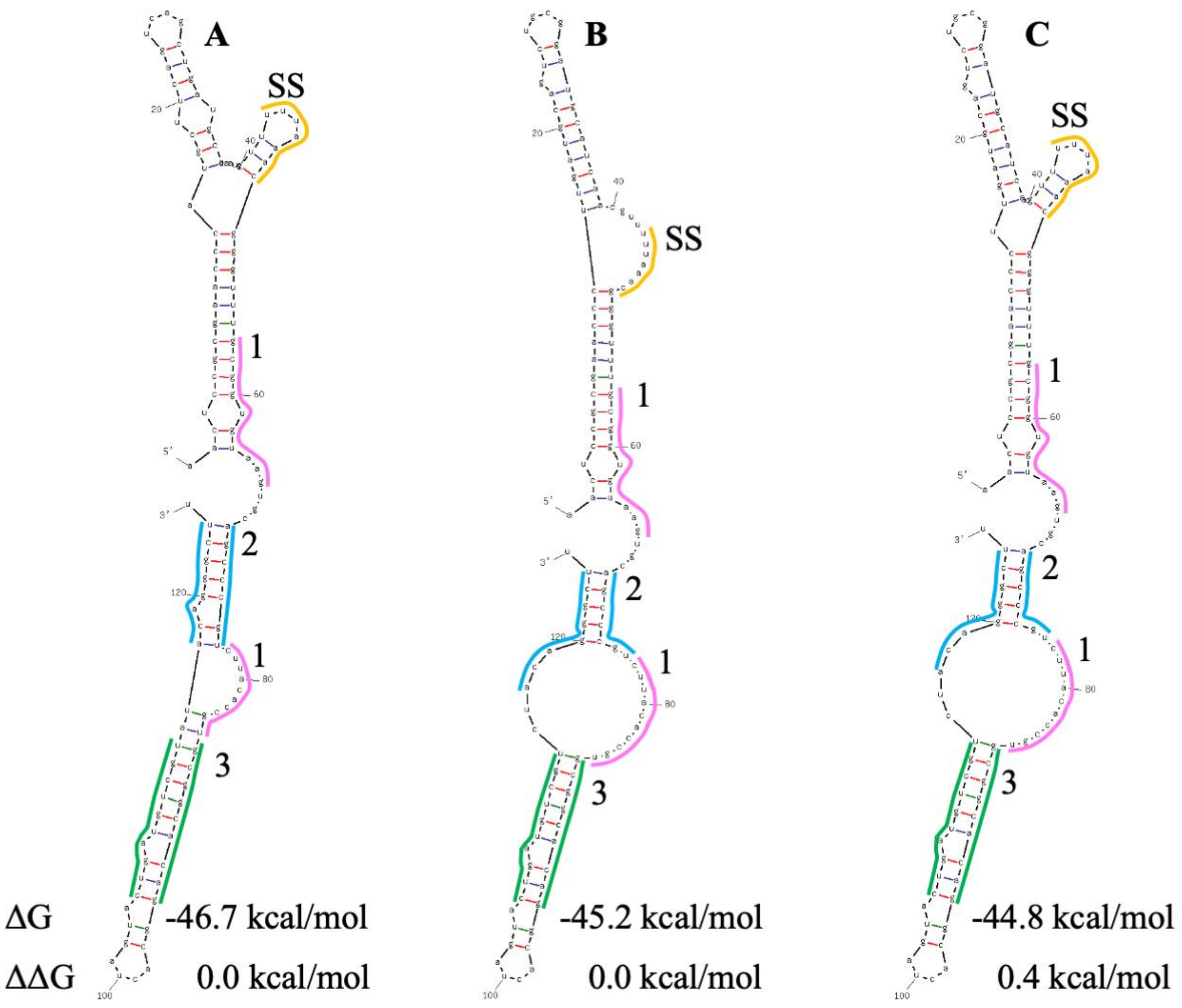


**Figure S11**. Secondary-structure prediction of the minimal RNA fragment containing the sequence of the classical pseudoknot that participates in the frameshifting process. (A) SARS-CoV-2, (B and C) SARS-CoV. Slippery site is indicated by yellow; the arms of pseudoknot duplexes are indicated by numbers. Figure is based on Kelly et al. model [93]. ΔG – change in free energy. ΔΔG – the energy increment of the lowest change in free energy.

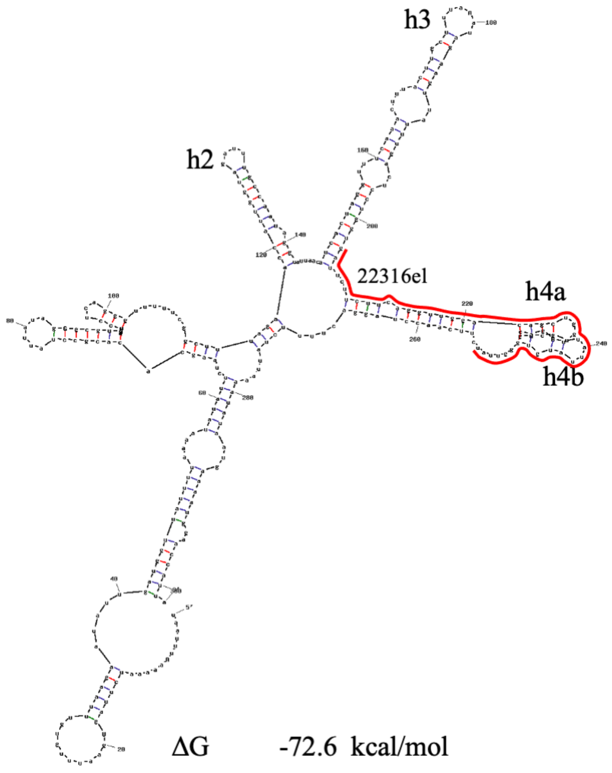


**Figure S12.** Secondary structure prediction of the SARS-CoV-2 region, containing PQS 22316el with 7 mutations. Optimal folding. PQS is indicated by line. ΔG – change in free energy.

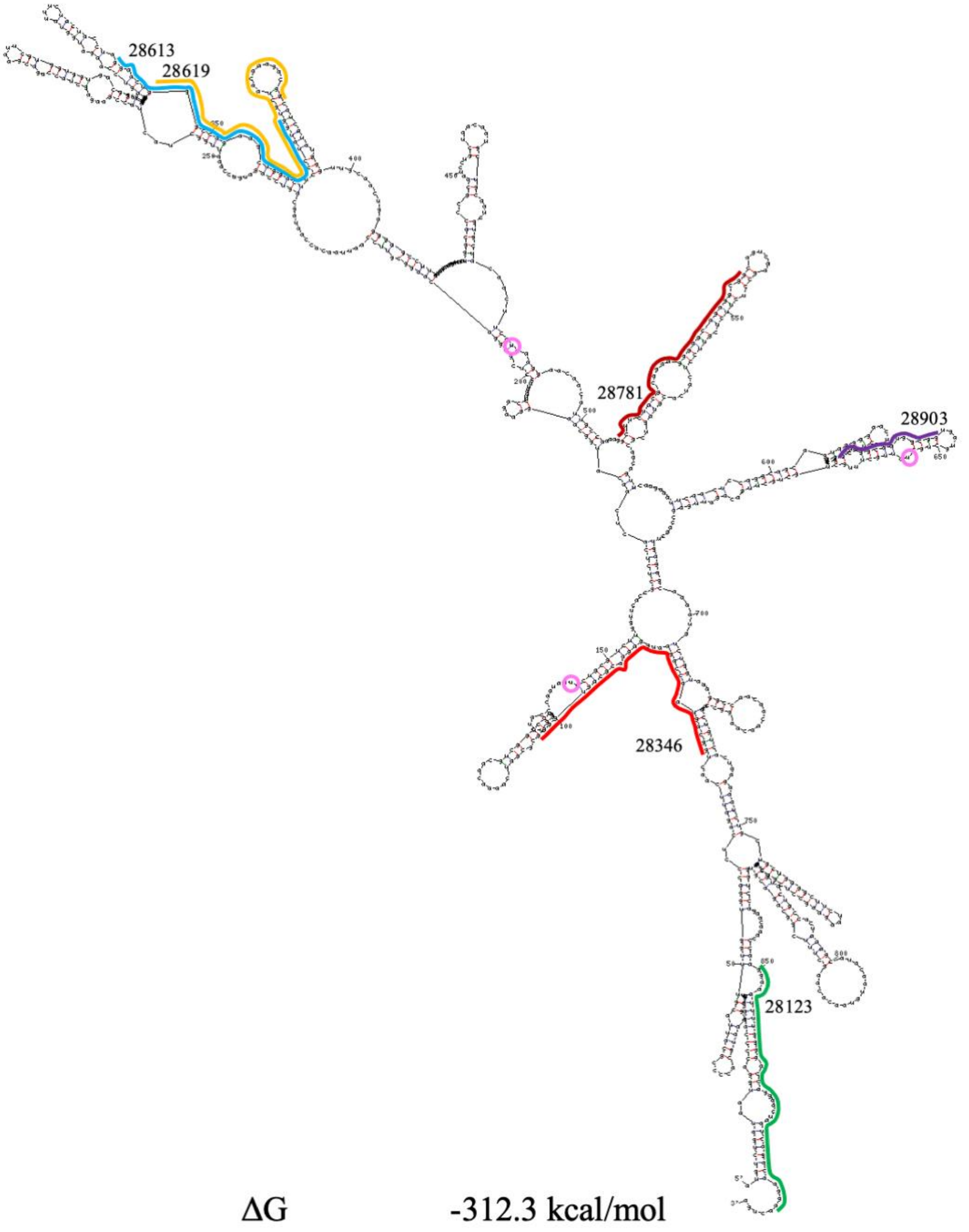


**Figure S13.** Secondary structure prediction of the SARS-CoV-2 region, containing 1st domain of the gene N RNA. ΔG – change in free energy. PQSs are indicated by a line.

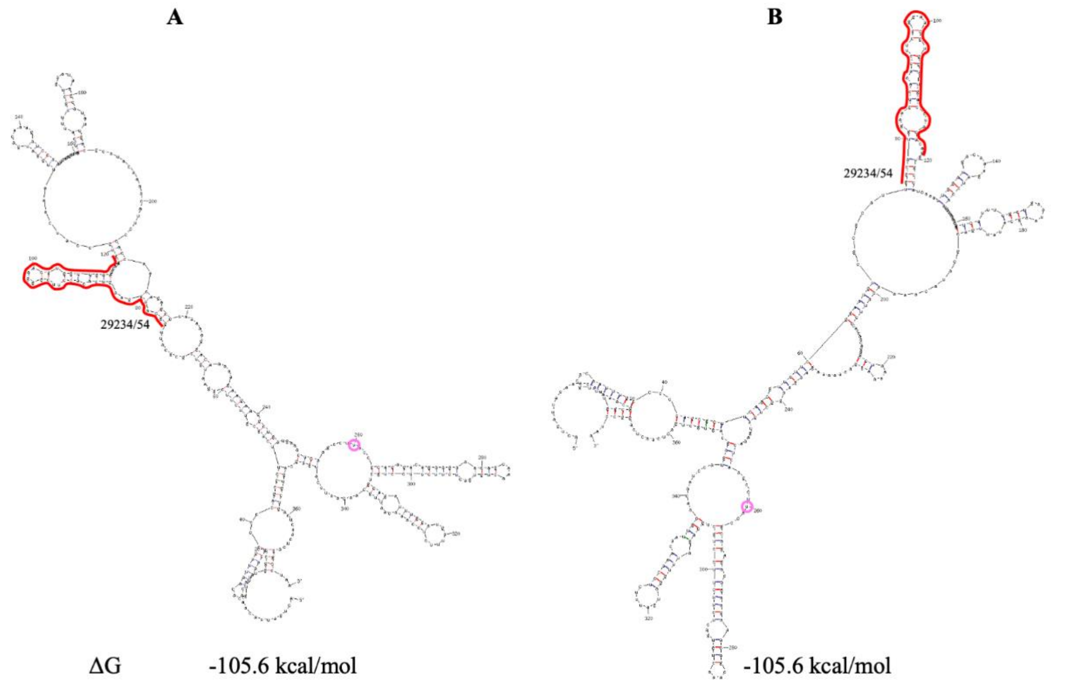


**Figure S14.** Secondary structure prediction of SARS-CoV-2 region, containing 2nd domain of gene N RNA. PQSs are indicated by line. Ψ-site is indicated by pink circle/ ΔG – change in free energy.

**PQSs**

```
28261 cgaacaaacu aaaAUGucug auaauggacc ccaaaaucag cgaaaugcac cccgcauuac
28321 guuuggugga cccucagauu caacuggcag uaaccagaau ggagaacgca guggggcgcg 28346
28381 aucaaaacaa cgucggcccc aagguuuacc caauaaUacu gcgucuuggu ucaccgcucu
28441 cacucaacau ggcaaggaag accuuaaauu cccucgagga caaggcguuc caauuaacac
28501 caauagcagu ccagaugacc aaauuggcua cuaccgaaga gcuaccagac gaauucgugg
28561 uggugacggu aaaaugaaag aucucagucc aagaugguau uucuacuacc uaggaacugg 28613
28621 gccagaagcu ggacuucccu auggugcuaa caaagacggc aucauauggg uugcaacuga
28681 gggagccuug aauacaccaa aagaucacau uggcacccgc aauccugcua acaaugcugc
28741 aaucgugcua caacuuccUc aaggaacaac auugccaaaa ggcuucuacg cagaagggag 28781
28801 cagaggcggc agucaagccu cuucucguuc cucaucacgu agucgcaaca guucaagaaa
28861 uucaacucca ggcagcagua ggggaacuuc uccugcuaga auggcuggca auggcgguga 28903
28921 ugcugcUcuu gcuuugcugc ugcuugacag auugaaccag cuugagagca aaaugucugg
28981 uaaaggccaa caacaacaag gccaaacugu cacuaagaaa ucugcugcug aggcuucuaa
29041 gaagccucgg caaaaacgua cugccacuaa agcauacaau guaacacaag cuuucggcag
29101 acguggucca gaacaaaccc aaggaaauuu uggggaccag gaacuaauca gacaaggaac 29123
29161 ugauuacaaa cauuggccgc aaauugcaca auuugccccc agcgcuucag cguucuucgg
29221 aaugucgcgc auuggcaugg aagucacacc uucgggaacg ugguugaccu acacaggugc 29234
29281 caucaaauug gaugacaaag auccaaauuu caaagaucaa gucauuuugc ugaauaagca
29341 uauugacgca uacaaaacau ucccaccaac agagccuaaa aaggacaaaa agaagaaggc
29401 ugaugaaacu caagccuUac cgcagagaca gaagaaacag caaacugugA cucuucuucc
29461 ugcugcagau uuggaugauu ucuccaaaca auugcaacaa uccaugagca gugcugacuc
29521 aacucaggcc UAAacucaug cagaccacac aaggcagaug ggcuauauaa acguuuucgc
```

**Figure S15.** Gene N, nts 28274-29533. Start and stop codons are indicated by pink. GG(GGG)-repeats are indicated by red. Pseudouridine sites are indicated by blue and shown in capital letters. The start of the second domain is indicated by yellow.

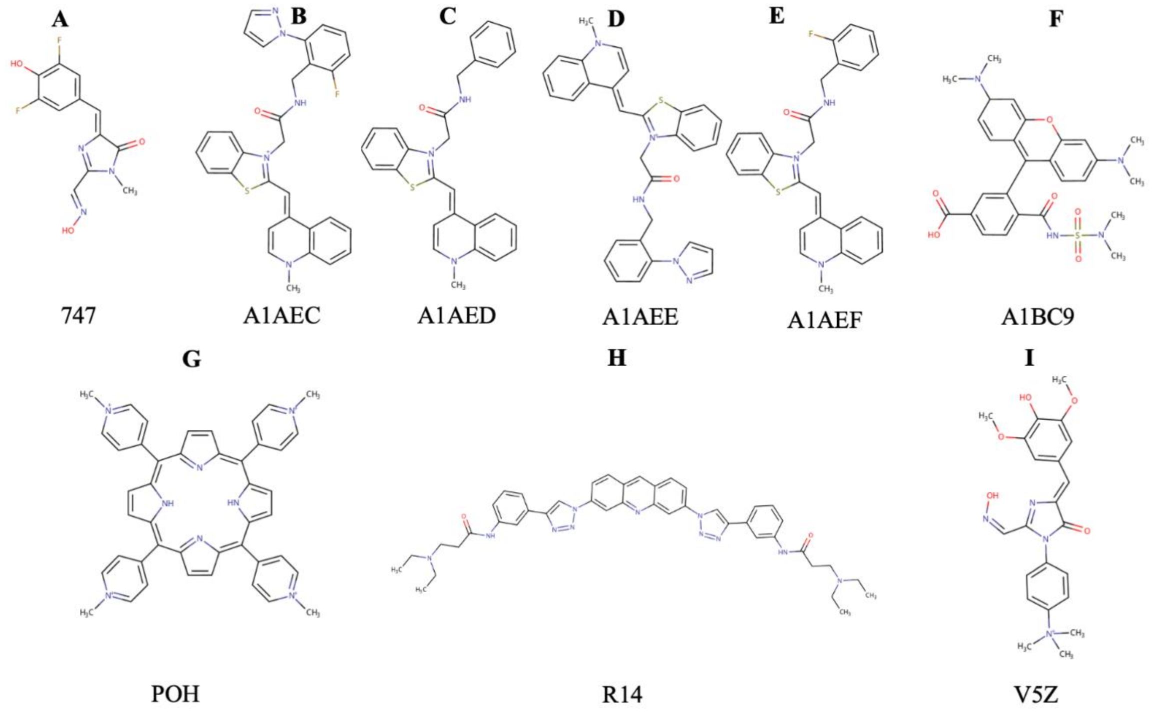


**Figure S16.** Chemical formulas of compounds.

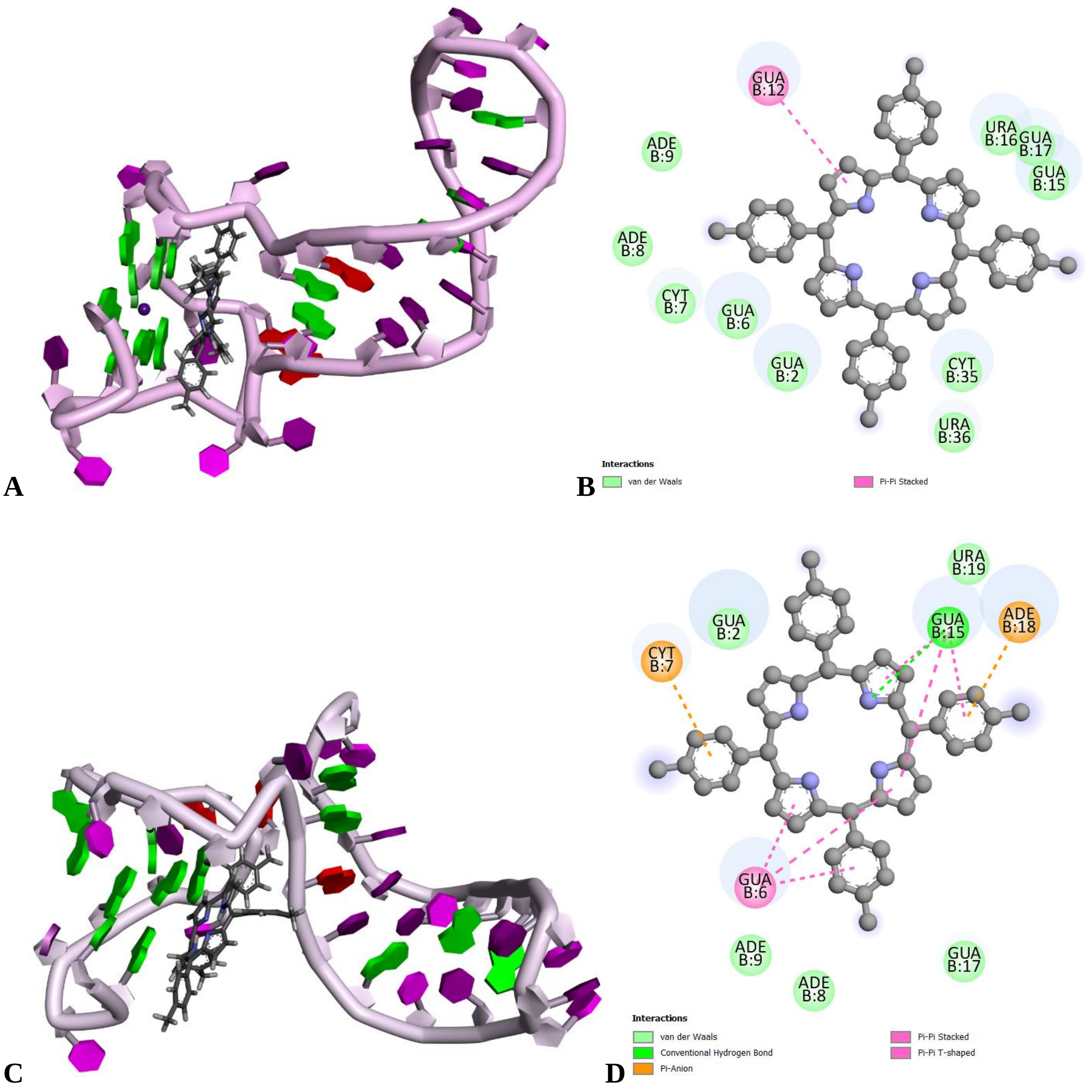


**Figure S17**. Spatial structure of the SARS-CoV-2 genomic RNA fragment nts 28903-28927 in complex with POH before (A) and after (C) molecular dynamics and 2D interaction diagram at the corresponding site before (B) and after (D) molecular dynamics.
